# Oligodendrocyte transcription factor 2 orchestrates glioblastoma immune evasion by suppressing CXCL10 and CD8^+^ T cell activation

**DOI:** 10.1172/JCI195556

**Published:** 2026-01-27

**Authors:** Xinchun Zhang, Jinjiang Xue, Cunyan Zhao, Chenqiuyue Zeng, Jiacheng Zhong, Gangfeng Yu, Xi Yang, Yao Ling, Dazhen Li, Jiaxiao Yang, Yun Xiu, Hongda Li, Shiyuan Hong, Liangjun Qiao, Song Chen, Q. Richard Lu, Yaqi Deng, Zhaohua Tang, Fanghui Lu

**Affiliations:** 1 Department of Cancer Center, The Second Affiliated Hospital of Chongqing Medical University, Chongqing, China.; 2 Department of Neurosurgery, Key Laboratory of Major Brain Disease and Aging Research (Ministry of Education), The First Affiliated Hospital of Chongqing Medical University, Chongqing, China.; 3 School of Basic Medical Sciences, Chongqing Medical University, Chongqing, China.; 4 Chongqing University Central Hospital, Chongqing, China.; 5 College of Pharmacy, Chongqing Medical University, Chongqing, China.; 6 Department of Pediatrics, Division of Experimental Hematology and Cancer Biology, Cincinnati Children’s Hospital Medical Center, Cincinnati, Ohio, USA.

**Keywords:** Cell biology, Immunology, Oncology, Brain cancer

## Abstract

Glioblastomas (GBMs) are highly lethal brain tumors with limited treatment options and resistance to immune checkpoint inhibitors due to their immunosuppressive tumor microenvironment. Here, we identify OLIG2 as a key regulator of immune evasion in GBM stem-like cells, which inhibits CD8^+^ T cell–dependent antitumor immunity while promoting protumor macrophage polarization. Mechanistically, OLIG2 recruited HDAC7 to repress *CXCL10* transcription, inducing STAT3 activation in tumor-associated macrophages (TAMs) and decreasing CD8^+^ T cell infiltration and activation. Genetic deletion of *OLIG2* significantly increased CXCL10 secretion, shifting TAMs toward an antitumor phenotype and enhancing CD8^+^ T cell activities. Furthermore, upregulated OLIG2 expression was correlated with resistance to immune checkpoint inhibitors in patients with GBMs. OLIG2 inhibition by either genetic deficiency or pharmacological targeting with CT-179 sensitized GBM tumors to anti–PD-L1 therapy, enhancing antitumor immune responses and prolonging survival. Our findings reveal OLIG2^+^ glioma stem-like cells as critical mediators of immune evasion and identify the OLIG2/HDAC7/CXCL10 axis as a potential therapeutic target to enhance immune checkpoint inhibitor efficacy and improve immunotherapy outcomes in aggressive GBMs.

## Abstract 2

OLIG2, a master transcription factor in glioblastoma stem cells, enables immune evasion by suppressing CXCL10. Targeting OLIG2 overcomes immunotherapy resistance and improves survival.

## Graphical abstract

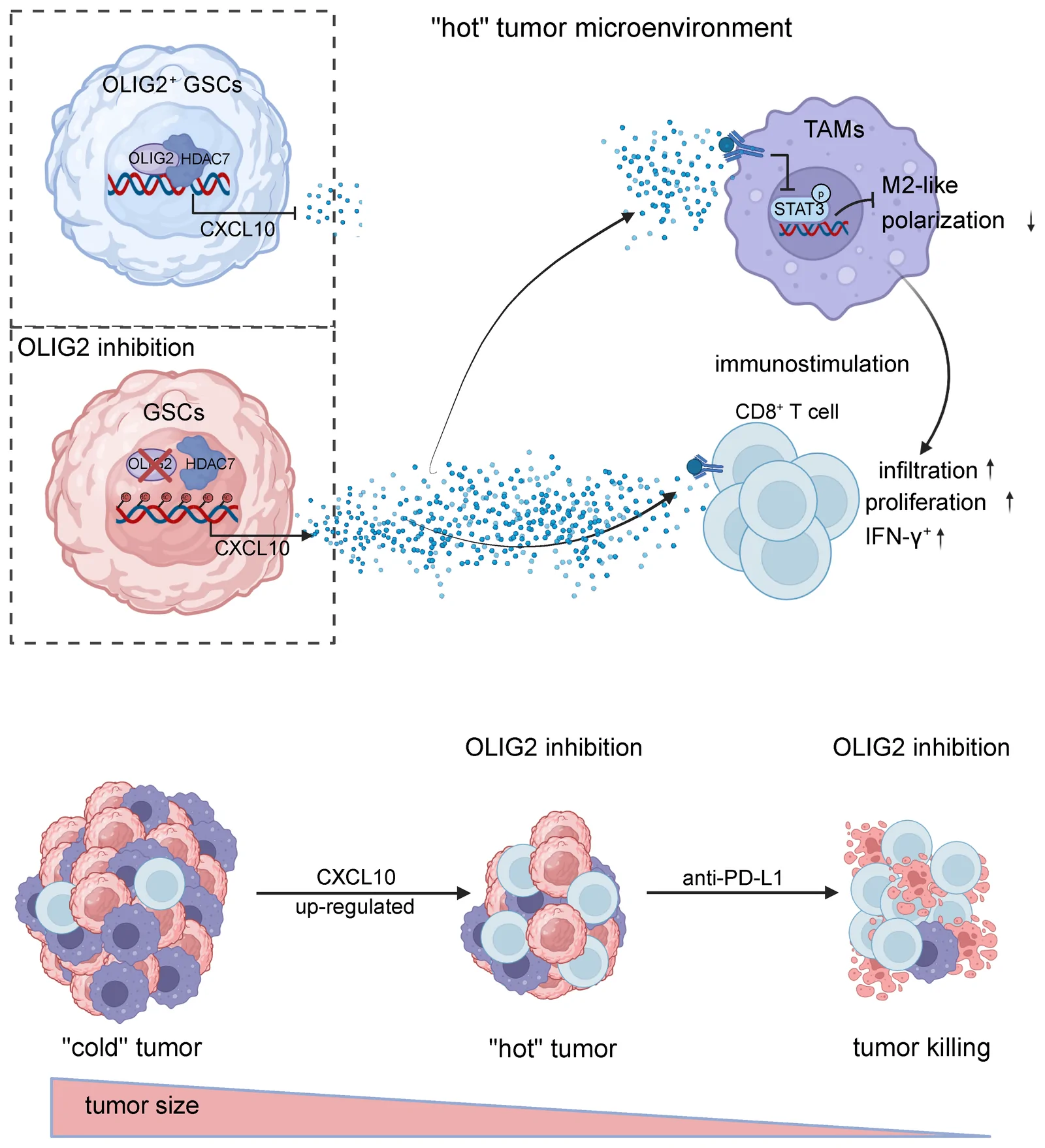

## Introduction

Glioblastomas (GBMs) are the most common and lethal primary brain tumors in adults, with a median survival of less than 15 months and a 5-year survival rate of just approximately 7% (1). Beyond radiotherapy and temozolomide following surgical resection, advanced therapeutic options remain limited (2). While immune checkpoint inhibitors (ICIs) have shown success in treating melanoma and non–small cell lung cancer, their efficacy in GBMs is hindered by the tumors’ highly immunosuppressive microenvironment (3–5). This complex tumor microenvironment (TME) is shaped by dynamic interactions between heterogeneous tumor cells and other components, including endothelial cells, astrocytes, and immune cells (6, 7).

Glioblastoma stem-like cells (GSCs) play a crucial role in tumor progression, possessing robust self-renewal and tumor-initiating capacities (8, 9). While extensively studied for their contributions to GBM development, their role in shaping the TME remains poorly characterized. The GBM-specific transcription factor OLIG2 is a key regulator of tumorigenesis, driving proliferation by activating cell cycle regulators and oncogenic transcription programs (10, 11). Previous studies have demonstrated that OLIG2 reprograms differentiated GBM cells into propagating stem-like cells (12). Additionally, OLIG2^–^ glioma cells induce innate immune activation via angiogenesis and blood-brain barrier disruption (13). Other transcription factors, such as SOX2 and OCT4, also contribute to GBM progression by regulating BRD-dependent immunosuppressive transcriptional programs, further implicating OLIG2^+^ GSCs in establishing an immunosuppressive TME (14).

Substantial progress has been made in understanding how GSCs evade immune surveillance. For instance, GSCs suppress T cell activity by downregulating MHC molecules and antigen-processing factors (15). Additionally, GSC-derived exosomes manipulate myeloid-derived suppressor cells to inhibit T cell activation (16). GSCs can also inhibit CD8^+^ T cell infiltration and antitumor immune response by reprogramming lysine catabolism (17). Furthermore, GSCs interact with tumor-associated macrophages (TAMs), the most abundant immune cells in GBMs, by secreting chemoattractants such as CXCL8, CSF1, POSTN, TFPI2, and WISP1, leading to the polarization of TAMs into protumor, immunosuppressive phenotypes (18–22). While evidence clearly supports the ability of GSCs to reshape the TME, the precise molecular mechanisms governing these interactions remain largely unknown.

Chemokines are well-known regulators of immune cell activity and play key roles in modulating the GBM TME. In IDH-mutant gliomas, reduced CXCL9 and CXCL10 expression correlates with lower STAT1 levels, impairing CD8^+^ T cell migration (23). Notably, CXCL9 delivery via adenoviral vectors enhances cytotoxic T cell infiltration, sensitizing GBMs to anti–PD-1 therapy (24). Conversely, recent studies indicate that elevated CXCL10 expression can recruit immunosuppressive CNS myeloid cells, promoting brain metastasis (25). The regulatory mechanisms and dual roles of chemokines in the GBM immune landscape remain a major challenge in the field.

In this study, we demonstrate that OLIG2^+^ GSCs actively suppress the immune microenvironment by promoting antiinflammatory TAM polarization and inhibiting CD8^+^ T cell infiltration and activation. Mechanistically, we show that OLIG2 recruits HDAC7 to suppress CXCL10 transcription, thereby silencing TAM-mediated and CD8^+^ T cell–driven antitumor responses, ultimately shaping a cold immune microenvironment. Furthermore, we evaluate the therapeutic efficacy of targeting OLIG2 in combination with anti–PD-L1 therapy, demonstrating that genetic or pharmacological inhibition of OLIG2 enhances antitumor immunity and significantly prolongs survival in tumor-bearing mice. Our findings highlight OLIG2^+^ GSCs as key drivers of immune evasion in GBMs and reveal the OLIG2/HDAC7/CXCL10 axis as a promising therapeutic target for overcoming ICI resistance, offering new insights into immunotherapy strategies for patients with GBMs.

## Results

### High OLIG2 expression positively correlates with the immunosuppressive TME in GBMs.

**Figure 1:**
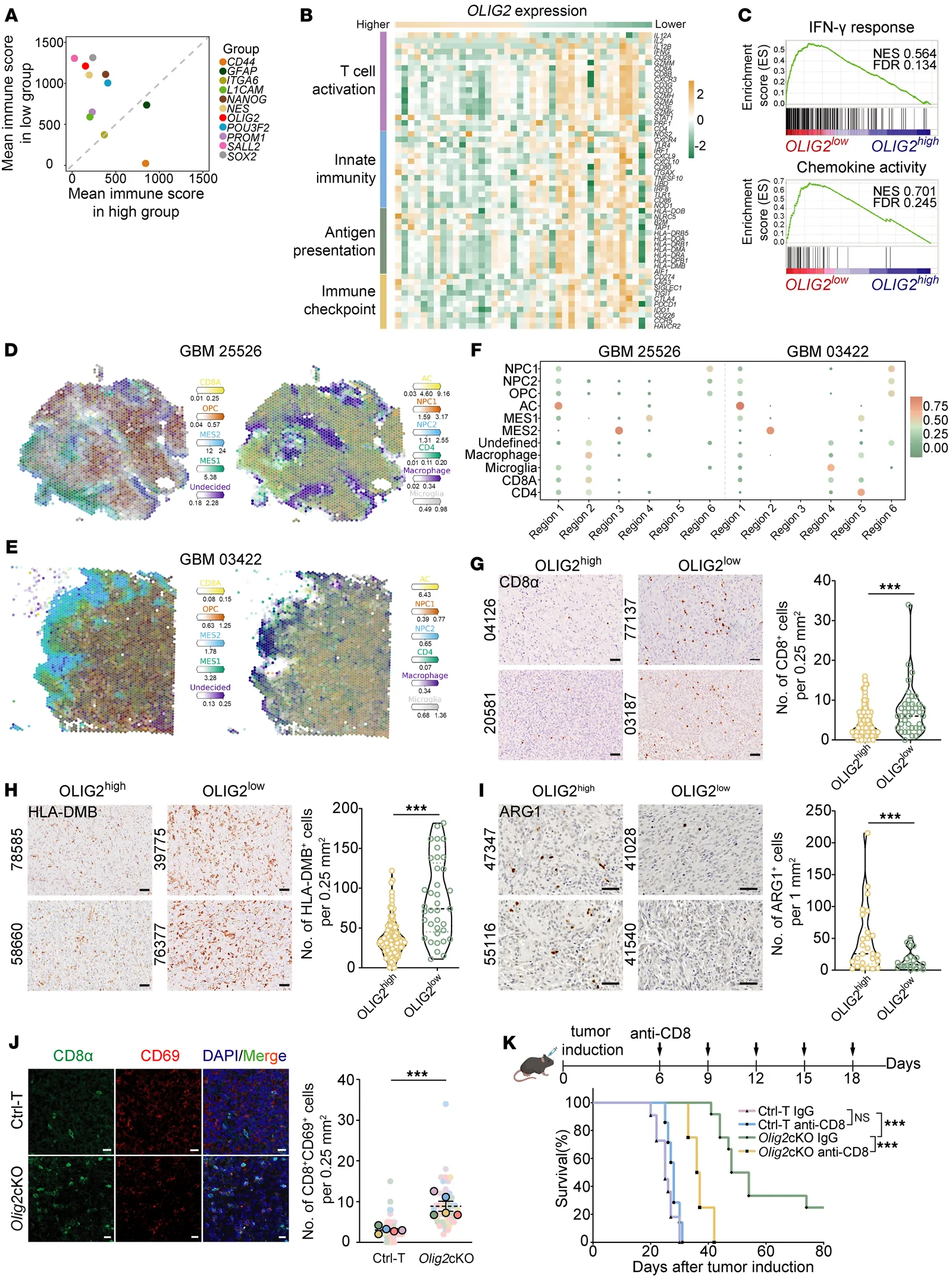
High OLIG2 expression positively correlates with the immunosuppressive TME in GBMs. (**A**) ESTIMATE analysis of immune scores in patients with GBMs from TCGA database with high or low expression of GSC markers (*n* = 40). (**B**) Heatmap showing the relative expression of signature immune genes in patients with GBMs from TCGA database (*n* = 40). (**C**) GSEA showing the enrichment of signature gene sets of immunoregulation in *OLIG2*^lo^ GBM cases compared with *OLIG2*^hi^ specimens (*n* = 40). (**D**–**F**) Spatial mapping of cell types and quantifications in GBM tissues utilizing the cell2location method. Estimated cell abundance is depicted by color intensity for indicated cell types (**D** and **E**). Non-negative matrix factorization analysis of GBM tissue sections reveals cellular compartment abundance (**F**). Dot size and color represent the weight and normalized cell abundance. (**G**–**I**) Representative images of IHC staining and quantifications of CD8α (*n* = 164), HLA-DMB (*n* = 164), or ARG1 (*n* = 46) in GBM cases with high or low OLIG2 expression. Each plot represents the number of CD8α, HLA-DMB, or ARG1 cells per area (0.25 or 1 mm^2^). Scale bars: 50 μm. (**J**) IF images and quantification of relative CD8^+^CD69^+^ T cells in Ctrl-T and *Olig2*cKO mice (*n* = 5 independent mice). Scale bars: 20 μm. (**K**) Schematic and Kaplan-Meier survival curve of Ctrl-T or *Olig2*cKO GBM mice treated with anti-CD8α antibody (10 mg/kg). Log-rank test was used to assess significance (*n* = 7–12 mice/group). Statistical significance was determined by unpaired 2-tailed Student’s *t* test in **G**–**J**. ***P < 0.001.

Emerging evidence suggests that GSCs dynamically inﬂuence and communicate with the GBM tumor immune microenvironment (9). To identify the factors that contribute to GSC-immune cell symbiosis, we examined the correlation of immune scores in patients with GBMs using a series of GSC markers and the ESTIMATE algorithm (26). We identified *SOX2*, *OLIG2*, *SALL2*, and *CD44* as key regulators influencing immune scores in GBM samples from The Cancer Genome Atlas (TCGA) database (Figure 1A and Supplemental Figure 1A; supplemental material available online with this article; https://doi.org/10.1172/JCI195556DS1). Notably, *OLIG2* is highly coexpressed with the GSC markers *SOX2* and *SALL2* (Supplemental Figure 1B). Given its essential role in GBM initiation and progression, we focused on *OLIG2* to investigate its contribution to immunosuppression in GBMs.

Patients with GBMs with high *OLIG2* expression (*OLIG2*^hi^) exhibited significantly lower immune and stromal scores compared with those with low *OLIG2* expression (*OLIG2*^lo^) (Supplemental Figure 1C). To define the differential TME shaped by *OLIG2* in GBMs, we used CIBERSORT-based immune deconvolution to identify *OLIG2*^hi^ GBMs associated with a diminished immunostimulatory landscape, characterized by decreased infiltration of CD8^+^ T cells and activated CD4^+^ T cells, as well as an accumulation of M0 macrophages (Supplemental Figure 1D). Transcriptome profiling further supported this immunosuppressive phenotype, showing downregulation of key immune-related pathways in *OLIG2*^hi^ GBMs, including T cell activation, innate immunity signaling, antigen presentation machinery, and immune checkpoint regulation (Figure 1B). GSEA revealed marked enrichment of antitumor immune pathways in the *OLIG2*^lo^ group, including IFN-γ response, chemokine activity, antigen processing and presentation, and inflammatory response (Figure 1C and Supplemental Figure 1E).

### Spatial associations between OLIG2 expression and immune cells in GBM tissues.

To further investigate the spatial associations between *OLIG2* expression and immune cell populations, we integrated scRNA-seq data with Visium spatial transcriptomic data from 2 representative patients with IDH-WT GBMs using the cell2location algorithm for precise cell type mapping (27). Spatial transcriptomic profiles revealed distinct regions corresponding to 7 tumor cell clusters, TAMs, and T cells (28) (Supplemental Figure 1F). Among them, astrocyte-like (AC), mesenchymal (MES), and other undefined tumor cell populations showed pronounced spatial proximity to T cell–enriched regions (Figure 1, D–F, and Supplemental Figure 1G). Notably, these tumor cell populations were characterized by low *OLIG2* expression, suggesting a potential niche-specific crosstalk between *OLIG2*^lo^ populations and tumor-infiltrating T cells (Supplemental Figure 1F). Furthermore, CellChat analysis through scRNA-seq revealed that *OLIG2*^–^*SOX2*^+^-labeled GSCs established enhanced ligand–receptor interaction networks with T cells and TAMs compared with *OLIG2*^+^ counterparts (Supplemental Figure 1H). These findings suggest that *OLIG2*^hi^ and *OLIG2*^lo^ GSCs differentially regulate immune cell dynamics within the TME. To validate these findings, we performed immunostaining on a cohort of GBM specimens and observed a significantly higher proportion of CD8α^+^ T cells and HLA-DMB^+^ cells in OLIG2^lo^ patients, whereas the OLIG2^hi^ group exhibited more ARG1^+^ populations (Figure 1, G–I). By contrast, the CD4^+^ T cells population remained unchanged (Supplemental Figure 1I).

### Correlation of OLIG2^+^ GSCs with an immunosuppressive TME in a GBM animal model.

Next, we investigated whether the immunosuppressive environment in GBMs was driven by OLIG2^+^ GSCs. To this end, we utilized a spontaneous immunocompetent murine GBM model, as previously described (11), enabling conditional knockout of *Olig2* in GSCs. This was achieved by selectively ablating floxed alleles of *Pten* and *Trp53* in combination with *Olig2*^fl/fl^ alleles (*Olig2*cKO). GSEA of bulk RNA-seq from tumor tissues of *Olig2*^fl/fl^ mice revealed enhanced immune signaling pathways, including LPS-stimulated macrophage activation and interferon response (Supplemental Figure 1J). We found that tumors in *Olig2*cKO mice exhibited an increased population of activated CD8^+^ T cells (CD8^+^CD69^+^) (Figure 1J). Furthermore, depletion of CD8^+^ T cells using an anti-CD8α antibody significantly accelerated tumor progression in *Olig2*cKO mice compared with the isotype control. By contrast, WT tumor–bearing control mice showed no change in survival following anti-CD8α antibody treatment. This observation suggests that OLIG2 plays a key role in suppressing CD8^+^ T cell activity, thereby contributing to tumor progression (Figure 1K and Supplemental Figure 1K). Together, these findings indicate that a large OLIG2^+^ GSC population is associated with a more immunosuppressive protumor microenvironment.

### OLIG2 deficiency reprograms the TME to facilitate antitumor immunity in GBMs.

**Figure 2:**
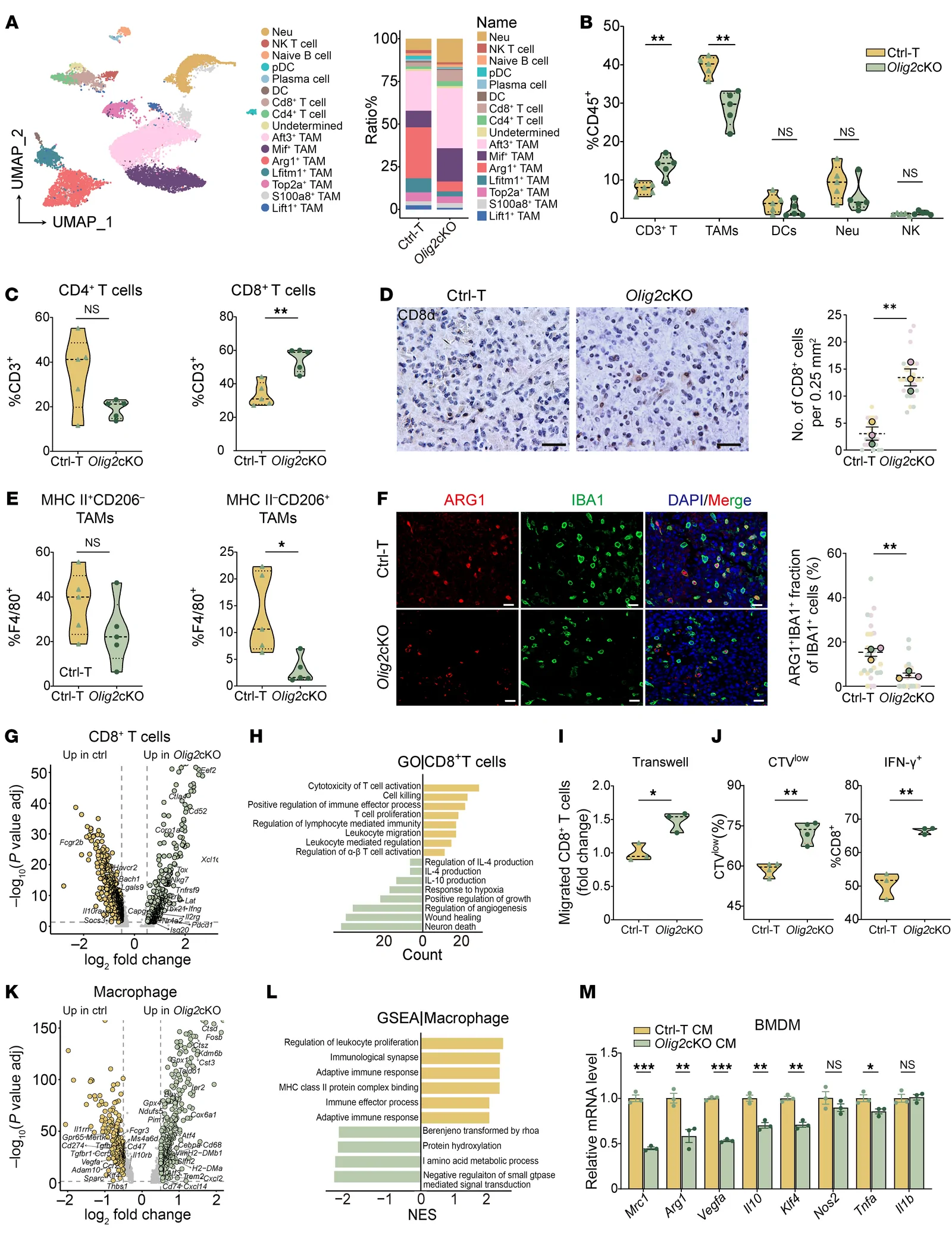
OLIG2 deletion enhances T cell activation and inhibits protumor TAMs in GBMs. (**A**) UMAP of scRNA-seq data from CD45^+^ cells in Ctrl-T and *Olig2*cKO mice (*n* = 4/group) (left) and the frequencies for subtypes from both groups (right). (**B**) Flow cytometry quantification of the CD3^+^ T cells, TAMs, DCs, neutrophils, and NK cells in tumor-bearing Ctrl and *Olig2*cKO mice (*n* = 5/group). (**C**) Flow cytometry quantification of the CD8^+^ and CD4^+^ T cell populations in tumor-bearing Ctrl and *Olig2*cKO mice (*n* = 5/group). (**D**) Representative staining and quantification for CD8α in tumor-bearing Ctrl and *Olig2*cKO mice. Scale bars: 20 μm. Each plot represents the number of CD8α per area (0.25 mm^2^) (*n* = 3/group). (**E**) Flow cytometry quantifications of the MHC II^–^CD206^+^ and MHC II^+^CD206^–^ TAMs in tumor-bearing Ctrl and *Olig2*cKO mice (*n* = 5/group). (**F**) Representative staining and quantification for ARG1 (red) and IBA1 (green) in tumor tissues from Ctrl-T and *Olig2*cKO mice (*n* = 3/group). Scale bars: 20 μm. (**G**) Volcano plots of differentially expressed genes for CD8^+^ T cells between Ctrl-T and *Olig2*cKO mice. (**H**) GSEA of differentially expressed genes for CD8^+^ T cells between *Olig2*cKO and Ctrl-T. (**I**) Quantification of migrated CD8^+^ T cells toward Ctrl-T and *Olig2*cKO CM by transwell assay (*n* = 3). (**J**) Flow cytometry quantification of the proliferation (*n* = 4) and IFN-γ expression (*n* = 3) in CD8^+^ T cells cocultured with Ctrl-T and *Olig2*cKO CM. (**K**) Volcano plots of differentially expressed genes for macrophages between Ctrl-T and *Olig2*cKO mice. (**L**) GSEA of differentially expressed genes for macrophages between *Olig2*cKO and Ctrl-T. (**M**) qPCR of mRNA expression of selected antiinflammatory and proinflammatory markers in BMDMs treated with Ctrl-T and *Olig2*cKO CM for 48 hours (*n* = 3). Data are shown as mean ± SEM. Statistical significance was determined by unpaired 2-tailed Student’s *t* test in **B**–**M**. *P < 0.05, **P < 0.01, ***P < 0.001.

To investigate how OLIG2 reprograms the TME, we performed scRNA-seq to analyze immune cell profiles sorted from both control (Ctrl-T) and *Olig2*cKO tumors. Unsupervised clustering using UMAP identified 15 distinct cell types based on their canonical gene markers. Compared with Ctrl-T tumors, *Olig2*cKO tumors exhibited an obvious increase in CD8^+^ T cell populations and a marked reduction in Arg1^+^ TAMs (Figure 2A and Supplemental Figure 2A).

Flow cytometry analysis of infiltrating immune cells in *Olig2*cKO tumors showed an increased T cell fraction (CD3^+^) alongside a concomitant decrease in TAMs (CD11b^+^F4/80^+^). However, no significant changes were observed in the populations of DCs (CD11c^+^MHC II^+^CD11b^–^), neutrophils (CD11b^+^Ly6G^+^F4/80^–^), or NK cells (CD3^–^NK1.1^+^) (Figure 2B, Supplemental Figure 2B, and Supplemental Figure 5D). In the CD3^+^ fraction, *Olig2*cKO tumors exhibited a notable increase in CD8^+^ T cells (Figure 2C and Supplemental Figure 2C), which was further confirmed by immunostaining (Figure 2D). Within the TAM populations, the reduction in antiinflammatory macrophages (MHCII^–^CD206^+^, M2-like) was particularly pronounced in *Olig2*cKO tumors (Figure 2E). IHC further revealed a significant reduction in ARG1^+^IBA1^+^ TAMs within the tumor lesions of *Olig2*cKO mice (Figure 2F). These findings suggest a shift in the TME toward a less immunosuppressive state in *Olig2*cKO tumors.

To investigate the impact of OLIG2 on the transcriptional profiles of CD8^+^ T cell subsets, we analyzed gene expression changes in *Olig2*cKO tumors. We observed upregulation of effector markers (*Ifng*, *Tbx21*, *Isg20*, and *Xcl1*) and exhaustion markers (*Pdcd1*, *Tox*, and *Ctla4*) in CD8^+^ T cells, whereas the suppressive regulators (*Il10ra* and *Lgals9*) were downregulated (Figure 2G). Consistently, cellular pathways related to T cell activation, migration, and cytotoxicity were enriched in the CD8^+^ T cell population from *Olig2*-deficient tumors (Figure 2H). Moreover, the *Olig2*cKO group exhibited an elevated cytotoxicity score in CD8^+^ T cells (Supplemental Figure 2D).

To further investigate the impact of *Olig2*-depleted GSCs on CD8^+^ T cell function, conditioned media (CM) from GSCs isolated from *Olig2*cKO and Ctrl-T GBM tissues was applied to CD8^+^ T cells to assess their activity. Treatment with *Olig2*cKO CM led to enhanced CD8^+^ T cell migration and proliferation, along with increased IFN-γ expression (Figure 2, I and J, and Supplemental Figure 2, E and F), indicating a heightened antitumor immune response in *Olig2*cKO tumors.

The scRNA-seq analysis of TAMs revealed upregulated expression of genes involved in antigen presentation in *Olig2*-depleted tumors. These genes were enriched in pathways associated with phagocytosis and immune effector processes in *Olig2*cKO tumors (Figure 2K). Conversely, scRNA-seq identified downregulated immunosuppressive genes, primarily related to cell–cell adhesion and myeloid migration pathways (Figure 2L).

To assess the effect of *Olig2*cKO CM on TAM activity in vitro, primary BMDMs and cell lines (Raw264.7 and BV2) were treated with *Olig2*cKO CM, followed by qPCR analysis. Treatment with *Olig2*cKO CM resulted in suppressed expression of antiinflammatory markers, while proinflammatory markers remained unaffected in macrophages (Figure 2M and Supplemental Figure 2, G and H). Consistently, the migration of treated macrophages was significantly reduced (Supplemental Figure 2I). Moreover, a similar effect was observed in macrophages that had been prepolarized with mIL-4 to differentiate into M2-like macrophages, where *Olig2*cKO CM suppressed antiinflammatory marker expression (Supplemental Figure 2J). Together, these findings indicate that OLIG2 deletion reshapes the immune microenvironment by enhancing CD8^+^ T cell infiltration and activation while suppressing the protumor activity of TAMs, thereby shifting the TME from an immunosuppressive protumor state to a more antitumor state.

### OLIG2-depleted GSCs enhance CD8^+^ T cell infiltration and activation through CXCL10 upregulation.

**Figure 3:**
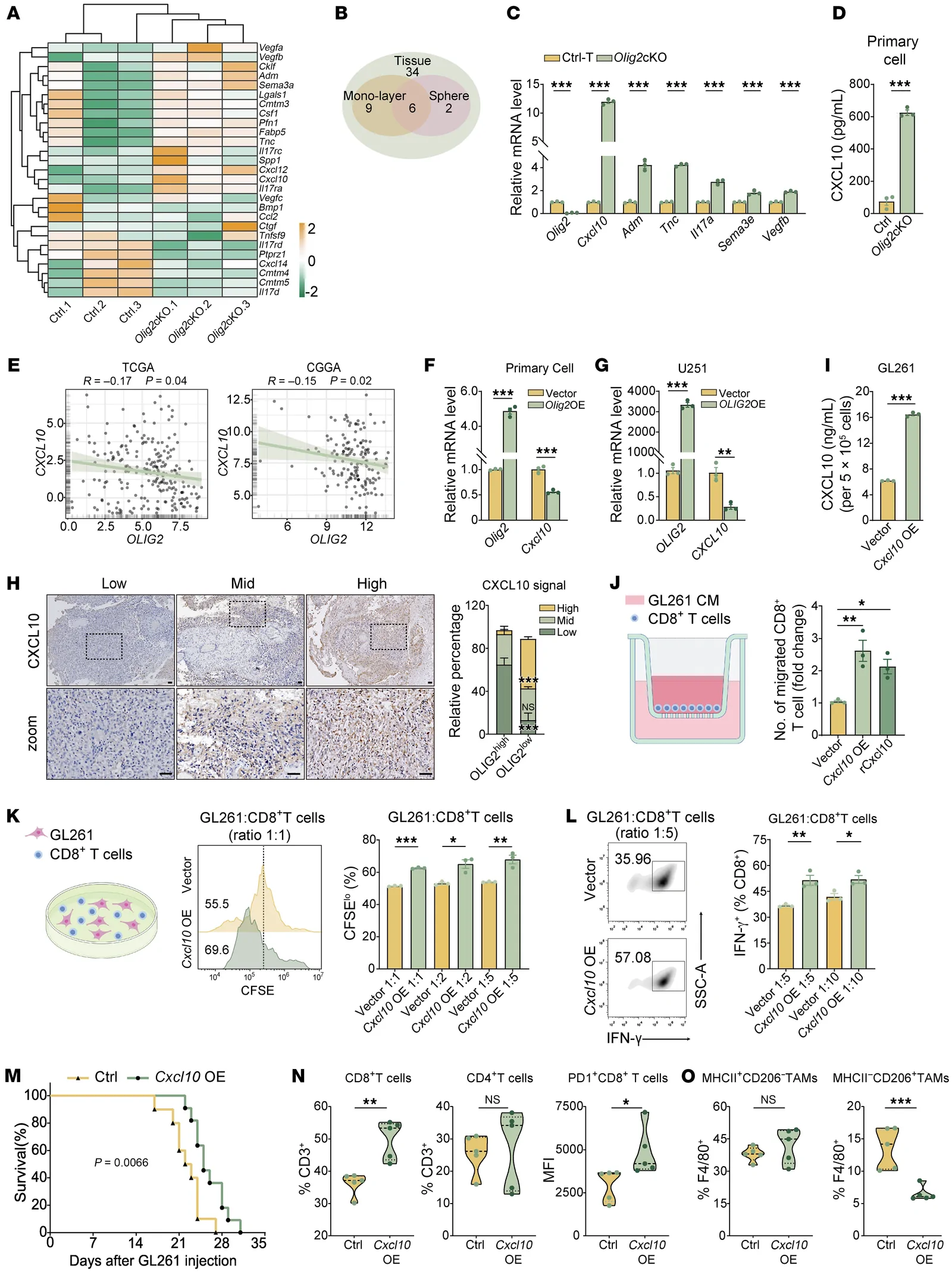
CXCL10 promotes antitumor immune response of CD8^+^ T cells. (**A**) Heatmap showing the expression of secreted proteins in the RNA-seq data of Ctrl-T and *Olig2*cKO GBM cells. (**B**) Venn diagram showing the shared secreted proteins among tumor tissue, spheres, and monolayers from Ctrl-T and *Olig2*cKO mice. (**C**) qPCR of mRNA expression of secreted proteins from Ctrl-T and *Olig2*cKO tumor cells (*n* = 3). (**D**) ELISA of CXCL10 secretion levels from the supernatant of Ctrl-T and *Olig2*cKO tumor cells (*n* = 3). (**E**) Correlation between *OLIG2* and *CXCL10* expression in TCGA GBM datasets and the Chinese Glioma Genome Atlas database. (**F** and **G**) qPCR of mRNA expression of *CXCL10* in primary mouse GBM cells and U251 cells upon *OLIG2* overexpression (OE) at 48 hours (*n* = 3). (**H**) Representative imaging and quantification of CXCL10 in GBM specimen with high or low OLIG2 expression (*n* = 19). Scale bars: 50 µm. (**I**) ELISA of CXCL10 secretion levels from the supernatant of *Cxcl10*-overexpressing GL261 cells (*n* = 3). (**J**) Quantification of migrated CD8^+^ T cells toward CM from the indicated treatment by transwell assay (*n* = 3). (**K**) Representative histograms and quantifications of CFSE proliferation assay of CD8^+^ T cells treated with CM from GL261-Ctrl and GL261-*Cxcl10* OE cells for 72 hours (*n* = 3). (**L**) Representative plots and quantification of the IFN-γ expression of CD8^+^ T cells cocultured with GL261-Ctrl and GL261-*Cxcl10* OE cells at the indicated ratio for 24 hours (*n* = 3). (**M**) Kaplan-Meier survival curves of orthotopic transplanted mice with Ctrl or *Cxcl10* OE GL261 cells (*n* = 10 mice/group). Log-rank analysis was used to assess significance. (**N** and **O**) Flow cytometry quantification of the indicated T cells and macrophages in GBMs from orthotopic transplanted mice with GL261-Ctrl and GL261-*Cxcl10* OE cells (*n* = 5/group). Statistical significance was determined by unpaired 2-tailed Student’s *t* test in **C**, **D**, **F**, **G**, **I**, **K**, **L**, **N**, and **O**; 2-way ANOVA in **H**; and 1-way ANOVA in **J**. *P < 0.05, **P < 0.01, ***P < 0.001.

Since *Olig2*cKO CM induced distinct phenotypic changes in T cells and macrophages, we next investigated which soluble proteins play key roles in this regulation. To identify candidate factors, we reanalyzed our RNA-seq data from *Olig2*cKO tumor cells and tissues, focusing on genes encoding secreted proteins. Six differentially expressed genes, predicted to be expressed in GSCs cultured as both monolayers and spheres, were selected for subsequent validation using qPCR (Figure 3, A and B). Among the identified candidates, *Cxcl10* was the most upregulated target upon *Olig2* deletion in GSCs (Figure 3C), which was further validated at the protein level by Western blot (Supplemental Figure 3A). The level of secreted CXCL10 was significantly higher in *Olig2*cKO GSCs compared with Ctrl-T GSCs (Figure 3D). While RNA-seq analysis revealed no significant differential expression of *Cxcl9* and *Cxcl11*, subsequent qPCR validation demonstrated modest upregulation of these chemokines (Supplemental Figure 3B). Crucially, *Cxcl10* exhibited the highest expression levels among these chemokines, suggesting that it was the dominant contributor to TME remodeling. Notably, *OLIG2* expression was negatively correlated with *CXCL10* levels across multiple databases (Figure 3E and Supplemental Figure 3C). Furthermore, overexpression of *Olig2* suppressed *Cxcl10* mRNA expression in Ctrl-T GSCs, U251 cells, and IFN-γ–stimulated GL261 cells (Figure 3, F and G, and Supplemental Figure 3E). Analysis of GBM tumor specimens further confirmed a higher proportion of strong CXCL10 signals in OLIG2^lo^ GBM patients (Figure 3H). Additionally, this CXCL10^hi^ tumor cell population was identified by IF in *Olig2*cKO mice carrying the Rosa26-tdTomato reporter (Supplemental Figure 3D).

Next, we investigated whether CXCL10 elevation enhances the antitumor CD8^+^ T cell response in *Olig2*cKO GBMs. To assess this, supernatants from *Cxcl10*-overexpressing GL261 cells were applied to CD8^+^ T cells, followed by a migration assay. The transwell assay demonstrated that these supernatants significantly promoted CD8^+^ T cell migration, a result that was further corroborated by treatment with recombinant CXCL10 protein (Figure 3, I and J, and Supplemental Figure 3E). Supernatants from *Cxcl10*-overexpressing cells also enhanced CD8^+^ T cell proliferation and IFN-γ production (Figure 3, K and L, and Supplemental Figure 3, F and G). To assess the impact of CXCL10 on T cell–mediated cytotoxicity, we performed a tumor cell killing assay by coincubating *Cxcl10*-overexpressing GL261 cells with CD8^+^ T cells. The proportion of propidium iodide–positive (PI^+^) tumor cells was significantly increased in the *Cxcl10*-overexpressing group compared with controls (Supplemental Figure 3H), suggesting that overexpression of *Cxcl10* enhances CD8^+^ T cell–mediated tumor cell killing. Furthermore, in an orthotopic syngeneic murine GBM model, transplantation of *Cxcl10*-overexpressing GL261 cells resulted in significantly prolonged survival (Figure 3M). Flow cytometry analysis of tumor tissues revealed increased CD8^+^ T cell infiltration and a higher proportion of PD1^+^CD8^+^ T cells in the *Cxcl10*-overexpressing group compared with the control (Figure 3N). These observations suggest that OLIG2 depletion in GSCs leads to upregulated CXCL10 production, which in turn enhances CD8^+^ T cell activation.

### CXCL10 regulates immunogenic TAMs in a STAT3-dependent manner.

**Figure 4:**
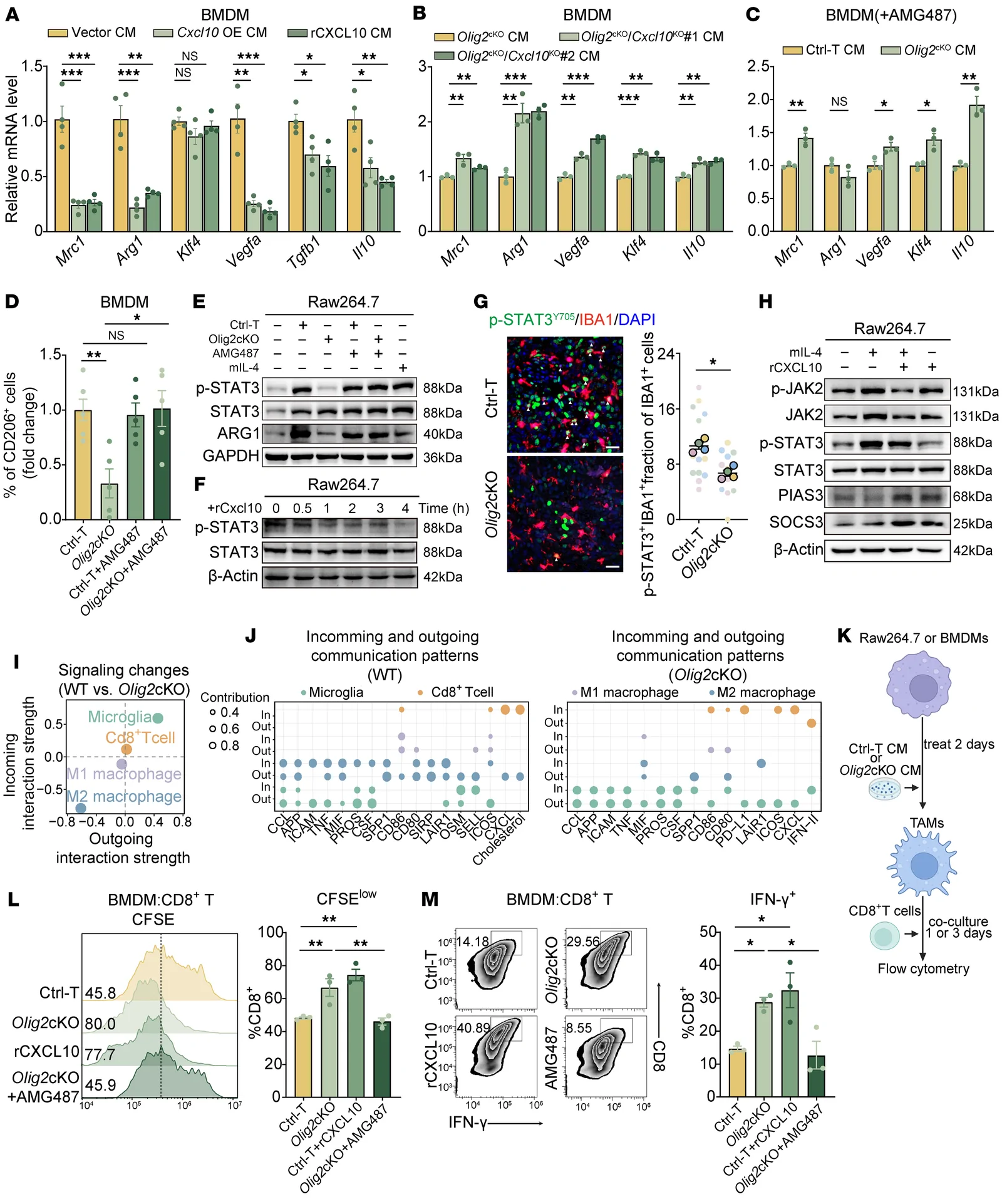
CXCL10 reduces M2-like polarization of macrophages by inhibiting STAT3 signaling. (**A**) qPCR of mRNA expression of antiinflammatory markers in BMDMs treated with CM from the indicated groups (*n* = 4). (**B**) qPCR of mRNA expression of antiinflammatory markers in BMDMs treated with CM from the indicated groups (*n* = 3). (**C**) qPCR of mRNA expression of antiinflammatory markers in BMDMs treated with Ctrl-T or *Olig2*cKO CM containing 1 μM AMG487 (*n* = 3). (**D**) Quantification of CD206 expression in BMDMs treated with Ctrl-T and *Olig2*cKO CM with or without AMG487 for 48 hours (*n* = 5). (**E**) Western blot showing the protein levels in Raw264.7 cells cocultured with CM from the indicated treatment. (**F**) Western blot showing the protein levels in Raw264.7 cells at the indicated time points. (**G**) Representative images and quantification of p-STAT3 and IBA1 in Ctrl-T and *Olig2*cKO tumor tissues (*n* = 4/group). Scale bars: 20 µm. (**H**) Western blot showing the protein levels in mIL-4– and CXCL10-treated Raw264.7 cells. (**I**) Scatterplot depicting the interaction strength of individual cell subsets across all identified signaling pathways. (**J**) Dot plot illustrating the incoming and outgoing communication patterns of individual signaling pathways across distinct cell subsets. Dot size represents the relative contribution of the corresponding pathway to the total detected communication patterns within each cell subset. (**K**) Schematic of the coculture assays of CD8^+^ T cells treated with CM-incubated Raw264.7 or BMDMs. (**L**) Representative histograms and quantification of CFSE proliferation assay of CD8^+^ T cells cocultured with BMDM cells preincubated with CM from the indicated treatment for 72 hours (*n* = 3). (**M**) Representative plots and quantification of the IFN-γ expression of CD8^+^ T cells cocultured with BMDMs preincubated with CM from the indicated treatment for 24 hours (*n* = 3). Statistical significance was determined by 1-way ANOVA in **D**, **L**, and **M** and unpaired 2-tailed Student’s *t* test in **A**–**C** and **G**. *P < 0.05, **P < 0.01, ***P < 0.001.

We next examined whether CXCL10 was responsible for the suppression of TAM activity in *Olig2*cKO tumors. Treatment of both murine and human macrophages with *CXCL10*-overexpressing CM or recombinant CXCL10 protein led to a significant decrease in the mRNA levels of antiinflammatory markers (Figure 4A and Supplemental Figure 4, A–C). Notably, the expression of these markers was restored in macrophages cocultured with *Olig2*cKO CM in which *Cxcl10* had been knocked out (Figure 4B and Supplemental Figure 4, D–F). In addition, we treated BMDMs or Raw264.7 cells with *Olig2*-deficient CM in the presence of AMG487, a selective antagonist of CXCR3 (CXCL10’s receptor), and analyzed antiinflammatory marker expression. Our results showed that AMG487 reversed the suppression of alternative macrophage activation (Figure 4, C and D, and Supplemental Figure 4, G–I). Moreover, flow cytometry analysis of tumor tissues from the *Cxcl10*-overexpressing GL261 syngeneic mouse model revealed a significant reduction in antiinflammatory TAMs (Figure 3O). These findings suggest a critical role of CXCL10/CXCR3 signaling in driving TAM reprogramming toward a proinflammatory phenotype.

To further investigate the mechanism by which CXCL10 mediates TAM reprogramming, we examined key signaling pathways involved in TAM alternative activation, including ERK, AKT, STAT1, and STAT3 (29–32). An obvious decrease in phosphorylated STAT3 (p-STAT3) levels was observed in Raw264.7 cells treated with *Olig2*cKO CM, which was reversed upon AMG487 treatment (Figure 4E and Supplemental Figure 4J). A time-course experiment using recombinant CXCL10 demonstrated a progressive decline in STAT3 phosphorylation in Raw264.7 and THP-1 cells over time (Figure 4F and Supplemental Figure 4K). Additionally, immunofluorescence (IF) staining confirmed a reduced population of p-STAT3^+^ TAMs in *Olig2*cKO GBM tissues (Figure 4G). We further evaluated the inhibitors of STAT3 phosphorylation, including SOCS1, SOCS3, PIAS1, and PIAS3 (33, 34), and CXCL10 treatment upregulated the expression of SOCS3 and PIAS3 in mIL-4–polarized TAMs (Figure 4H and Supplemental Figure 4, L and M). The same treatment reversed the mIL-4–induced elevation of p-STAT3 and p-JAK2 levels in Raw264.7 cells (Figure 4H). AMG487 treatment also restored the elevation of p-STAT3 and p-JAK2 levels that were suppressed by *Olig2*cKO CM in Raw264.7 cells (Supplemental Figure 4M). These findings indicate that SOCS3 and PIAS3 play critical roles in CXCL10-dependent inhibition of STAT3 signaling.

### CXCL10 orchestrates TAMs to enhance the antitumor immune response of T cells.

Given that CXCL10 regulates both CD8^+^ T cells and TAMs, we explored this interaction by performing interactome analysis of TAMs and CD8^+^ T cells using our scRNA-seq data to determine whether *Olig2* mediates TAM-driven suppression of CD8^+^ T cells. Based on TAM subpopulations classified into M1-like, M2-like, and microglia, we found that M2-like macrophages exhibited remarkably diminished interactions with other cell populations in the *Olig2*cKO group (Figure 4I). Further characterization of intercellular communication between macrophages and the TME revealed that, in *Olig2*cKO tumors, immunosuppressive signals, including SPP1, OSM, MIF, and SELL, as well as migration cues such as CCL and CXCL, showed a marked reduction in functional intensity. Conversely, immune activation signals, particularly CD86 and CD80, were markedly upregulated, indicating a transition toward a more immunostimulatory TME. At the same time, CD8^+^ T cells in the *Olig2*cKO group displayed an enriched array of activation cues, including enhanced costimulatory signaling and elevated IFN signaling, reinforcing a heightened antitumor immune response. Intriguingly, we also observed a marked augmentation in the immune checkpoint signal PD-L1 (Figure 4J). These findings aligned with our previous analysis, which indicated that both activation and exhaustion of CD8^+^ T cells are concurrently elevated in the *Olig2*cKO group (Figure 2G).

To further investigate whether upregulated CXCL10 reprograms TAMs to enhance CD8^+^ T cell–mediated antitumor responses in *Olig2*-deficient tumors, we employed a coculture system. In this assay, Raw264.7 cells or BMDMs were treated with Ctrl-T or *Olig2*cKO CM, followed by coculture with CD8^+^ T cells (Figure 4K). We observed increased CD8^+^ T cell proliferation and elevated IFN-γ production in the *Olig2*cKO CM-treated group (Supplemental Figure 4, N and O).

Next, the coculture system was supplemented with either recombinant CXCL10 or AMG487 (a CXCR3 antagonist), followed by an assessment of CD8^+^ T cell activity. The results showed that recombinant CXCL10 enhanced CD8^+^ T cell activation in the Ctrl-T CM-treated group. Conversely, the addition of AMG487 significantly reduced CD8^+^ T cell activation in the *Olig2*cKO CM-treated group, bringing it to levels similar to those observed in the Ctrl-T CM-treated group (Figure 4, L and M). Together, these findings demonstrate that OLIG2 deficiency induces CXCL10 upregulation, which inhibits STAT3 phosphorylation, thereby preventing TAM polarization toward an immunosuppressive phenotype. This shift may foster an antitumor microenvironment and enhance CD8^+^ T cell–mediated antitumor immunity.

### OLIG2 recruits HDAC7 to suppress CXCL10 transcription.

**Figure 5:**
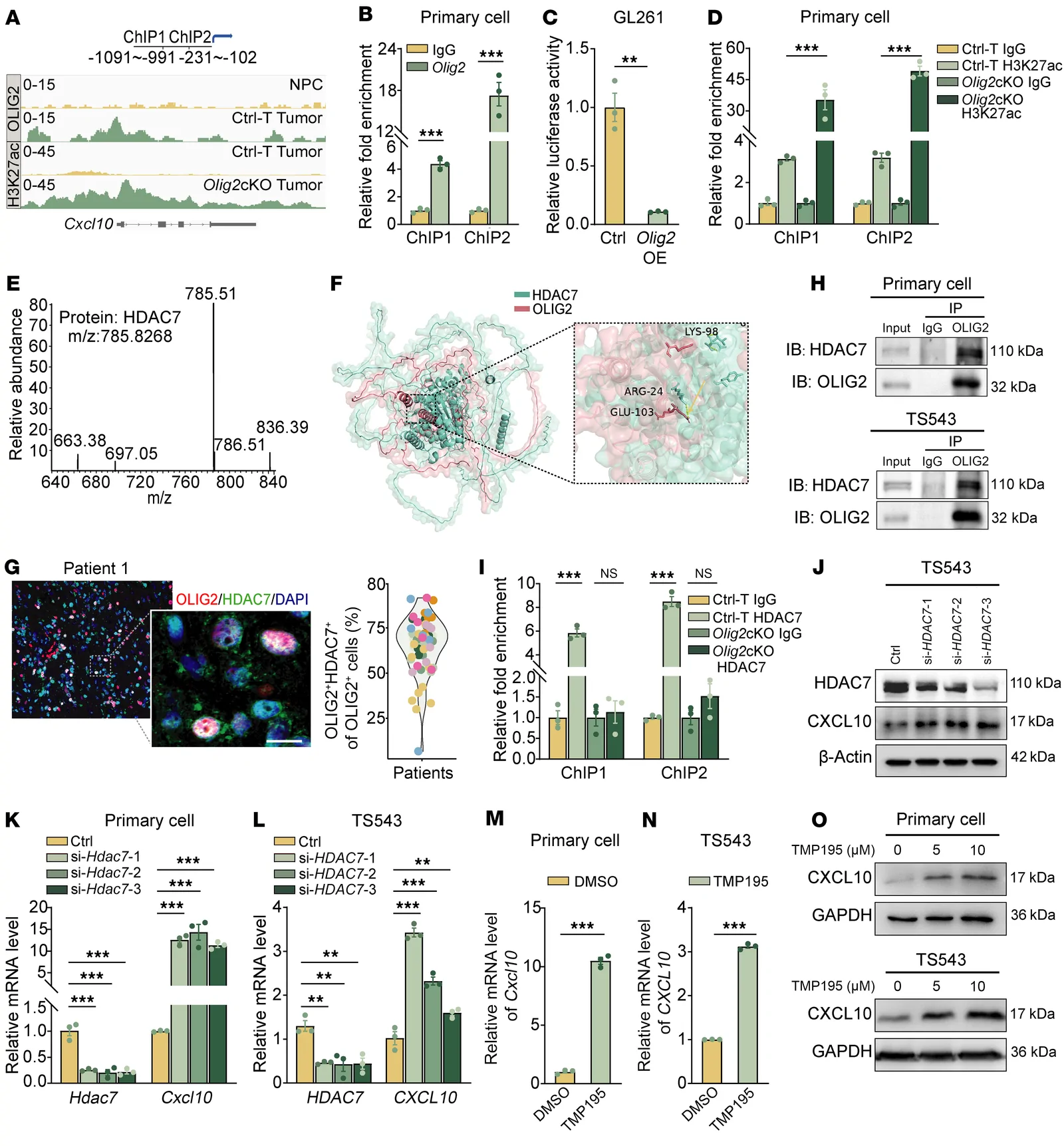
OLIG2 recruits HDAC7 to inhibit *CXCL10* expression in GBM cells. (**A**) IGV browser visualization of OLIG2 or H3K27ac binding regions on the gene loci of *Cxcl10* in NPCs, Ctrl-T, and *Olig2*cKO tumors. (**B**) ChIP-qPCR of OLIG2 enrichment in enhancer regions of *Cxcl10* in Ctrl-T cells (*n* = 3). (**C**) Dual-luciferase reporter assay of *Cxcl10* enhancer activity in control or *Olig2* overexpressed GL261 cells (*n* = 3). (**D**) ChIP-qPCR of H3K27ac enrichment in enhancer regions of *Cxcl10* in Ctrl-T and *Olig2*cKO cells (*n* = 3). (**E**) HDAC7 peptide identified by mass spectrometry. (**F**) Molecular docking assay predicting the binding sites of OLIG2 and HDAC7 proteins. The docking interfaces are highlighted by dashed lines (yellow arrow). (**G**) Representative images and quantification of OLIG2 and HDAC7 staining of human GBM specimen (*n* = 10 patients). Scale bar: 10 μm. (**H**) Co-IP assay to detect the binding between OLIG2 and HDAC7. (**I**) ChIP-qPCR of HDAC7 enrichment in enhancer regions of *Cxcl10* in Ctrl-T and *Olig2*cKO cells (*n* = 3). (**J**) Western blot showing protein levels of HDAC7 and CXCL10 in Ctrl and si*HDAC7* TS543 cells. (**K**) qPCR of mRNA expression of *Cxcl10* in Ctrl-T tumor cells transfected with si*HDAC7* (*n* = 3). (**L**) qPCR of mRNA expression of *CXCL10* in Ctrl and si*HDAC7* TS543 cells (*n* = 3). (**M** and **N**) qPCR of mRNA expression of *Cxcl10* in Ctrl-T tumor cells and TS543 cells treated with 10 μM TMP195 for 48 hours (*n* = 3). (**O**) Western blot showing protein levels of CXCL10 in Ctrl-T tumor cells and TS543 cells treated with TMP195 for 48 hours. Statistical significance was determined by unpaired 2-tailed Student’s *t* test in **B**, **C**, **M**, and **N** and 1-way ANOVA in **D**, **I**, **K**, and **L**. **P < 0.01, ***P < 0.001.

To investigate the potential mechanisms by which CXCL10 is transcriptionally regulated by OLIG2, we performed ChIP-seq analysis and identified prominent enrichment of OLIG2 binding at the enhancer regions of CXCL10 (Figure 5A). ChIP-qPCR further validated that OLIG2 binds to these regulatory regions (Figure 5B). Dual-luciferase reporter assays confirmed the repressive role of OLIG2 in *Cxcl10* transcription, as overexpression of *Olig2* significantly reduced the luciferase activity associated with *Cxcl10* enhancer elements in GL261 and 293T cells compared with vector controls (Figure 5C and Supplemental Figure 5A). Additionally, ChIP-seq analysis of Ctrl-T tumors revealed decreased levels of the enhancer H3K27ac in the *Cxcl10* transcriptional regulatory region (Figure 5A). Conversely, ChIP-qPCR analysis of *Olig2*cKO cells showed increased H3K27ac levels at the *Cxcl10* enhancer region (Figure 5D). These findings suggest that *Olig2* directly targets and suppresses *Cxcl10* expression in GSCs.

To identify key components interacting with OLIG2, we performed immunoprecipitation–mass spectrometry and molecular docking analysis, which revealed HDAC7 as a potent binding partner of OLIG2, playing a critical role in regulating CXCL10 expression (Figure 5, E and F). IF staining confirmed the colocalization of OLIG2 and HDAC7 in GBM tissues (Figure 5G and Supplemental Figure 5B). The interaction between endogenous HDAC7 and OLIG2 was further validated by coimmunoprecipitation (co-IP) in both murine primary GSCs and TS543 cells (Figure 5H). Consistently, HDAC7 occupancy at the *Cxcl10* enhancer region was markedly reduced in *Olig2*cKO GSCs (Figure 5I). Pharmacological inhibition of HDAC7 using the HDAC4/7/9 inhibitor TMP195 or HDAC7-specific siRNAs led to a significant increase in *CXCL10* mRNA and protein levels, further supporting the role of HDAC7 in the transcriptional repression of *CXCL10* (Figure 5, J–O, and Supplemental Figure 5C). Collectively, these findings demonstrate that the OLIG2/HDAC7 complex suppresses *CXCL10* transcription in GSCs, thereby modulating immune responses within the GBM microenvironment.

### OLIG2 deletion enhances the efficacy of anti–PD-L1 therapy for GBMs.

**Figure 6:**
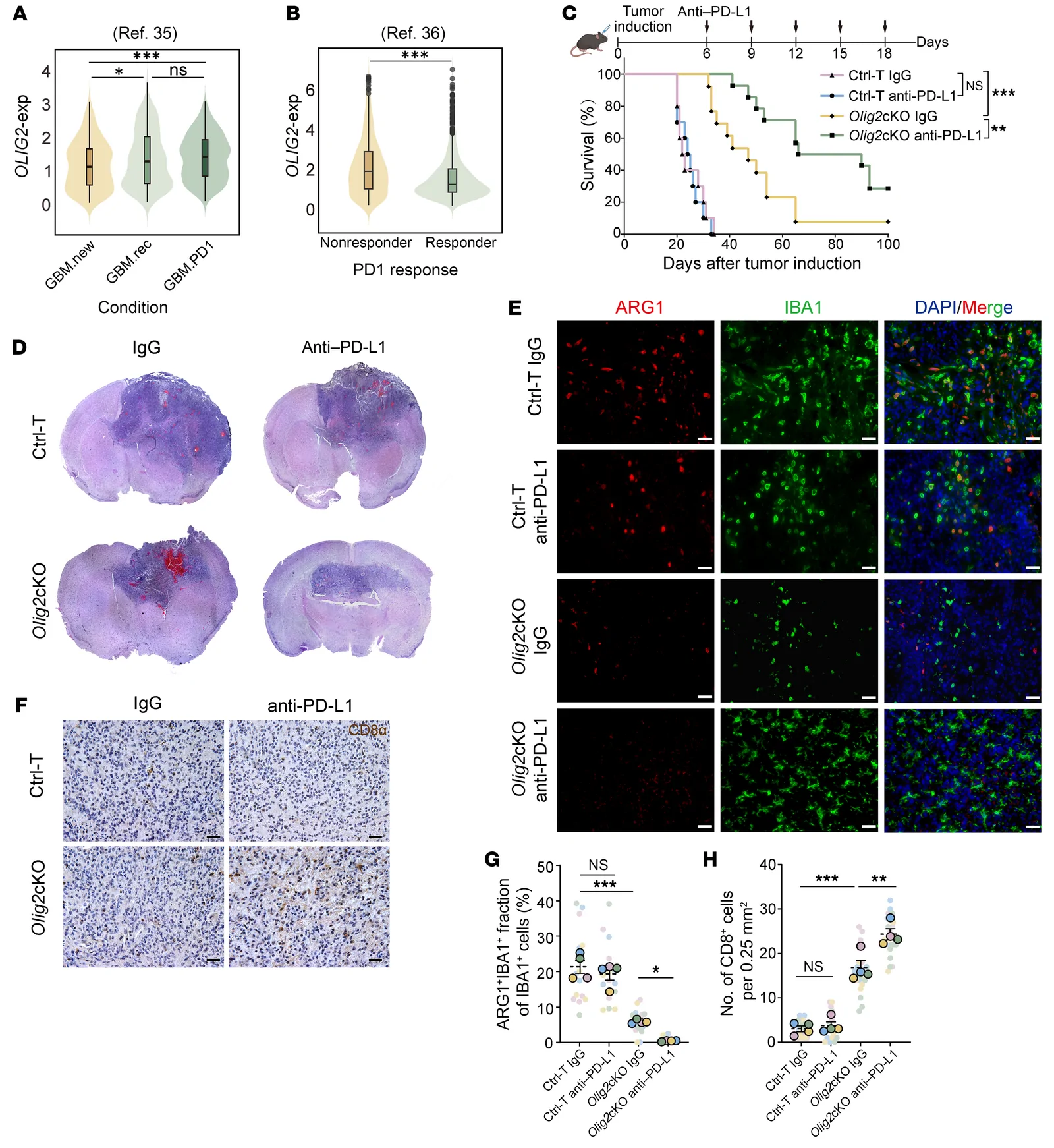
OLIG2 deficiency strengthens the effects of anti–PD-L1 therapy in GBMs. (**A**) The relative expression of *OLIG2* in the scRNA-seq dataset of treatment-naive, recurrent, and neoadjuvant anti–PD-1–treated GBM patient samples (Gene Expression Omnibus, GSE154795; ref. 35). (**B**) The relative expression of *OLIG2* in the scRNA-seq dataset of response and nonresponse groups to anti–PD-1 therapy in patients with GBMs (National Genomics Data Center, HRA004677; ref. 36). (**C**) Schematic and Kaplan-Meier survival curve of Ctrl-T or *Olig2*cKO mice treated with anti–PD-L1 (10 mg/kg) antibody. Log-rank analysis was used to assess significance (*n* = 10–14 mice/group). (**D**) H&E staining showing tumors from Ctrl-T or *Olig2*cKO mice treated with IgG and anti–PD-L1 antibody. (**E** and **G**) Representative images and quantification of ARG1, IBA1, and nuclei in tumor tissues from Ctrl-T or *Olig2*cKO mice treated with IgG or anti–PD-L1 antibody. Scale bars: 20 μm (*n* = 4/group). (**F** and **H**) Representative images and quantification of CD8α in Ctrl-T or *Olig2*cKO mice treated with IgG or anti–PD-L1 antibody (*n* = 4/group). Scale bars: 20 μm. Statistical significance was determined by 1-way ANOVA in **G** and **H**. *P < 0.05, **P < 0.01, ***P < 0.001.

Given the immunosuppressive properties of OLIG2^+^ GSCs, we investigated whether OLIG2 expression could predict the response to T cell–directed ICI therapy in patients with GBMs. Recent clinical trials evaluating neoadjuvant anti–PD-1 therapy in resectable recurrent GBMs have demonstrated notable survival benefits compared with adjuvant treatment alone (35). Analysis of scRNA-seq data from one such clinical trial revealed a significantly higher expression of OLIG2 in neoadjuvant anti–PD-1–treated tumors compared with treatment-naive samples (Figure 6A), suggesting that OLIG2^+^ GSCs may contribute to anti–PD-1 resistance. Furthermore, analysis of a separate scRNA-seq dataset from GBM patients treated with ICIs (36) showed that tumor cells from ICI-responsive patients exhibited lower OLIG2 expression (Figure 6B). These findings underscore the potential of OLIG2 as a therapeutic target to enhance the efficacy of ICI therapy and improve treatment outcomes in patients with GBMs.

Since our data indicate that *Olig2*cKO mice exhibit an enhanced CD8^+^ T cell response, we next investigated whether *Olig2* silencing could enhance the efficacy of anti–PD-L1 therapy, which has demonstrated limited effectiveness against GBM as a monotherapy. To assess this, Ctrl-T and *Olig2*-ablated GBM mice were treated with an anti–PD-L1 antibody, followed by evaluation of tumor progression. While anti–PD-L1 treatment had minimal impact on the survival of Ctrl-T mice, *Olig2*cKO mice receiving PD-L1 blockade demonstrated a significant improvement in median survival and a marked reduction in tumor size (Figure 6, C and D). Further analysis of TME remodeling using IF and IHC staining revealed that PD-L1 blockade significantly enhanced CD8^+^ T cell infiltration, while reducing the number of ARG1^+^ TAMs in *Olig2*cKO mice (Figure 6, E–H). Collectively, these in vivo findings suggest that the combination of OLIG2 deficiency and anti–PD-L1 therapy substantially augments both innate and adaptive antitumor immune responses against GBMs.

### CT-179 potentiates the efficacy of anti–PD-L1 therapy.

A selective OLIG2 inhibitor, CT-179, was previously shown to suppress tumor growth in medulloblastoma models (37, 38). To assess its therapeutic potential in GBMs, we determined the IC_50_ of CT-179 in primary mouse GBM cells and in the human GBM line TS543 (Supplemental Figure 6, A and B). Consistent with its role in inhibiting OLIG2 transcriptional activity, qPCR analysis revealed that CT-179 treatment significantly downregulated OLIG2 target genes, including *AURKA*, *CDC20*, and *CDCA8*, while *CXCL10* expression was markedly upregulated (Supplemental Figure 6, C–F). Notably, CT-179 did not substantially alter OLIG2 protein abundance.

**Figure 7:**
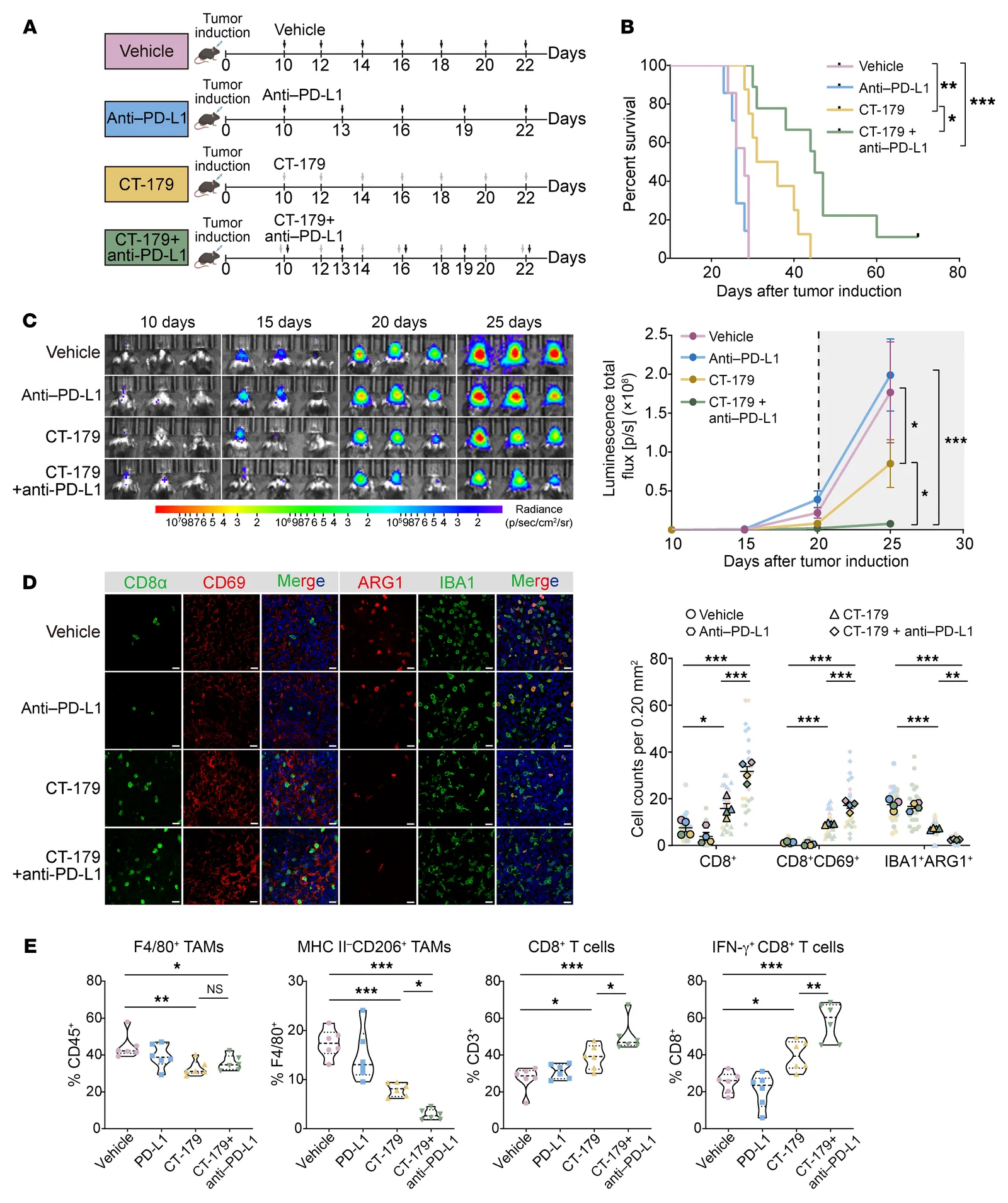
CT-179 potentiates anti–PD-L1 therapy efficacy. (**A**) Treatment strategy for evaluating CT-179 and anti–PD-L1 therapy in a murine GBM model. Black and gray arrows indicate time points of anti–PD-L1 antibody injection and CT-179 administration, respectively. (**B**) Kaplan-Meier survival analysis of mice treated with monotherapy or combinational therapy (*n* = 8–10 mice/group). Vehicle control (pink), anti–PD-L1 monotherapy (10 mg/kg, blue), CT-179 monotherapy (20 mg/kg, yellow), and combination therapy (green). Log-rank analysis was used to assess significance. (**C**) Representative in vivo bioluminescence images and quantification of 4 treatment groups at indicated time points (*n* = 5–6 mice/group). (**D**) Representative IF images and quantification of tumor sections from 4 treatment groups (*n* = 4 mice/group). Scale bars = 20 μm. (**E**) Flow cytometry quantification of the F4/80^+^ TAMs, MHC II^–^CD206^+^ TAMs, CD8^+^ T cells, and IFN-γ^+^ CD8^+^ T cells in tumor tissues (*n* = 6 mice/group). Data represent mean ± SEM. Statistical significance was determined by 1-way (**E** and **D**) or 2-way ANOVA (**C**) with Tukey’s post hoc test. *P < 0.05, **P < 0.01, ***P < 0.001.

We next assessed CT-179 efficacy in vivo. CT-179 monotherapy reduced tumor growth and extended survival in GBM-bearing mice. Importantly, combining CT-179 with PD-L1 blockade further diminished tumor burden and yielded a significantly greater survival benefit compared with either treatment alone (Figure 7, A–C).

To assess how CT-179 influences the TME, we performed immune profiling and observed changes that mirrored those in the *Olig2*cKO model. Notably, the combination treatment significantly increased the proportion of CD8^+^ T cells and reduced immunosuppressive IBA1^+^ARG1^+^ TAM subpopulations (Figure 7D). Flow cytometry further confirmed that CT-179 combined with PD-L1 blockade decreased MHCII^–^CD206^+^ TAMs while increasing both the infiltration of CD8^+^ T cells and the proportion of IFN-γ^+^CD8^+^ effector T cells (Figure 7E). In addition, reduced ARG1 expression following OLIG2 inhibition was primarily detected in IBA1^+^ and CD68^+^ TAM subsets (Supplemental Figure 6, G–I). Collectively, these findings indicate that CT-179 not only exerts direct antitumor effects but also remodels the tumor immune microenvironment to potentiate antitumor immune responses.

## Discussion

GBMs are widely recognized as immunologically “cold” tumors due to their limited immune cell infiltration and profoundly immunosuppressive microenvironment (39). A major challenge in improving ICI therapies for GBMs is understanding the mechanisms that exclude cytotoxic T cells from the TME. In this study, we identify a tumor-intrinsic mechanism regulated by OLIG2, which modulates CD8^+^ T cell and TAM activity through an HDAC7/CXCL10-dependent signaling axis. Notably, OLIG2 blockade markedly potentiated the efficacy of anti–PD-L1 therapy, highlighting OLIG2 as a promising therapeutic target in combination with ICIs for GBM treatment.

Our previous work demonstrated that OLIG2 promotes the tumor-propagating capacity of GSCs (11). Here, we reveal that OLIG2^+^ GSCs suppress CXCL10, contributing to an immunosuppressive TME by impairing T cell–mediated antitumor immunity. These findings were primarily characterized in a murine model of proneural GBMs, and we extended our analyses to U251 (classical-like GBM) and GL261 (mesenchymal-like GBM) cells to account for intratumoral heterogeneity in GSC-driven ICI resistance. Bioinformatic analysis revealed a marked inverse correlation between *OLIG2* and *CXCL10* expression in both proneural and mesenchymal GBM subtypes (Supplemental Figure 3I). These findings suggest that OLIG2-mediated repression of CXCL10 is a prominent feature across these GBM subtypes, underscoring OLIG2 as a key regulator of the GBM immune landscape. Additional factors probably regulate CXCL10 in nonproneural GBM subtypes, with relatively low OLIG2 expression. Further studies are required to elucidate these mechanisms and optimize subtype-specific therapeutic strategies.

As the dominant immune cell population in GBMs, TAMs facilitate tumor progression by hijacking proinflammatory pathways to sustain immunosuppression (7, 40, 41). Our findings reveal that CXCL10 inhibits TAM polarization toward a protumor phenotype, consistent with previous studies demonstrating that CXCL10 enhances TAM recruitment, proliferation, and antitumor activity (42, 43). Mechanistically, we show that CXCL10 suppresses TAM polarization via STAT3 inhibition, mediated by SOCS3 and PIAS3 downstream of the CXCL10/CXCR3 axis.

Furthermore, OLIG2 ablation reduces TAM infiltration in murine GBMs, raising the possibility that additional secreted factors may contribute to OLIG2-driven TAM chemotaxis, which warrants further investigation.

We found that *Olig2* deletion resulted in marked upregulation of chemokines such as *Cxcl9*-*11*, which are known to promote T cell recruitment (44), alongside downregulation of immunosuppressive genes including *Ptprz1*, *Cmtm4*, and *Cmtm5*. Modest increases were also observed in transcripts linked to angiogenesis and extracellular matrix remodeling (e.g., *Vegf*, *Tnc*, *Fabp5*, and *Spp1*). Despite these potentially protumorigenic changes, OLIG2 loss consistently delayed tumor progression and significantly prolonged survival in vivo. These data indicate that the immunostimulatory consequences of OLIG2 deletion, namely, enhanced T cell infiltration and reduced immunosuppressive signaling, override any counteracting effects associated with angiogenesis- or ECM-related gene upregulation.

Chemokine regulation within the glioma microenvironment is shaped by multiple signaling pathways. Mondal et al. recently reported that glioma-specific PP2A deletion activates the cGAS-STING pathway, leading to type I interferon induction and robust CXCL10 upregulation (45). By contrast, our findings revealed that OLIG2 forms a complex with HDAC7, which directly associates with the CXCL10 enhancer to repress its transcription. Importantly, we did not observe activation of cGAS-STING or IFN-I signaling signatures in OLIG2-deficient tumors, suggesting that OLIG2-mediated repression of CXCL10 occurs independently of the PP2A/cGAS-STING signaling. Together, these results support a model in which CXCL10 expression is subject to distinct, yet potentially convergent, regulatory pathways, with cGAS-STING operating upstream of or parallel to an OLIG2-HDAC7–mediated chromatin repression mechanism.

Given its central roles in tumor initiation, progression, and immune evasion, OLIG2 represents a compelling therapeutic target in GBMs. Our findings indicate that OLIG2 expression may serve as a predictive biomarker for responsiveness to ICI therapy, particularly in proneural and mesenchymal GBM subtypes. Consistent with the effects observed following genetic loss of OLIG2 in combination with anti–PD-1/PD-L1 blockade, pharmacological inhibition of OLIG2 using the small-molecule inhibitor CT-179 enhances antitumor immunity and further restrains tumor growth. These results highlight the potential of integrating OLIG2 inhibition with immunotherapy to overcome immune resistance in GBMs. Overall, our study delineates a mechanistic basis by which OLIG2^+^ GSCs orchestrate immunosuppressive signaling within the TME and positions OLIG2 as a promising target for next-generation combinatorial immunotherapies.

## Methods

### Sex as a biological variable.

Our study examined male and female animals, and similar findings are reported for both sexes.

### Cell cultures.

HEK293T (ATCC) and GL261 cells (from Yu Shi, Third Military Medical University, Chongqing, China) were cultured in DMEM (Gibco, 11965092) supplemented with 10% FBS (Vazyme, F101-01) and 1% penicillin-streptomycin (BasalMedia, S110JV). Human glioma TS543 cells and primary mouse glioma spheres were isolated from Ctrl-T and *Olig2*cKO tumor-bearing mice and maintained in DMEM/F-12 (Gibco, C11440500BT) with B-27(-A) (Gibco, 12587010), 20 ng/mL human bFGF (Novoprotein, C779), 20 ng/mL human EGF (Thermo Fisher Scientific, 13256-029), and 2 ng/mL heparin (Sigma-Aldrich, A8036). The Raw264.7, THP-1, U937 (from ATCC), and BV2 (from Chao Wang, Chongqing Medical University) cells were grown in RPMI 1640 (Sigma-Aldrich, R8758) with 10% FBS (Sigma-Aldrich, F8313) and 1% penicillin-streptomycin. All cells were cultured at 37°C in a humidified incubator containing 5% CO_2_. All the cells were authenticated by examination of morphology and growth characteristics and confirmed to be free of mycoplasma.

### Mice intracranial tumorigenesis and treatment.

C57BL/6 background *Pten*^fl/fl^ *Trp53*^fl/fl^ *Olig2*^fl/fl^ transgenic mice were obtained in-house. 4- to 8-week-old C57BL/6 mice of both sexes were purchased from Enswell Company. Animals were housed in a climate-controlled, pathogen-free facility with access to food and water ad libitum under a 12-hour light/dark cycle. GBMs were induced by PDGFB-IRES-Cre retroviral vector injections in the *Pten*^fl/fl^ *Trp53*^fl/fl^ transgenic mice. For white matter targeting, we used the coordinates of 0.5 mm rostral, 0.8 mm lateral, and 2.5 mm deep with respect to bregma. 2 μL retrovirus was injected into the brain using a brain stereotactic instrument (RWD Life Science). For intracranial transplantation, 5 × 10^4^ GL261 cells were injected intracranially into the striatum at a depth of 3.0 mm in C57BL/6 mice. The isotype IgG, anti–PD-L1, and anti-CD8 treatment was initiated on the sixth day after tumor induction by i.p. injection every 3 days for a total of 5 doses. CT-179 was administered via i.p. injection, initiating on day 10 after tumor induction. The treatment regimen consisted of 1 dose every other day for a total of 7 doses. In each experiment, mice were randomly assigned to 1 of the following arms: isotype IgG (10 mg/kg, Selleck, A2116); anti–PD-L1 (10 mg/kg, Selleck, A2115); anti-CD8α (10 mg/kg, Selleck, A2102); CT-179 (20 mg/kg, TargetMoI, 1996636-69-1). Tumor-bearing mice were monitored daily and euthanized when they reached the humane endpoint criteria.

### Isolation of single cells from mouse brain tumors.

The brain tumors were isolated, minced into small pieces, and digested by Accutase cell detachment solution (BioLegend, 423201) and DNase I at 37°C for 30 minutes. The digestion was terminated and filtered through a 70 μm cell strainer. Lipid was removed by 30% Percoll solution. Red blood cells were solubilized with red cell lysis buffer. The single cells were washed with PBS and then used for flow cytometry analysis or sorting.

### Cell viability assays.

Primary GBM cells derived from mouse tumors and TS543 cells were plated in 96-well plates at a density of 2,000 cells per well. Following seeding, the cells were treated with various concentrations of CT-179 (ranging from 0 to 16 μM; specifically, 0, 0.125, 0.5, 1, 2, 4, 8, and 16 μM for TS543 cells, and 0, 0.5, 1, 2, 3, 4, 8, and 16 μM for primary cells). After 48 hours of treatment, cell viability was determined using a CellTiter-Lumi Luminescent Cell Viability Assay kit (Beyotime, C0065M) according to the manufacturer’s protocol. Chemiluminescence was measured by a multifunctional microplate reader (Feyond-A300, ALLSHENG). The IC_50_ value was calculated by fitting dose–response data to a 4-parameter logistic model using GraphPad Prism software (version 10, GraphPad Software).

### Flow cytometry analysis.

Single-cell suspensions were washed once, blocked using anti-CD32/CD16 (BioLegend, 156604) to avoid nonspecific binding for 20 minutes at room temperature, and stained with cell surface antibodies for 25 minutes at room temperature. Intracellular staining (IFN-γ) was performed using a Cyto-Fast Fix/Perm Buffer Set (BioLegend, 426803), which is designed for fixation and permeabilization of mammalian cells for intracellular staining such as cytokines and other cytoplasmic molecules. Stained cells were acquired by flow cytometry (Beckman Coulter CytoFLEX), and the data were analyzed by FlowJo (version 10.8.1, BD Biosciences) or CytExpert (version 2.4, Beckman Coulter) software. Antibody information is listed in Supplemental Table 2.

### Mouse CD8^+^ T cell isolation.

For T cell isolation, spleens from C57BL/6 mice were isolated and passed through 70 μm cell strainer. After red blood cell lysis, CD8^+^ T cells were purified by CD8 microbeads (BioLegend, 480007) according to the manufacturer’s protocol. CD8^+^ T cells were stimulated with 3 μg/mL anti-CD3 and 1 μg/mL anti-CD28 antibodies with 20 ng/mL mIL-2 for 48 hours.

### T cell assay.

For in vitro migration assay, 5 × 10^5^ CD8^+^ T cells were put into the top well of a transwell chamber (3.0 μm). The bottom well was loaded with RPMI 1640 medium with glioma cell-CM or CM with added recombinant murine CXCL10. Plates were incubated at 37°C for 20 hours, and the cells of the bottom well were photographed and counted under a microscope in 4–6 randomly chosen fields. For the in vitro T cell proliferation assay, CD8^+^ T cells were labeled with 2.5 μM CFSE (MedChemExpress, HY-D0938) at 37°C for 10 minutes. For CellTrace Violet staining (Thermo Fisher Scientific, C34557), refer to the manufacturer’s instructions.

The primary GSCs or treated macrophages, as described above for CM, were cocultured with labeled CD8^+^ T cells at the indicated ratios, and the plates were incubated at 37°C for 72 hours. The cells were harvested and measured by flow cytometry. For in vitro T cell functional assay, the above cocultured system was incubated at 37°C for 24 hours. Before being harvested and assessed by flow cytometry to detect IFN-γ production, the cocultured CD8^+^ T cells were stimulated with 50 ng/mL PMA (MedChemExpress, HY-18739) and 1 μM ionomycin for 4 hours in the presence of Monensin (MedChemExpress, HY-N0150).

### T cell killing assay.

GL261 cells from the vector control group and the *Cxcl10-*overexpressing group were prelabeled with CFSE and cocultured with CD8^+^ T cells at ratios of 1:5 and 1:10 for 72 hours. After coculture, cell death was assessed using PI (Vazyme, A211-01) staining according to the manufacturer’s instructions. Flow cytometry was performed to quantify tumor cell death, with CFSE^+^PI^+^ cells identified as dead tumor cells.

### Mouse BMDM isolation.

Bone marrow–derived cells were harvested from 6- to 8-week-old C57BL/6 mice. Bone marrow cells were isolated from the femurs and tibias of euthanized mice and passed through a 70 μm cell strainer. After red blood cell lysis, the remaining cells were cultured in the RPMI 1640 medium supplemented with 10% serum and 10 ng/mL recombinant murine M-CSF for 7 days. The medium was changed on days 3 and 5. The purity of CD11b^+^F4/80^+^ macrophages was verified to be 90% by flow cytometry.

### IHC and multiple IF staining.

IHC staining was performed in paraffin-embedded tissues. Mouse GBM tissues were collected from mice when neurological signs occurred after tumor induction. Human GBM specimens were obtained from patients with GBMs through surgical resection. The tumor sections were dewaxed with xylene and hydrated in gradient ethanol. Antigen retrieval was performed using sodium citrate solution. Sections were incubated in 0.3% hydrogen peroxide for 15 minutes. Then slides were blocked with 5% goat serum (Solarbio, SL038) containing 0.3% Triton X-100 for 1 hour. The primary antibody was incubated overnight at 4°C. Sections were incubated with HRP-conjugated secondary antibodies (anti-mouse/rabbit IgG) for 40 minutes. Chromogenic visualization was performed using DAB (AiFang Biological, AFIHC004), and nuclei were counterstained with hematoxylin. Sections were subsequently dehydrated, cleared in xylene, mounted with neutral balsam, and imaged by light microscopy. For multiple IF staining, sections were incubated with TYR-520/570/690 fluorescent dye (AiFang Biological, AFIHC035) for 10 minutes after the HRP-conjugated secondary antibody incubation. Antigen retrieval was repeated, and another primary antibody was incubated overnight at 4°C. Sections were incubated with HRP anti-mouse IgG/anti-rabbit IgG antibody and then incubated with another fluorescent dye for 10 minutes. After washing by PBS, the slides were redyed with DAPI and photographed with a Zeiss confocal microscope.

### ChIP.

Chromatin was first extracted and sonicated in accordance with previous protocols (11). Mice primary cells were fixed and cross-linked in 1% formaldehyde and subjected to sonication to fragment DNA. Chromatin was incubated with antibodies overnight at 4°C. Immunoprecipitated complexes were collected using protein A/G Plus agarose (Beyotime, P2055). The beads were washed, and precipitated DNA was released by proteinase K digestion in DNA release buffer. The purified DNA was subsequently used as a template for qPCR or ChIP-seq.

### Dual-luciferase reporter assay.

For transcriptional inhibition analysis, the mice enhancer binding site of *Cxcl10* was cloned into the pGL3-promoter vector. HEK293T or GL261 cells were transfected with pGL3-*Cxcl10*-promoter plasmid, *Olig2* expression vector, and Renilla expression vectors and incubated for 48 hours. After transfection, cells were harvested, and firefly luciferase activity was measured and normalized to Renilla luciferase activity. Putative *Cxcl10* activity was measured by a dual-luciferase assay.

### RT-qPCR.

Total RNA from cells were extracted using RNAiso Plus reagent according to the manufacturer’s protocol (Takara, 108-95-2). Reverse transcription was performed using commercial reverse transcription kits. RT-qPCR was performed using ChamQ SYBR qPCR Master Mix (Vazyme, Q711). Primer sequences are provided in Supplemental Table 1.

### Western blot.

Total proteins of cells were collected in RIPA buffer containing PMSF and PIC. Lysates were centrifuged for 15 minutes at approximately 13,520*g* and 4°C. The protein lysates were quantified with a BCA kit (Biosharp, BL521A). Lysates containing equal amounts of proteins were separated by SDS-PAGE and transferred onto PVDF membranes. After blocking, membranes were immunoblotted with relative primary antibodies followed by the HRP-conjugated antibody. Following the detection of phosphorylated proteins, membranes were stripped using stripping buffer (Beyotime, P0025) to remove the antibodies. After washing 3 times with TBST, the membranes were reblocked and reprobed with antibodies against the corresponding total proteins. The antibodies are listed in Supplemental Table 2.

### Co-IP.

Total proteins of cells were extracted using IP lysis buffer (Beyotime, P0013-100ML) supplemented with PMSF and PIC for 1 hour and centrifuged at approximately 13,520*g* for 15 minutes. Lysates were incubated by protein A/G Plus agarose beads for 4 hours at 4°C to remove nonspecific binding. Supernatants were incubated with primary antibody or relative isotype IgG overnight at 4°C. Then, lysates were captured by protein A/G Plus agarose beads (Beyotime, P2055) for 4 hours at 4°C. The precipitants were washed with ice-cold PBS 5 times and boiled with SDS loading buffer at 100°C for 8 minutes. Proteins were subjected to SDS-PAGE and analyzed by liquid chromatography–tandem mass spectrometry (LC-MS/MS) or Western blot. A complete list of antibodies is provided in Supplemental Table 2.

### LC-MS/MS analysis.

The proteins from co-IP were separated by SDS-PAGE. The indicated bands at approximately 15–180 kDa were cut and subjected to MS analysis (Applied Protein Technology).

### Molecular docking.

The molecular structures of OLIG2 and HDAC7 were retrieved from the AlphaFold database (https://alphafold.ebi.ac.uk/). Subsequently, the PDB files of both proteins were submitted to the GRAMM web server (Vakser Lab) for protein–protein docking analysis (46). The resulting docking complexes were then analyzed using PDBePISA (https://www.ebi.ac.uk/msd-srv/prot_int/pistart.html), an interactive tool for characterizing macromolecular interfaces. This analysis provided detailed information on intermolecular interactions, including hydrogen bonds, disulfide bonds, salt bridges, and covalent linkages. Finally, PyMOL molecular visualization software (https://www.pymol.org/pymol.html) was employed to generate structural representations of the OLIG2-HDAC7 complex, with the docking interfaces highlighted by dashed lines (yellow arrow in Figure 5F).

### scRNA-seq data generation and quality control.

Details on tissue dissociation and the preparation of single-cell suspension are described in *Isolation of single cells from mouse brain tumors*. Sorted cells were processed for single-cell capture, and cDNA libraries were generated using the BD Rhapsody platform protocol (BD Biosciences). Libraries were sequenced by Novogene. Raw FASTQ files were processed using the BD Rhapsody whole transcriptome analysis pipeline to obtain a unique molecular identifier (UMI) matrix for each sample. The matrix of read counts per sample was further analyzed with the Seurat package (version 4.3.0) in R software (version 4.2.2). For each cell, we used 3 quality control measures. Cells falling into any of the following 3 categories were excluded: (a) <200 expressed genes, (b) >30% UMIs of mitochondrial genes, and (c) >30% UMIs of ribosomal genes.

### Bioinformatic analysis.

Bulk RNA-seq and ChIP-seq analyses were performed using our previous RNA-seq data (GSE80089) or the datasets of TCGA and others downloaded via the Gliovis platform (https://gliovis.bioinfo.cnio.es/). ESTIMATE (https://ibl.mdanderson.org/estimate/) and CIBERSORT were used to analyze the relationship between gene expression and immune infiltration. GSEA (version 4.3.2) was used to analyze the enrichment of signature gene sets from TCGA and our previous RNA-seq data. Correlation analysis was performed using the Gliovis platform.

For scRNA-seq analysis, each dataset was processed through normalization, scaling, and dimensionality reduction via principal component analysis (PCA) using Seurat. UMAP visualization was performed to display cell clusters. Marker genes for cluster characterization were identified with the FindAllMarkers function. Pathway enrichment analysis was conducted with clusterProfiler (version 4.6.2) (47). GSCs were characterized by high expression of SOX2. Cell–cell communication networks and ligand–receptor interactions were analyzed using CellChat (version 2.1.2) (48).

For spatial transcriptomic analysis, a publicly available dataset (GSE276841) was employed to investigate the spatial distribution pattern of macrophages, microglia, CD8A^+^ T cells, CD4^+^ T cells, and tumor cell subtypes (MES1, MES2, AC, NPC1, NPC2, OPC, and undefined) using the cell2location algorithm. First, the scRNA-seq dataset (GSE182109) underwent standard normalization followed by PCA, dimensionality reduction, and unsupervised clustering. Cell cluster identities were determined through differential expression analysis using the FindAllMarkers function in Seurat. Tumor cell subtypes were annotated based on established gene signatures from Neftel et al. (28). Reference transcriptional signatures derived from annotated scRNA-seq cell types were applied to Visium data through the negative binomial regression model implemented in the Python (3.12.9) version of the cell2location package (version 0.1.4). The cell2location model was trained with default parameters to estimate the posterior distribution of cellular abundance for each cell type at every spatial point, which was then used for downstream analysis of distribution features and colocalization. The estimated proportion of a specific cell type was measured as the ratio of spots with cell2location-measured abundance higher than the average across all selected spots. To identify macrophages, microglia, CD8^+^ T cells, CD4^+^ T cells, and tumor subtypes, spots with cellular abundance exceeding the average level of the corresponding cell population across all spots were designated as dominant spots for that cell population.

### Statistics.

GraphPad Prism software was used for data presentation and statistical analysis. Unpaired 2-tailed Student’s *t* test or unpaired or paired 1- or 2-way ANOVA was used with a multiple-comparison test as indicated in the figure legends. Data are shown as the mean ± SEM. Where applicable, a ROUT outlier test (designed to identify 1 or more outliers in a dataset based on nonlinear regression) was performed on data, and identified outliers were removed. Survival analysis was performed by log-rank test. *P* values less than 0.05 were considered significant.

### Study approval.

Human GBM tissues were obtained from The First Affiliated Hospital of Chongqing Medical University. Approval for tissue collection and experimentation was granted by the IRB of Chongqing Medical University (number 2024057). Informed consent was obtained from all patients. All animal procedures employed in this study were approved by the IACUC of Chongqing Medical University (IACUC-CQMU-2023-0429).

### Data availability.

All data and sources of materials are included in the article. Values for all data points in graphs are reported in the Supporting Data Values file. The scRNA-seq data were deposited in the National Genomics Data Center’s Genome Sequence Archive database (CRA031416). The bulk RNA-seq and ChIP-seq datasets are available in the Gene Expression Omnibus (GSE80089). All additional data and analytic code that support the findings of this study are available from the corresponding author FL upon reasonable request.

## Author contributions

FL, QRL, and ZT designed experimental and interpreted data. QRL provided human glioma TS543 cells and C57BL/6 background *Pten*^fl/fl^ *Trp53*^fl/fl^ *Olig2*^fl/fl^ transgenic mice. XZ, C Zhao, JX, C Zeng, JZ, GY, XY, YL, DL, YX, JY, and SC carried out the experiments. JX, XZ, C Zhao, LQ, and HL analyzed the data and prepared the figures. FL, QL, and SH wrote and edited manuscript. FL, QRL, and YD supervised the project.

## Funding support

- National Natural Science Foundation of China (grants 82473030, 82072799, and 32100454 to FL, HL, and ZT).
- Scientific and Technological Research Program of Chongqing Municipal Education Commission (KJZD-K202200410 and KJQN202300434 to FL and LQ).
- Natural Science Foundation of Chongqing (CSTB2022NSCQ-MSX0843 and CSTC2021JCYJ-MSXMX0262 to FL and ZT).
- Chongqing Medical University Program for Youth Innovation in Future Medicine (W0106 to FL and ZT).
- Graduate Student Research Innovation Project in Chongqing (CYB240223 to JX).
- CancerFree Kids Foundation to (QL).

## Supplementary Material

Supplemental data

Unedited blot and gel images

Supporting data values

## Version 1 01/27/2026

In-Press Preview

## Version 2 03/02/2026

Electronic publication

## Version 3 11/13/2026

Correction to figure 4A, supplemental figure 2H, and supporting data values.
