# Corrigendum to Oligodendrocyte transcription factor 2 orchestrates glioblastoma immune evasion by suppressing CXCL10 and CD8+ T cell activation

**DOI:** 10.1172/JCI213755

**Published:** 2026-11-16

**Authors:** Xinchun Zhang, Jinjiang Xue, Cunyan Zhao, Chenqiuyue Zeng, Jiacheng Zhong, Gangfeng Yu, Xi Yang, Yao Ling, Dazhen Li, Jiaxiao Yang, Yun Xiu, Hongda Li, Shiyuan Hong, Liangjun Qiao, Song Chen, Q. Richard Lu, Yaqi Deng, Zhaohua Tang, Fanghui Lu

Original citation: *J Clin Invest*. 2026;136(5):e195556. https://doi.org/10.1172/JCI195556

Citation for this corrigendum: *J Clin Invest*. 2026;136(22):e213755. https://doi.org/10.1172/JCI213755

Following publication of this article, the journal was notified that Figure 4A was a representative subset of the available data. The correct figure 4A, including the complete dataset, appears below and in the updated HTML and PDF versions of the article. In addition, a duplicated data point was identified in supplemental figure 2H. The supplemental data file has been updated with the correct figure, and the supporting data values file has been updated online with correction information for 4A and supplemental figure 2H.

The authors regret the errors.

## Supplementary Material

Supplemental data

Supporting data values

## Figures and Tables

**Figure 4:**
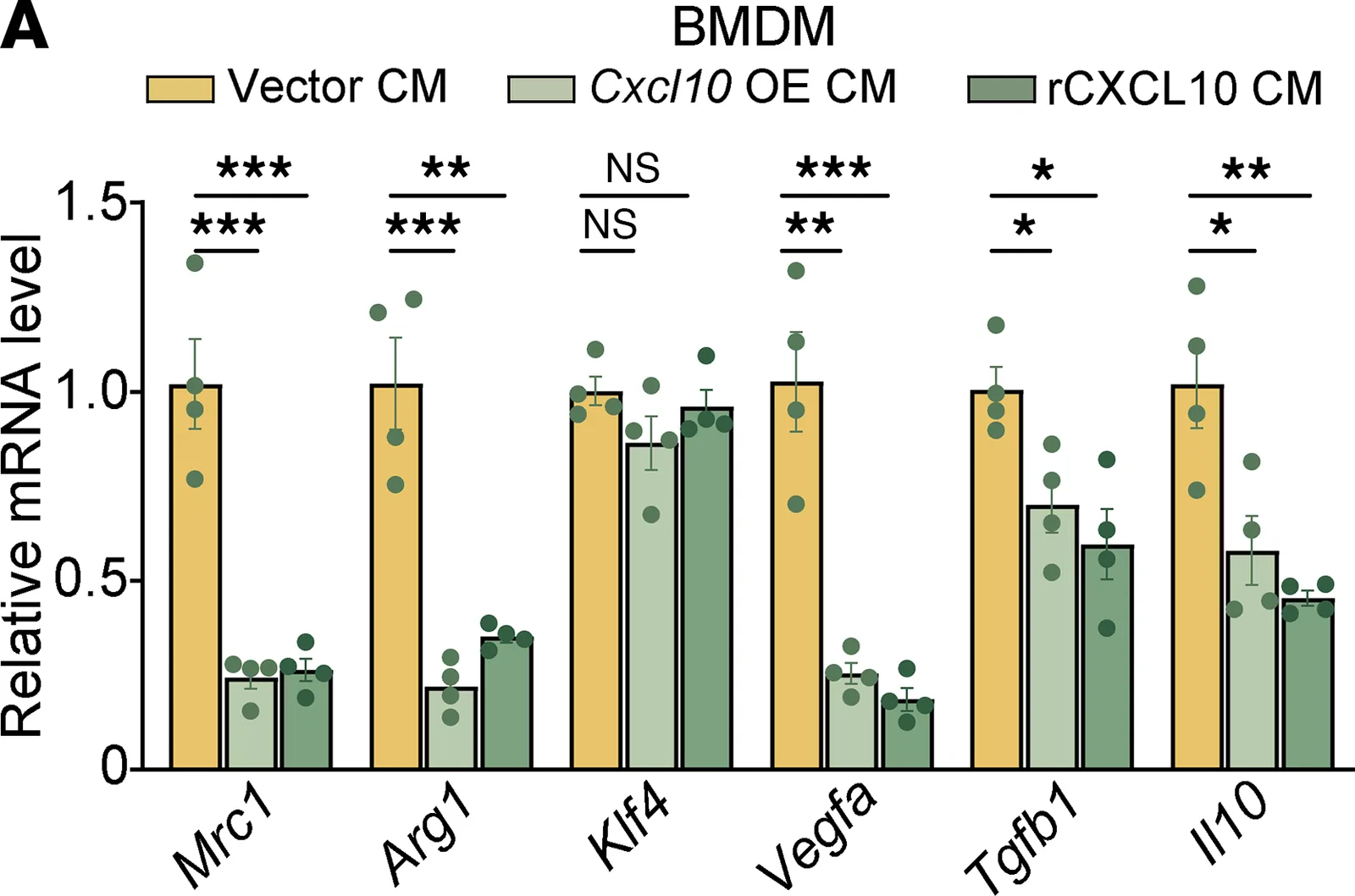
CXCL10 reduces M2-like polarization of macrophages by inhibiting STAT3 signaling. (**A**) qPCR of mRNA expression of antiinflammatory markers in BMDMs treated with CM from the indicated groups (*n* = 4).

## Version 1 11/16/2026

Electronic publication

