# Supplementary material for: Oligodendrocyte transcription factor 2 orchestrates glioblastoma immune evasion by suppressing CXCL10 and CD8+ T cell activation
Source: J Clin Invest. 2026 Jan 27;136(5):e195556. doi: 10.1172/JCI195556 (PMC12948422; doi:10.1172/JCI195556)
Supplement: Supplemental data [file jci-136-195556-s090.pdf]

Oligodendrocyte transcription factor 2 orchestrates glioblastoma immune evasion by suppressing CXCL10 and $CD8^+$ T cell activation

Xinchun Zhang[1,2,#], Jinjiang Xue[1,3,#], Cunyan Zhao[1,4,#], Chenqiuyue Zeng[1,#], Jiacheng Zhong[2], Gangfeng Yu[1], Xi Yang[1], Yao Ling[3], Dazhen Li[1], Jiaxiao Yang[3], Yun Xiu[3], Hongda Li[3], Shiyuan Hong[5], Liangjun Qiao[3], Song Chen[2], Q. Richard Lu[6], Yaqi Deng[3,*], Zhaohua Tang[2,*], Fanghui Lu[1,*]

[1] Department of Cancer Center, The Second Affiliated Hospital of Chongqing Medical University, Chongqing Medical University, Chongqing, 404100, China.

[2] Department of Neurosurgery, Key Laboratory of Major Brain Disease and Aging Research (Ministry of Education), The First Affiliated Hospital of Chongqing Medical University, Chongqing, 400016, China.

[3] School of Basic Medical Sciences, Chongqing Medical University, Chongqing, 400016, China.

[4] Chongqing University Central Hospital, Chongqing, 400014, China.

[5] College of Pharmacy, Chongqing Medical University, Chongqing, 400016, China.

[6] Department of Pediatrics, Division of Experimental Hematology and Cancer Biology, Cincinnati Children's Hospital Medical Center, Cincinnati, OH, 45229, USA.

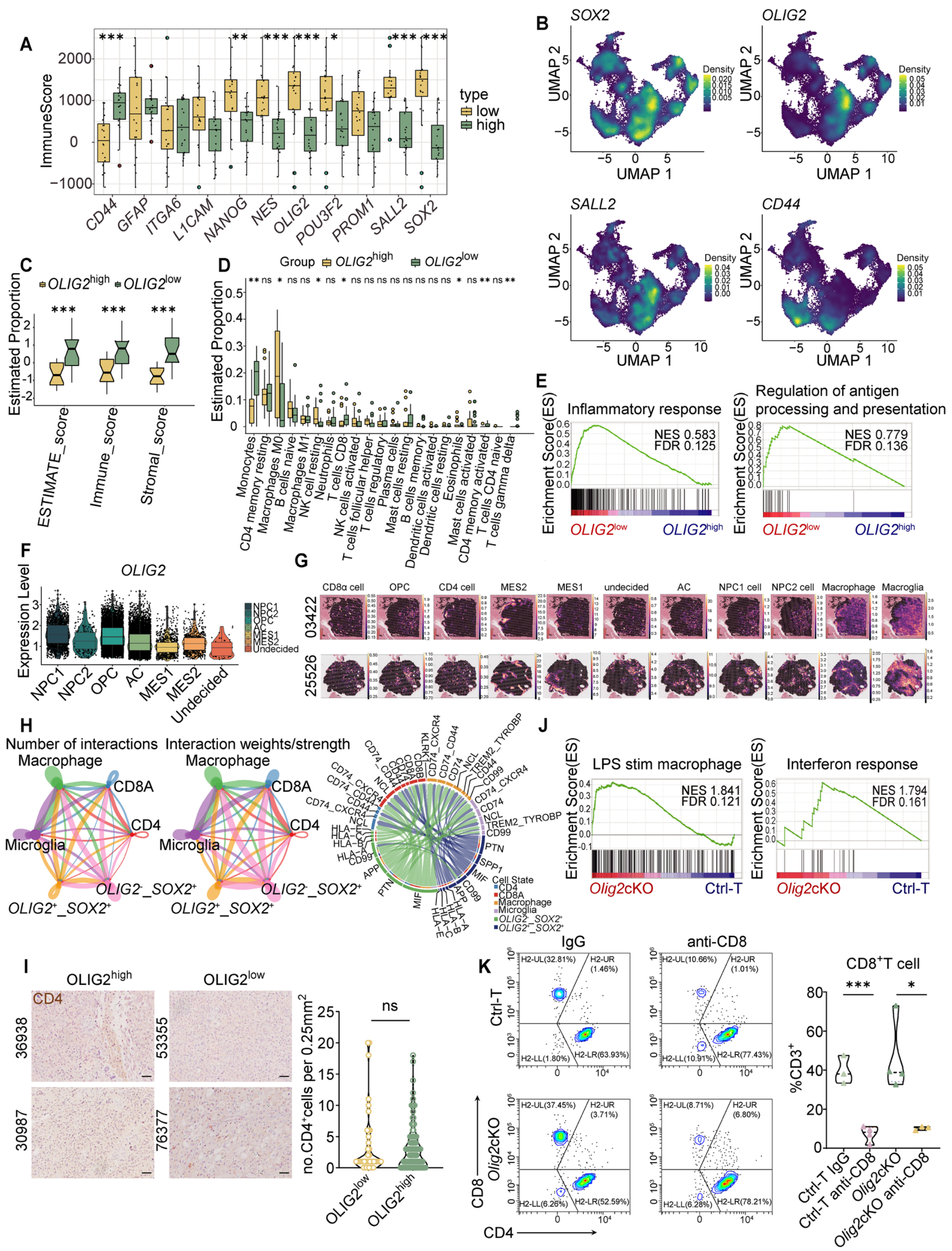

20

Supplementary Figure 1. High OLIG2 expression positively correlates with the pro-tumor immune microenvironment in GBM

(**A**) ESTIMATE analysis of immune infiltrations in GBM patients from TCGA database with high or low expression of GSC markers (*n*=40). (**B**) UMAP visualization of the specific GSC markers distribution in GBM cells (GSE182109). (**C**) ESTIMATE analysis of immune infiltrations in GBM patients from TCGA database with high or low *OLIG2* expression (*n*=40). (**D**) Characterization of the immune cell composition in GBM from TCGA databases by CIBERSORT analysis (*n*=40). (**E**) GSEA showing the enrichment of signature gene sets of immunoregulation in *OLIG2*^{low} GBM cases compared with *OLIG2*^{high} specimen (*n*=40). (**F**) The *OLIG2* expression levels in different subtypes of tumor cells (GSE182109). (**G**) Spatial feature plots showing the scores of cell types in GBM slides (GSE276841). (**H**) Cell-cell communications among *OLIG2*$^{+}$*SOX2*$^{+}$ GSC, *OLIG2*$^{-}$*SOX2*$^{+}$ GSC and immune cells from GBM scRNA-seq dataset (GSE182109) by CellChat. (**I**) Representative staining and quantification for CD4 (*n*=164) in GBM cases with high or low OLIG2 expression. Scale bar: 50 μm. Each plot represents the number of CD4 per area (0.25 mm^2). (**J**) GSEA showing the enrichment of signature gene sets of LPS-stimulated macrophage and Interferon response in GBM of *Olig2*cKO mice compared with Ctrl-T mice. (**K**) The efficiency of CD8$^+$ T cells depletion in mouse spleen (*n*=3-4 mice/group). Unpaired Student's *t*-test in **C**, **D**, **H** and **J**. *$p < 0.05$, **$p < 0.01$, ***$p < 0.001$, ns, not significant.

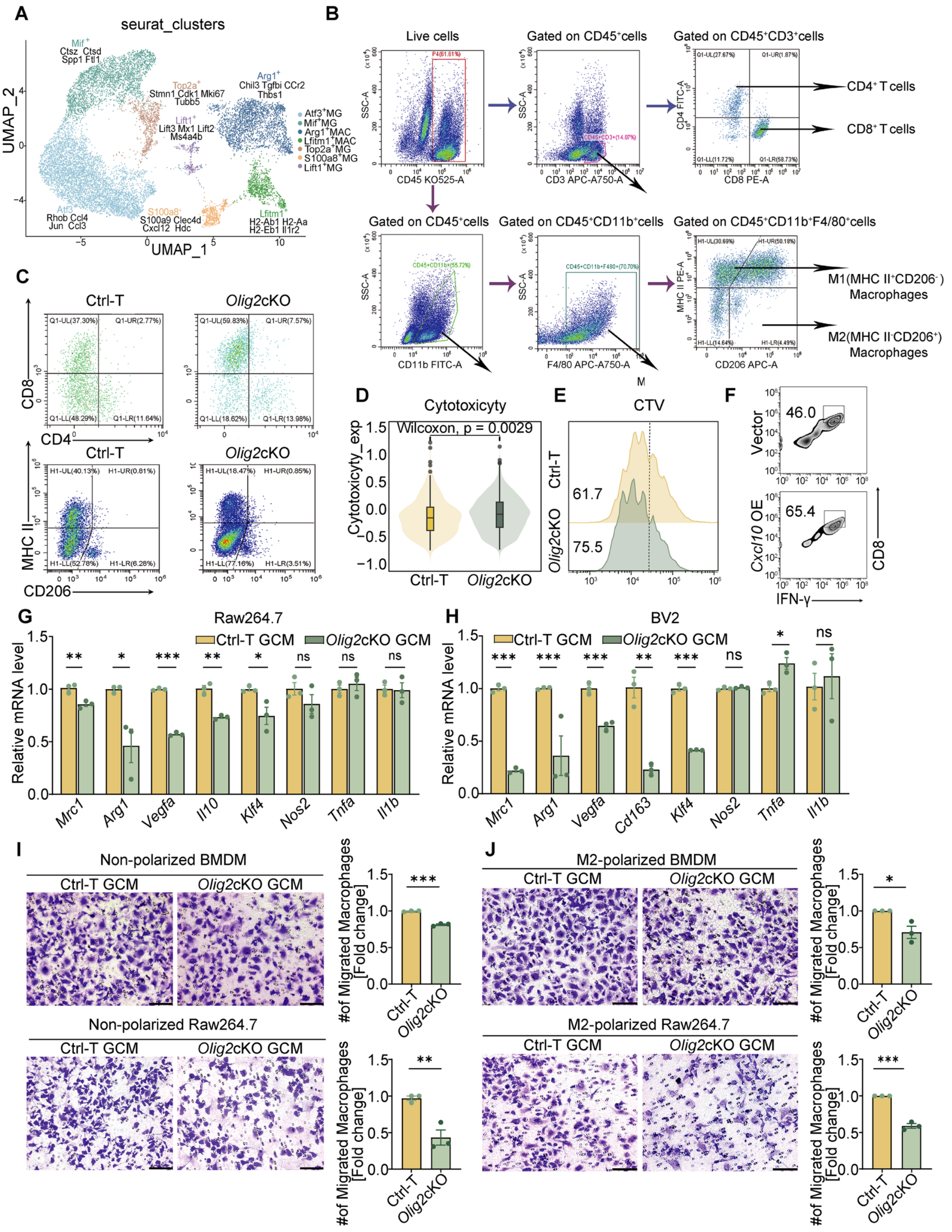
A
seurat_clusters
B
Live cells
Gated on CD45+cells
Gated on CD45+CD3+cells
CD4+ T cells
CD8+ T cells
Gated on CD45+cells
Gated on CD45+CD11b+cells
Gated on CD45+CD11b+F4/80+cells
M1(MHC II+CD206-) Macrophages
M2(MHC II-CD206+) Macrophages
C
Ctrl-T
Olig2cKO
D
Cytotoxicyty
Wilcoxon, p = 0.0029
E
CTV
F
Vector
Cxcl10 OE
IFN-γ
CD8
G
Raw264.7
Ctrl-T GCM
Olig2cKO GCM
Relative mRNA level
H
BV2
I
Non-polarized BMDM
Non-polarized Raw264.7
#of Migrated Macrophages [Fold change]
J
M2-polarized BMDM
M2-polarized Raw264.7

41

Supplementary Figure 2. OLIG2 deletion enhances T cells activation and inhibits protumor TAMs in GBM

(**A**) UMAP of scRNAseq data from TAMs and signature genes for each TAM cluster were labeled. (**B**) Gating strategy for $CD8^+$ T cells, $CD4^+$ T cells, MHC II^-CD206^+ TAMs, and MHC II^+CD206^- TAMs in mouse GBM. (**C**) Representative flow cytometry scatter plots of the $CD8^+$ T cells, $CD4^+$ T cells, MHC II^-CD206^+ TAMs, and MHC II^+CD206^- TAMs in Ctrl-T and *Olig2*cKO mice. (**D**) Violin-box plots depicting the cytotoxicity score of the $CD8^+$ T cells using scRNA seq data from Ctrl-T and *Olig2*cKO groups. Two-tailed unpaired Wilcoxon rank-sum test. (**E**) Representative histograms for CellTrace Violet proliferation assay of $CD8^+$ T cells treated with CM from Ctrl-T and *Olig2*cKO tumor cells for 72h (n=3). (**F**) Representative plots for IFN-γ expression of $CD8^+$ T cells treated with CM from Ctrl-T and *Olig2*cKO tumor cells (n=3). (**G**, **H**) qPCR for mRNA expression of anti-tumor and pro-tumor markers in Raw264.7 and BV2 cells treated with Ctrl-T and *Olig2*cKO CM (n=3). (**I**, **J**) Representative images and quantifications of migrated non- or M2-polarized Raw264.7 and BMDMs toward Ctrl-T CM or *Olig2*cKO CM by Transwell assay (n=3). Scale bar: 100 μm. Unpaired Student's t-test in **G**-**J**. *$p < 0.05$, **$p < 0.01$, ***$p < 0.001$, ns, not significant.

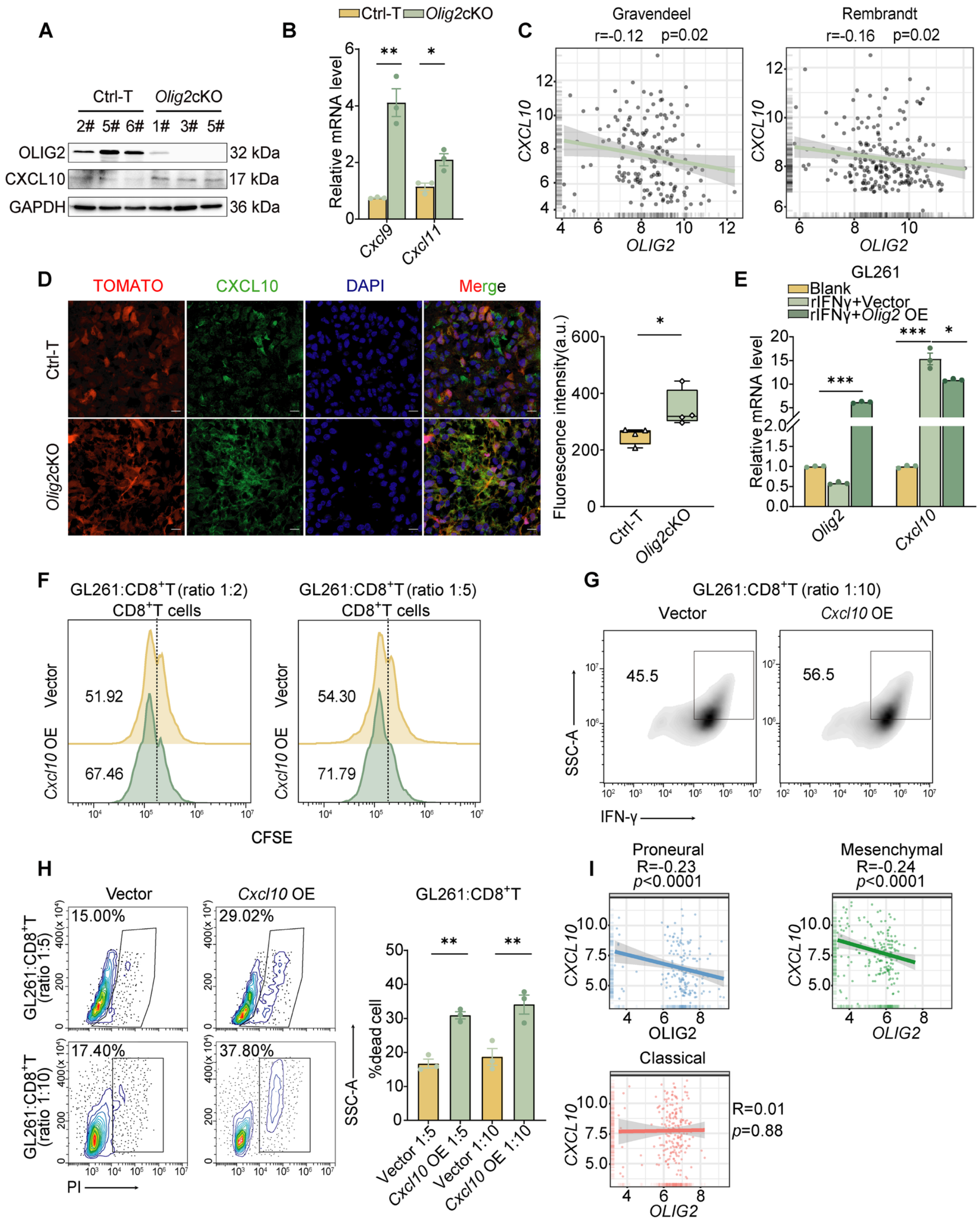
A
Ctrl-T
Olig2cKO
2# 5# 6# 1# 3# 5#
OLIG2
CXCL10
GAPDH
32 kDa
17 kDa
36 kDa
B
Ctrl-T
Olig2cKO
Relative mRNA level
Cxcl9
Cxcl11
C
Gravendeel
r=-0.12 p=0.02
Rembrandt
r=-0.16 p=0.02
CXCL10
OLIG2
D
TOMATO
CXCL10
DAPI
Merge
Ctrl-T
Olig2cKO
Fluorescence intensity(a.u.)
E
GL261
Blank
rIFNγ+Vector
rIFNγ+Olig2 OE
Relative mRNA level
Olig2
Cxcl10
F
GL261:CD8+T (ratio 1:2)
CD8+T cells
GL261:CD8+T (ratio 1:5)
CD8+T cells
Vector
Cxcl10 OE
51.92
67.46
54.30
71.79
CFSE
G
GL261:CD8+T (ratio 1:10)
Vector
Cxcl10 OE
45.5
56.5
SSC-A
IFN-γ
H
Vector
Cxcl10 OE
GL261:CD8+T (ratio 1:5)
GL261:CD8+T (ratio 1:10)
15.00%
29.02%
17.40%
37.80%
PI
SSC-A
GL261:CD8+T
%dead cell
Vector 1:5
Cxcl10 OE 1:5
Vector 1:10
Cxcl10 OE 1:10
I
Proneural
R=-0.23
p<0.0001
Mesenchymal
R=-0.24
p<0.0001
Classical
R=0.01
p=0.88
CXCL10
OLIG2

Supplementary Figure 3. OLIG2 regulates *CXCL10* expression to modulate T Cell proliferation and IFN-γ secretion

(**A**) Western blot for CXCL10 protein expression in Ctrl-T and *Olig2*cKO GBM cells. (**B**) qPCR for mRNA expression levels of *Cxcl9/11* in Ctrl-T and *Olig2*cKO GBM primary cells (*n*=3). (**C**) Correlation between *OLIG2* expression and *CXCL10* expression in Gravendeel and Rembrandt database. (**D**) Representative images and quantification of CXCL10 staining from Ctrl-T and *Olig2*cKO mice. Scale bar: 10 μm. (**E**) qPCR for mRNA expression of *Cxcl10* in 20ng/mL IFN-γ stimulated GL261 cells upon *Olig2* overexpression (*n*=3). (**F**) Representative histograms for CFSE proliferation assay of $CD8^+$ T cells co-cultured with GL261-Ctrl and GL261-*Cxcl10* OE cells at the indicated ratio for 72h (*n*=3). (**G**) Representative plots for IFN-γ expression of $CD8^+$ T cells treated with CM from GL261-Ctrl and GL261-*Cxcl10* OE cells for 24h (*n*=3). (**H**) Representative plots and quantification for the PI-positive tumor cells in GL261-Ctrl and GL261-*Cxcl10* OE groups cocultured with $CD8^+$ T cell at the indicated ratio for 72h (*n*=3). (**I**) Correlation between *OLIG2* and *CXCL10* expressions in TCGA GBM dataset. Unpaired Student's *t*-test in **B**, One-way ANOVA in **E, H**. *$p < 0.05$, **$p < 0.01$, ***$p < 0.001$, ns, not significant.

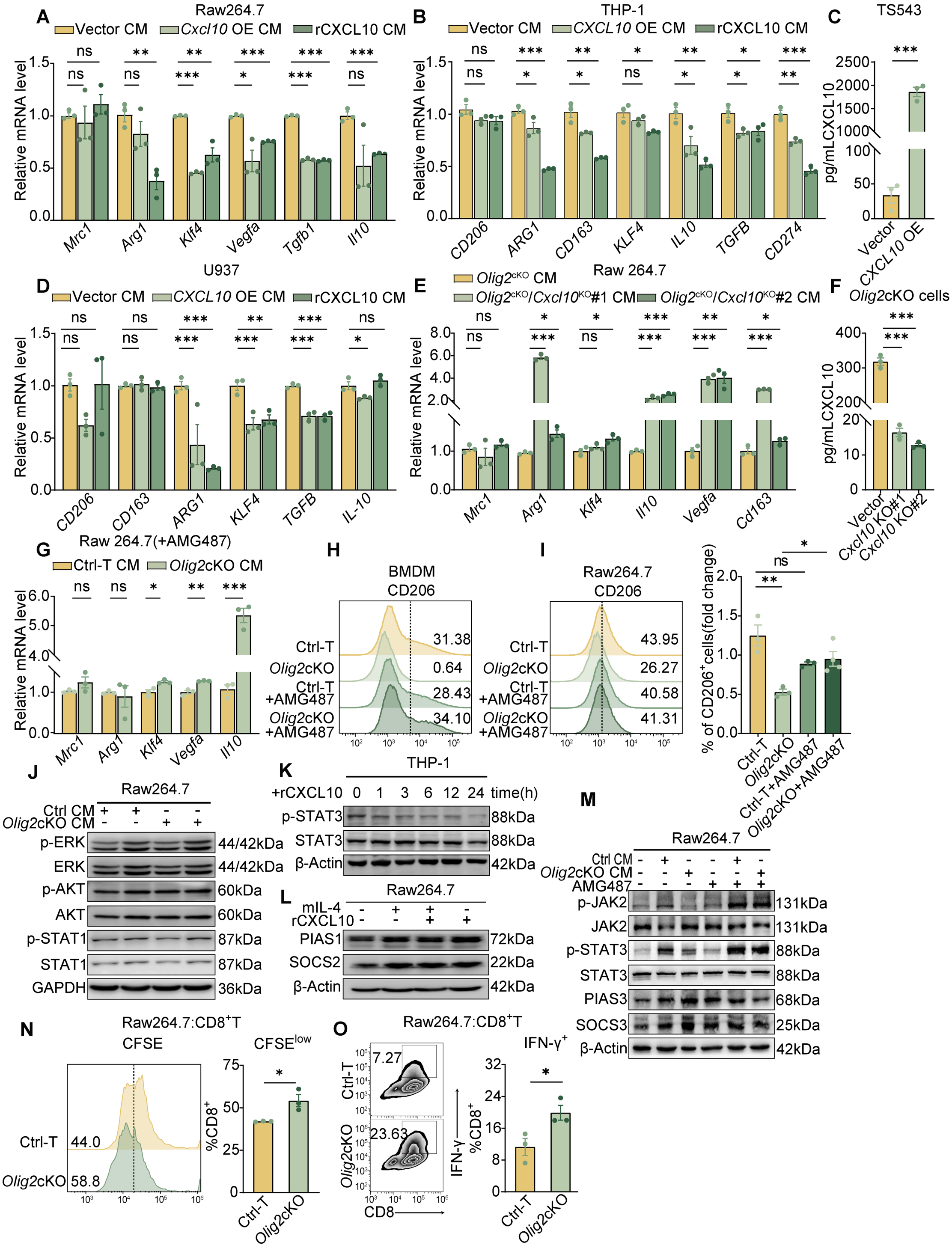
A
Raw264.7
Vector CM
Cxcl10 OE CM
rCXCL10 CM
Relative mRNA level
Mrc1
Arg1
Klf4
Vegfa
Tgfb1
Il10
B
THP-1
Vector CM
CXCL10 OE CM
rCXCL10 CM
CD206
ARG1
CD163
KLF4
IL10
TGFB
CD274
C
TS543
pg/mLCXCL10
Vector
CXCL10 OE
D
U937
Vector CM
CXCL10 OE CM
rCXCL10 CM
CD206
CD163
ARG1
KLF4
TGFB
IL-10
E
Raw 264.7
Olig2cKO CM
Olig2cKO/Cxcl10KO#1 CM
Olig2cKO/Cxcl10KO#2 CM
Mrc1
Arg1
Klf4
Il10
Vegfa
Cd163
F
Olig2cKO cells
Vector
Cxcl10 KO#1
Cxcl10 KO#2
G
Raw 264.7(+AMG487)
Ctrl-T CM
Olig2cKO CM
Mrc1
Arg1
Klf4
Vegfa
Il10
H
BMDM
CD206
Ctrl-T
Olig2cKO
Ctrl-T +AMG487
Olig2cKO +AMG487
31.38
0.64
28.43
34.10
I
Raw264.7
CD206
Ctrl-T
Olig2cKO
Ctrl-T +AMG487
Olig2cKO +AMG487
43.95
26.27
40.58
41.31
% of CD206+cells(fold change)
Ctrl-T
Olig2cKO
Ctrl-T+AMG487
Olig2cKO+AMG487
J
Raw264.7
Ctrl CM
Olig2cKO CM
p-ERK 44/42kDa
ERK 44/42kDa
p-AKT 60kDa
AKT 60kDa
p-STAT1 87kDa
STAT1 87kDa
GAPDH 36kDa
K
THP-1
+rCXCL10 0 1 3 6 12 24 time(h)
p-STAT3 88kDa
STAT3 88kDa
β-Actin 42kDa
L
Raw264.7
mIL-4
rCXCL10
PIAS1 72kDa
SOCS2 22kDa
β-Actin 42kDa
M
Raw264.7
Ctrl CM
Olig2cKO CM
AMG487
p-JAK2 131kDa
JAK2 131kDa
p-STAT3 88kDa
STAT3 88kDa
PIAS3 68kDa
SOCS3 25kDa
β-Actin 42kDa
N
Raw264.7:CD8+T
CFSE
CFSElow
Ctrl-T 44.0
Olig2cKO 58.8
%CD8+
Ctrl-T
Olig2cKO
O
Raw264.7:CD8+T
IFN-γ+
Ctrl-T
Olig2cKO
7.27
23.63
CD8
IFN-γ
%CD8+
Ctrl-T
Olig2cKO

Supplementary Figure 4. CXCL10 attenuates M2 macrophage polarization through STAT3 signaling suppression

(**A**) qPCR for mRNA expression of anti-inflammatory markers in Raw264.7 cells treated with CM from the indicated groups for 48h (n=3). (**B**) qPCR for mRNA expression of anti-inflammatory markers in THP-1 cells treated with CM from the indicated groups for 48h (n=3). (**C**) ELISA for CXCL10 secretion levels in the supernatant of TS543-Vector and TS543-*CXCL10* OE cells (n=3). (**D**) qPCR for mRNA expression of anti-inflammatory markers in U937 cells treated with CM from the indicated groups for 48h (n=3). (**E**) qPCR for mRNA expression of anti-inflammatory markers in Raw264.7 cells treated with CM from the indicated groups for 48h (n=3). (**F**) ELISA for CXCL10 secretion levels in the supernatant of *Olig2*cKO and *Olig2*cKO-*Cxcl10* KO cells (n=3). (**G**) qPCR for mRNA expression of anti-inflammatory markers in Raw264.7 cells treated with Ctrl-T and *Olig2*cKO CM containing 1 μM AMG487 for 48h (n=3). (**H**) Representative histograms for the CD206 expression of BMDMs treated with Ctrl-T and *Olig2*cKO CM containing AMG487 for 24h (n=3). (**I**) Representative histograms and quantification for the CD206 expression of Raw264.7 cells treated with Ctrl-T and *Olig2*cKO CM containing AMG487 for 24h (n=3). (**J**) Western blot for the protein levels in Raw264.7 cells treated with Ctrl-T and *Olig2*cKO CM. (**K**) Western blot for the protein levels in THP-1 cells treated with 100 ng/mL rCXCL10 proteins at indicated time points. (**L**) Western blot for the protein levels in Raw264.7 cells treated with mIL-4 and rCXCL10 proteins. (**M**) Western blot for the protein levels in Raw264.7 cells co-cultured with Ctrl-T and *Olig2*cKO CM containing AMG487. (**N**) Representative histograms for CFSE proliferation assay of the $CD8^+$ T cells co-cultured with BMDMs pre-incubated with Ctrl-T and *Olig2*cKO CM (n=3). (**O**) Representative flow cytometry for the IFN-γ expression of $CD8^+$ T cells co-cultured with BMDMs pre-incubated with Ctrl-T and *Olig2*cKO CM (n=3). One-way ANOVA in **F**, **I**, Unpaired Student's t-test in **A**-**E**, **G**, **N**, **O**. *$p < 0.05$, **$p < 0.01$, ***$p < 0.001$, ns, not significant.

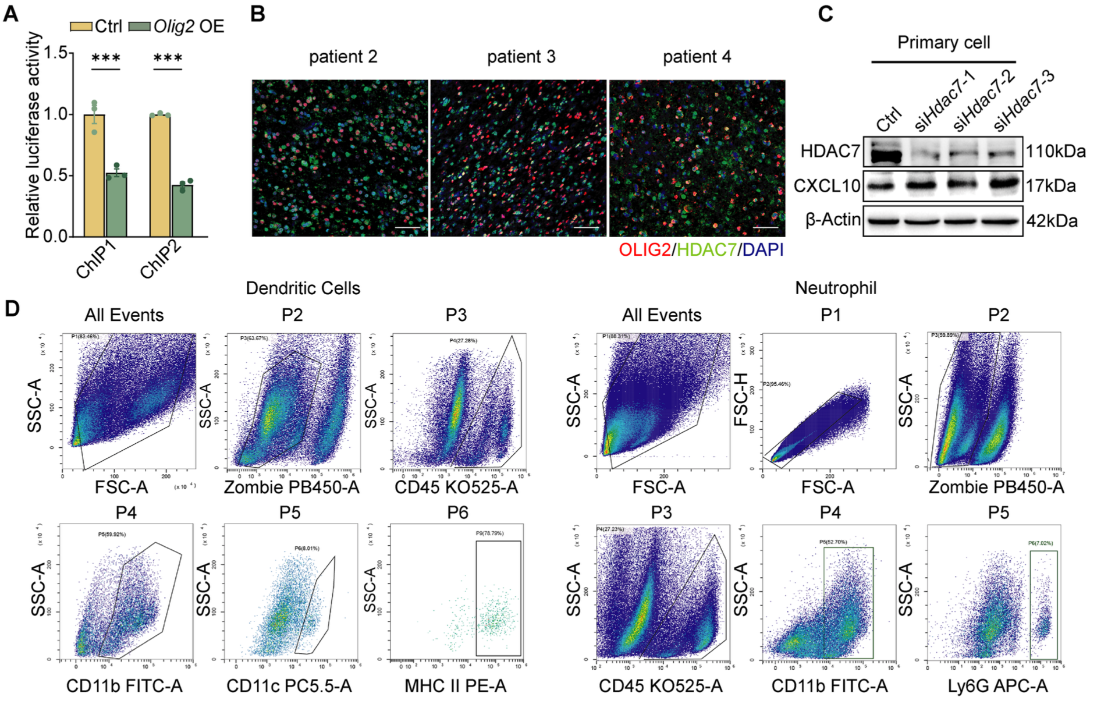

Supplementary Figure 5. OLIG2 recruits HDAC7 to epigenetically silence *CXCL10* expression

(**A**) Dual-luciferase reporter assay for *Cxcl10* enhancer activity in control or *Olig2* overexpressed 293T cells (*n*=3). (**B**) Representative immunofluorescence images showing OLIG2 (red) and HDAC7 (green) signals from three GBM patient specimen. Scale bars = 50 μm. (**C**) Western blot for protein levels of HDAC7 and CXCL10 in Ctrl-T tumor cells transfected with si*Hdac7*. (**D**) Gating strategy for $CD11b^{+}CD11c^{+}MHC\ II^{+}$ dendritic cells and $CD11b^{+}Ly6G^{+}$ neutrophils in mouse GBM for Figure 2B. Unpaired Student's *t*-test in **A**. $^{*}p < 0.05$; $^{**}p < 0.01$; $^{***}p < 0.001$; ns, not significant.

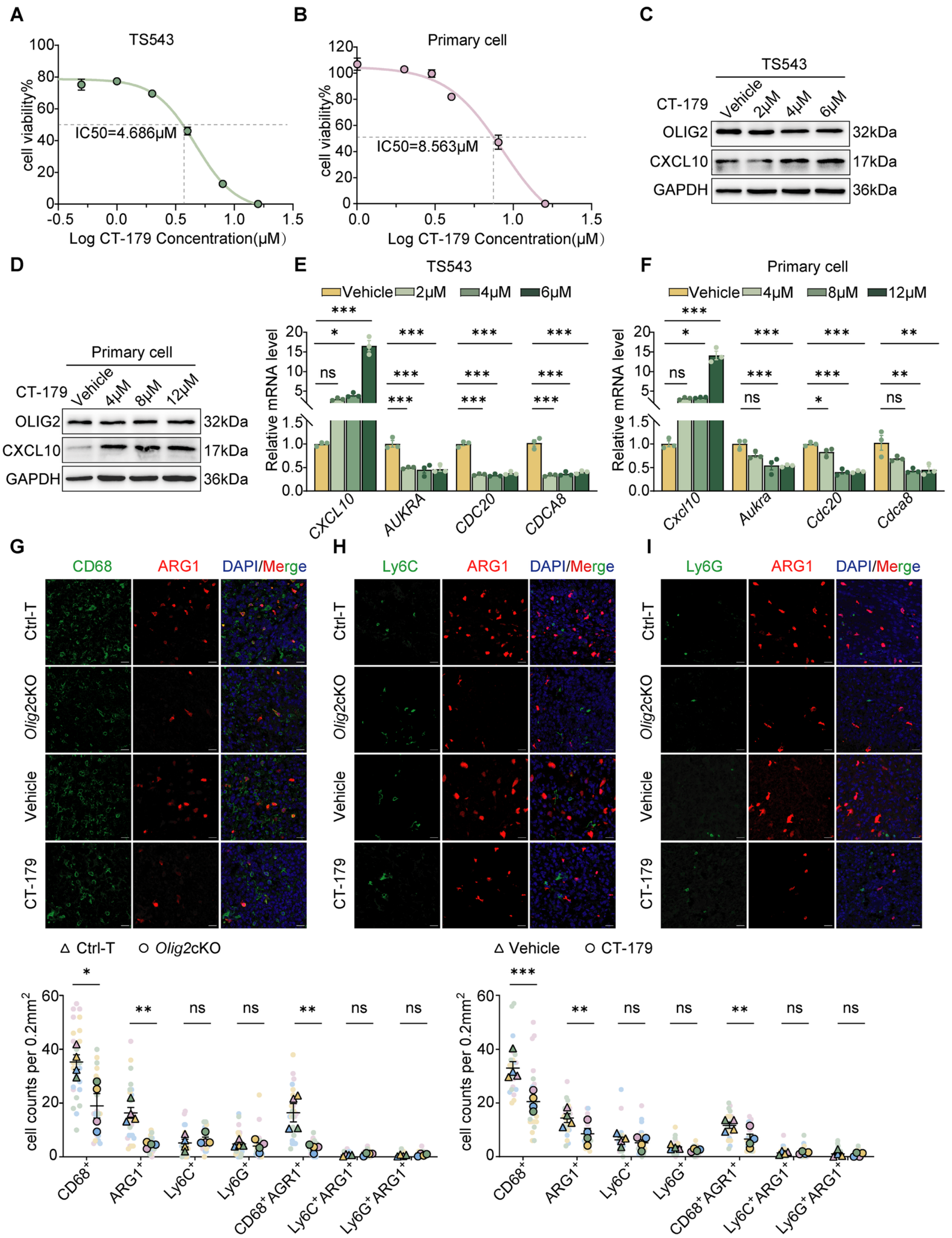
A
TS543
cell viability%
IC50=4.686μM
Log CT-179 Concentration(μM)
B
Primary cell
cell viability%
IC50=8.563μM
Log CT-179 Concentration(μM)
C
TS543
CT-179 Vehicle 2μM 4μM 6μM
OLIG2 32kDa
CXCL10 17kDa
GAPDH 36kDa
D
Primary cell
CT-179 Vehicle 4μM 8μM 12μM
OLIG2 32kDa
CXCL10 17kDa
GAPDH 36kDa
E
TS543
Vehicle 2μM 4μM 6μM
Relative mRNA level
CXCL10 AUKRA CDC20 CDCA8
F
Primary cell
Vehicle 4μM 8μM 12μM
Relative mRNA level
Cxcl10 Aukra Cdc20 Cdca8
G
CD68 ARG1 DAPI/Merge
Ctrl-T
Olig2cKO
Vehicle
CT-179
H
Ly6C ARG1 DAPI/Merge
Ctrl-T
Olig2cKO
Vehicle
CT-179
I
Ly6G ARG1 DAPI/Merge
Ctrl-T
Olig2cKO
Vehicle
CT-179
Ctrl-T Olig2cKO
cell counts per 0.2mm²
CD68⁺ ARG1⁺ Ly6C⁺ Ly6G⁺ CD68⁺AGR1⁺ Ly6C⁺ARG1⁺ Ly6G⁺ARG1⁺
Vehicle CT-179
cell counts per 0.2mm²
CD68⁺ ARG1⁺ Ly6C⁺ Ly6G⁺ CD68⁺AGR1⁺ Ly6C⁺ARG1⁺ Ly6G⁺ARG1⁺

119

Supplementary Figure 6. CT-179 suppresses OLIG2 transcriptional activity and reduces ARG1$^+$ TAMs in mouse GBM

(**A**, **B**) Cell viability assays for TS543 cells and mouse primary GBM cells exposed to CT-179 for 48h. (**C**, **D**) Western blot for OLIG2 and CXCL10 protein expressions in TS543 cells and mouses primary GBM cells treated with CT-179 for 48h. (**E**, **F**) qPCR for mRNA levels of the classical target genes of OLIG2 in TS543 GBM cells and mouse primary GBM cells treated with Vehicle or CT-179 for 48 hours (n=3). (**G-I**) (Top) Representative immunofluorescence images of tumor sections from four experimental groups: Ctrl-T, *Olig2*cKO, Vehicle, and CT-179 (20 mg kg^{-1}). CD68$^+$ macrophages (green), ARG1$^+$ cells (red) in **G**; Ly6C$^+$ monocytes (green), ARG1$^+$ cells (red) in **H**; Ly6G$^+$ neutrophils (green), ARG1$^+$ cells (red) in **I**. (Bottom) Quantitative analysis of immunosuppressive myeloid cell densities. Scale bars = 20 μm; n = 4 mice/group. Data was presented as mean ± SEM. One-way ANOVA in **E**, **F**. Unpaired Student's t-test in **G-I**, *$p<0.05$, **$p<0.01$, ***$p<0.001$, ns, not significant.

Supplementary Table 1

Name	Sequence
**Primers for qPCR**	
q-m*Il1b*-F	GCAACTGTTCCTGAACTCAACT
q-m*Il1b*-R	ATCTTTTGGGGTCCGTCAACT
q-m*Tnfa*-F	GACGTGGAACTGGCAGAAGAG
q-m*Tnfa*-R	TTGGTGGTTTGTGAGTGTGAG
q-m*Arg1*-F	CTCCAAGCCAAAGTCCTTAGAG
q-m*Arg1*-R	AGGAGCTGTCATTAGGGACATC
q-m*Il10*-F	GCTCTTACTGACTGGCATGAG
q-m*Il10*-R	CGCAGCTCTAGGAGCATGTG
q-m*Klf4*-F	GTGCCCCGACTAACCGTTG
q-m*Klf4*-R	GTCGTTGAACTCCTCGGTCT
q-m*Vegfa*-F	GCACATAGAGAGAATGAGCTTCC
q-m*Vegfa*-R	CTCCGCTCTGAACAAGGCT
q-m*Nos2*-F	GTTCTCAGCCCAACAATACAAGA
q-m*Nos2*-R	GTGGACGGGTCGATGTCAC
q-m*Cd163*-F	ATGGGTGGACACAGAATGGTT
q-m*Cd163*-R	CAGGAGCGTTAGTGACAGCAG
q-m*Tgfb1*-F	CTCCCGTGGCTTCTAGTGC
q-m*Tgfb1*-R	GCCTTAGTTTGGACAGGATCTG
q-m*β-Actin*-F	GGCTGTATTCCCCTCCATCG
q-m*β-Actin*-R	CCAGTTGGTAACAATGCCATGT
q-m*Mrc1*-F	CTCTGTTCAGCTATTGGACGC
q-m*Mrc1*-R	CGGAATTTCTGGGATTCAGCTTC
q-m*CD274*-F	GCTCCAAAGGACTTGTACGTG
q-m*CD274*-R	TGATCTGAAGGGCAGCATTTC
q-m*Vegfb*-F	GCCAGACAGGGTTGCCATAC
q-m*Vegfb*-R	GGAGTGGGATGGATGATGTCAG
q-m*Cxcl10*-F	CCAAGTGCTGCCGTCATTTTC
q-m*Cxcl10*-R	GGCTCGCAGGGATGATTTCAA
q-m*Cxcl9*-F	TCCTTTTGGGCATCATCTTCC
q-m*Cxcl9*-R	TTTGTAGTGGATCGTGCCTCG
q-m*Cxcl11*-F	GGCTTCCTTATGTTCAAACAGGG
q-m*Cxcl11*-R	GCCGTTACTCGGGTAAATTACA
q-m*Tnc*-F	ACGGCTACCACAGAAGCTG
q-m*Tnc*-R	ATGGCTGTTGTTGCTATGGCA
q-m*Il17ra*-F	AGTGTTTCCTCTACCCAGCAC
q-m*Il17ra*-R	GAAAACCGCCACCGCTTAC
q-m*Sema3e*-F	AGGCTACGCCTGTCACATAAA
q-m*Sema3e*-R	CCGTTCTTGATACTCATCCAGC
q-m*Adm*-F	CACCCTGATGTTATTGGGTTCA

q-m*Adm*-R	TTAGCGCCCACTTATTCCACT
q-H-*ACTIN*-F	CATGTACGTTGCTATCCAGGC
q-H-*ACTIN*-R	CTCCTTAATGTCACGCACGAT
q-H-*OLIG2*-F	GCTCCTCAAATCGCATCCA
q-H-*OLIG2*-R	AAAGGTCATCGGGCTCTG
q-H-*CXCL10*-F	GTGGCATTCAAGGAGTACCTC
q-H-*CXCL10*-R	TGATGGCCTTCGATTCTGGATT
q-H-*IL-10*-F	TCACATGCGCCTTGATGTCTG
q-H-*IL-10*-R	GACTTTAAGGGTTACCTGGGTTG
q-H-*TNFa*-F	TCGAGAAGATGATCTGACTGCC
q-H-*TNFa*-R	GCTGCACTTTGGAGTGATCG
q-H-*CD206*-F	TTTCTTGTCTGTTGCCGTAGTT
q-H-*CD206*-R	GGGTTGCTATCACTCTCTATGC
q-H-*Arg1*-R	CCAGTCCGTCAACATCAAAACT
q-H-*Arg1*-F	TGGACAGACTAGGAATTGGCA
q-H-*Klf4*-F	CGGACATCAACGACGTGAG
q-H-*Klf4*-R	GACGCCTTCAGCACGAACT
q-H-*TGFB1*-F	GGCCAGATCCTGTCCAAGC
q-H-*TGFB1*-R	GTGGGTTTCCACCATTAGCAC
q-H-*CD163*-F	TTTGTCAACTTGAGTCCCTTCAC
q-H-*CD163*-R	TCCCGCTACACTTGTTTTCAC
q-H-*CXCR3*-F	TTTGACCGCTACCTGAACATAGT
q-H-*CXCR3*-R	GGGAAGTTGTATTGGCAGTGG
q-H-*HDAC7*-F	GGCGGCCCTAGAAAGAACAG
q-H-*HDAC7*-R	CTTGGGCTTATAGCGCAGCTT
**sgRNA**	
Crispr1-m*Cxcl10* F	CACCGGCGGACCGTCCTTGCGAGA
Crispr1-m*Cxcl10* R	AAACTCCTGCGAGGAACGGTCCGCC
Crispr2-m*Cxcl10* F	CACCGGGGAGGGACGTTTATCGGCC
Crispr2-m*Cxcl10* R	AAACGCCGATAAACGTCCCTCCCC
**siRNA**	
m-si-*Hdac7*-1-s	GGCUGGAAACAGAAACCCA
m-si-*Hdac7*-1-a	UGGGUUUCUGUUUCCAGCC
m-si-*Hdac7*-2-s	CGGUCAUGCUGAAGCACCA
m-si-*Hdac7*-2-a	UGGUGCUUCAGCAUGACCG
m-si-*Hdac7*-3-s	GGAUAGUCGUGAUGCCCAU
m-si-*Hdac7*-3-a	AUGGGCAUCACGACUAUCC
H-si-*HDAC7*-1-s	GCAGUGUGGUCAAGCAGAA
H-si-*HDAC7*-1-a	UUCUGCUUGACCACACUGC
H-si-*HDAC7*-2-s	GGCAGGCUUACACCAGCAA
H-si-*HDAC7*-2-a	UUGCUGGUGUAAGCCUGCC

H-si-*HDAC7*-3-s	CCUGAAGUUGCGCUACAAA
H-si-*HDAC7*-3-a	UUUGUAGCGCAACUUCAGG

Supplementary Table 2

The information of antibody		
Antibodies	Source	Catalogue number
Anti-HLA-DMB	Abcam	#ab131273
Anti-Iba1	Abcam	#ab5076
Anti-Acetyl-Histone H3-K27	ABclonal	#A7253
Anti-HDAC7	ABclonal	#A13008
Anti-CXCL10	ABclonal	#A19138
Anti-β-Tubulin	ABclonal	#AC008
HRP-Mouse Anti-Rabbit IgG Light Chain	ABclonal	#AS061
PerCP/Cy5.5 anti-mouse CD8α(clone 53-6.7)	BD Biosciences	#561109
BV510 anti-mouse CD45 (clone 30-F11)	BioLegend	#103138
FITC anti-mouse/human CD11b (clone M1/70)	BioLegend	#101205
APC-Cy7 anti-mouse F4/80 (clone BM8)	BioLegend	#123117
APC anti-mouse CD206 (clone C068C2)	BioLegend	#141707
APC-Cy7 anti-mouse CD3 (clone 17A2)	BioLegend	#100222
APC anti-mouse CD4 (clone GK1.5)	BioLegend	#100405
PE-Cy7 anti-mouse PD-1 (clone RMP1-30)	BioLegend	#109109
APC anti-mouse IFN-γ (clone XMG1.2)	BioLegend	#505810
PerCP/Cy5.5 anti-mouse CD11c (clone N418)	BioLegend	#117327
BV605 anti-mouse NK1.1 (clone PK136)	BioLegend	#108739
APC anti-mouse Ly-6G (clone 1A8)	BioLegend	#127613
Purified anti-mouse CD3ε	BioLegend	#100340
Purified anti-mouse CD28	BioLegend	#117003
Zombie Violet	BioLegend	#423113
Anti-Ly6G	BioLegend	#127601
Anti-CD68	Bio-Rad	#MCA1957T
Anti-CD4	BOSTER	#A00344-2
Anti-CD8α	CST	#D8A8Y
Anti-CD8α	CST	#98941
Anti-p-ERK1/2	CST	#4370S
Anti-ERK	CST	#4695S
Anti-AKT	CST	#9272S
Anti-p-AKT	CST	#9271S
PE anti-mouse MHC II (clone M5/114.15.2)	Invitrogen	#12-5321-81
Rabbit IgG isotype control	Invitrogen	#02-6102
Donkey anti-Mouse IgG (H+L), Alexa Fluor™ Plus 488	Invitrogen	#A32766TR
Donkey anti-Mouse IgG (H+L), Alexa Fluor™ 555	Invitrogen	#A-31570
Donkey anti-Mouse IgG (H+L), Alexa Fluor™ Plus 647	Invitrogen	#A32787TR
Donkey anti-Rabbit IgG (H+L), Alexa Fluor™ 555	Invitrogen	#A-31572
Donkey anti-Rabbit IgG (H+L), Alexa Fluor™ Plus 647	Invitrogen	#A32795TR
Donkey anti-Goat IgG (H+L), Alexa Fluor™ 488	Invitrogen	#A-11055

Donkey anti-Goat IgG (H+L), Alexa Fluor™ 555	Invitrogen	#A-21432
Donkey anti-Rat IgG (H+L), Alexa Fluor™ Plus 647	Invitrogen	#A48272TR
Anti-OLIG2	Millipore	#ab9610
Anti-CXCL10	Novus	#AF-466-NA
Anti-Iba1 Guinea pig pAb	Oasis Biofarm	#OB-PGP049
Donkey-anti-Guinea pig IgG, AF488	Oasis Biofarm	#D-GP488
Anti-Arginase-1	Proteintech	#16001-1-AP
Anti-STAT3	Proteintech	#91408
Anti-β-Actin	Proteintech	#66009-1-Ig
Anti-GAPDH	Proteintech	#60004-1-g
HRP-Goat Anti-Mouse IgG	Proteintech	#SA00001-1
HRP-Goat Anti-Rabbit IgG	Proteintech	#SA00001-2
HRP-Rabbit Anti-Goat IgG	Proteintech	#SA00001-4
Anti-Ly6C	Santa Cruz	#sc-271811
Anti-CD69	Santa Cruz	#sc-373799
Anti-mouse PD-L1-InVivo	Selleck	#A2115
Rat IgG2b isotype control-InVivo	Selleck	#A2116
Anti-mouse CD8α-InVivo	Selleck	#A2102
Anti-p-STAT3 (Tyr705)	ZenBio	#R381552
Anti-p-STAT1 (Ser727)	ZenBio	#R25797
Anti-STAT1	ZenBio	#R25799
Anti-JAK2	ZenBio	#R24775
Anti-p-JAK2 (Tyr1007/1008)	ZenBio	#R381556
Anti-PIAS1	ZenBio	#R383086
Anti-PIAS3	ZenBio	#370213
Anti-SOCS2	ZenBio	#R25765
Anti-SOCS3	ZenBio	#500694
