# Supplementary figures and images for: Oligodendrocyte transcription factor 2 orchestrates glioblastoma immune evasion by suppressing CXCL10 and CD8+ T cell activation
Source: J Clin Invest. 2026 Jan 27;136(5):e195556. doi: 10.1172/JCI195556 (PMC12948422; doi:10.1172/JCI195556)

**Figure 4**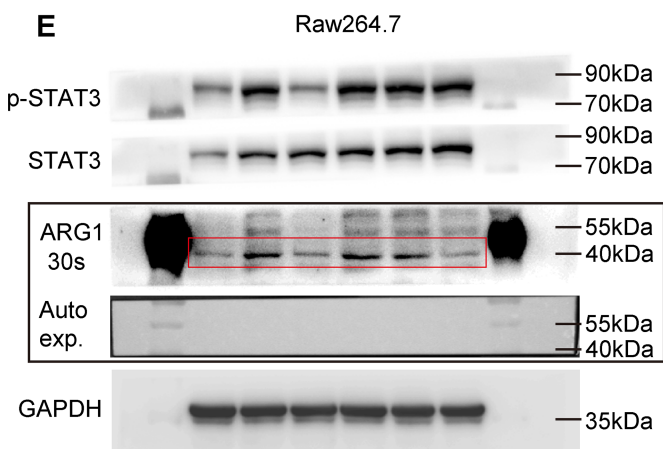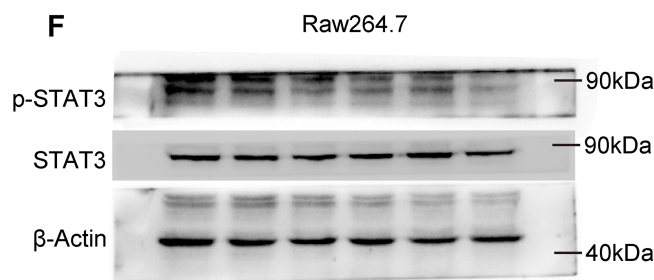**Figure 5**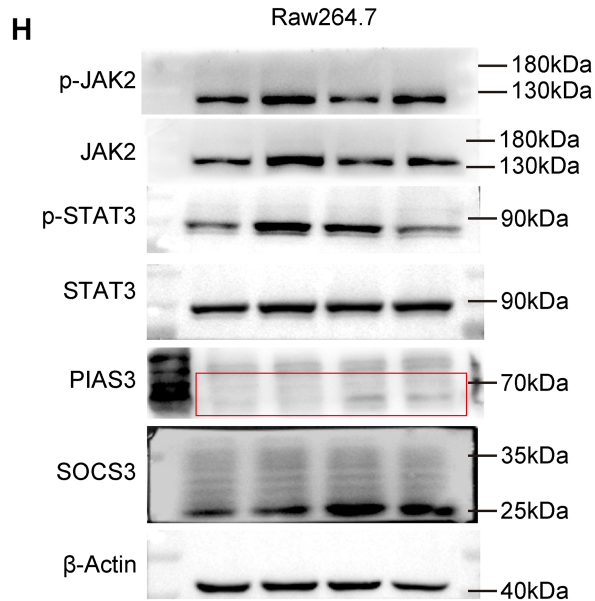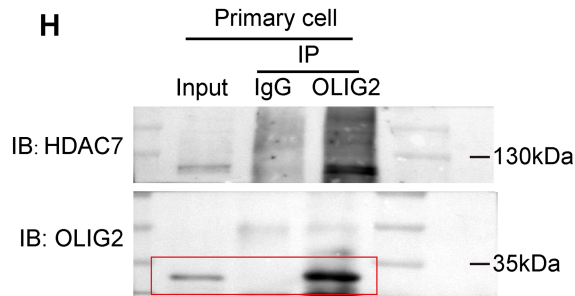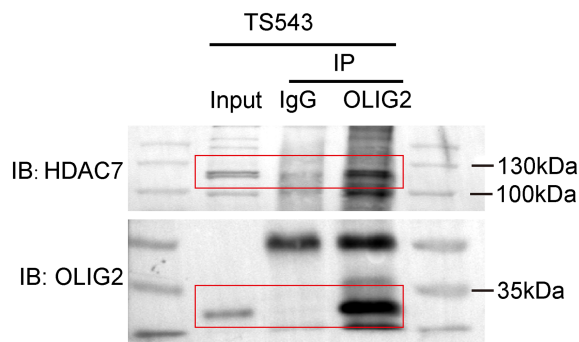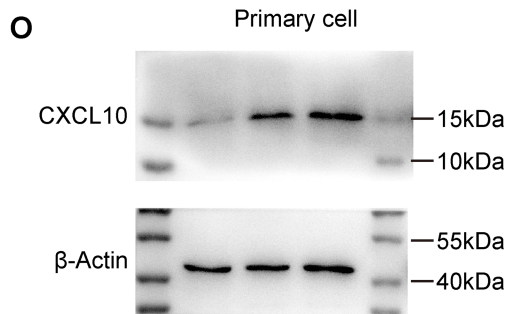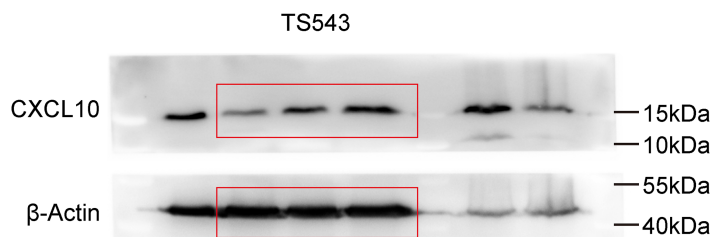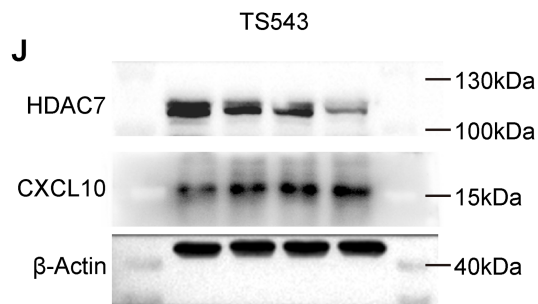

**Figure S3**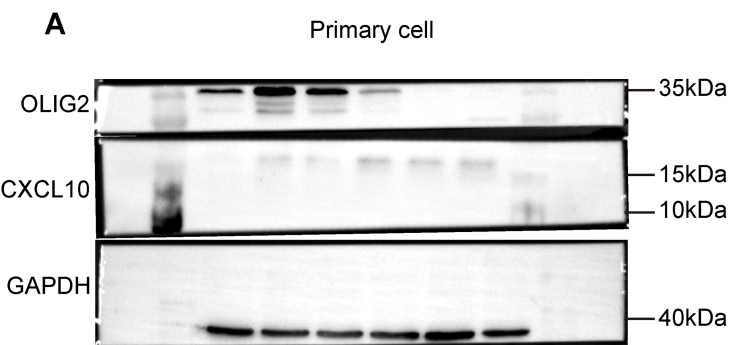**Figure S4**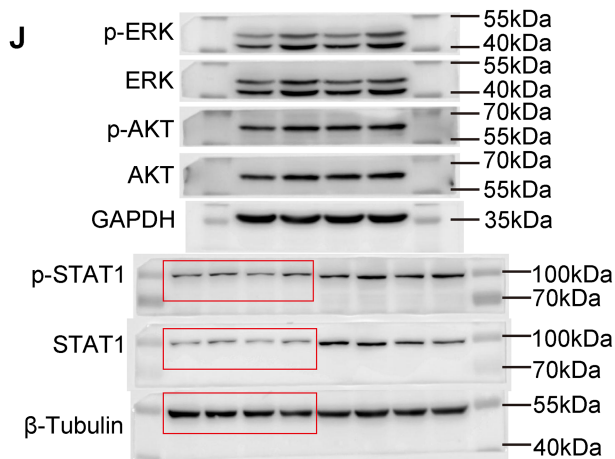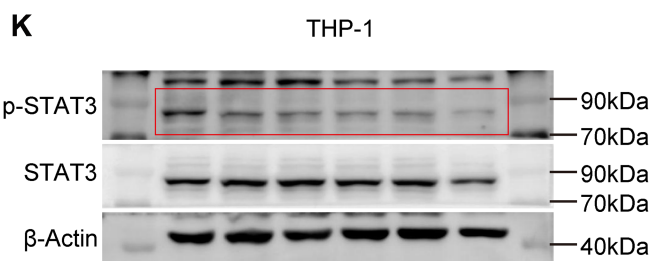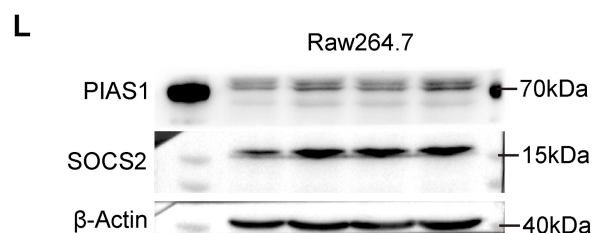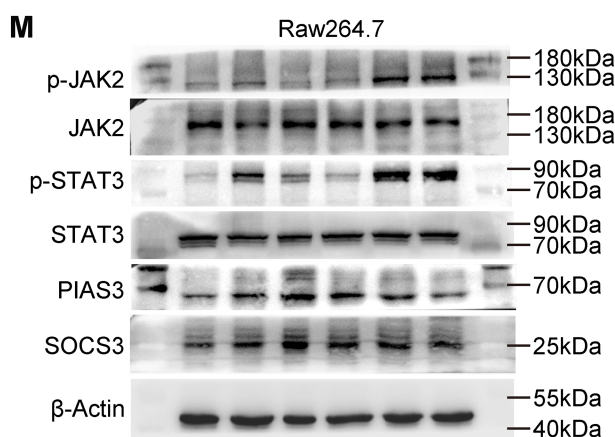**Figure S5**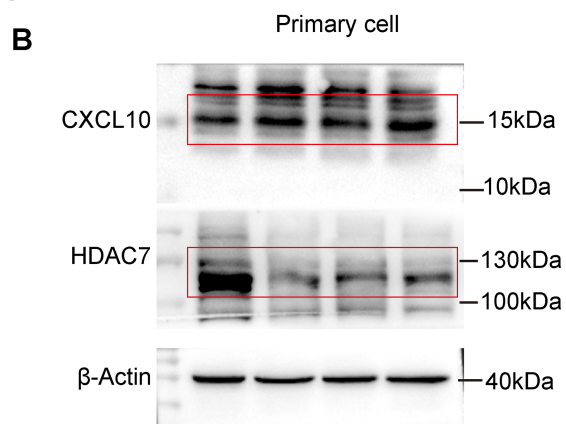**Figure S6**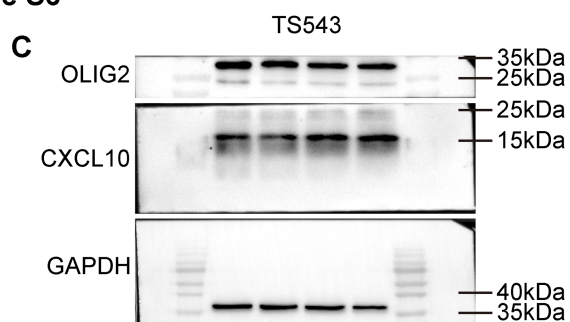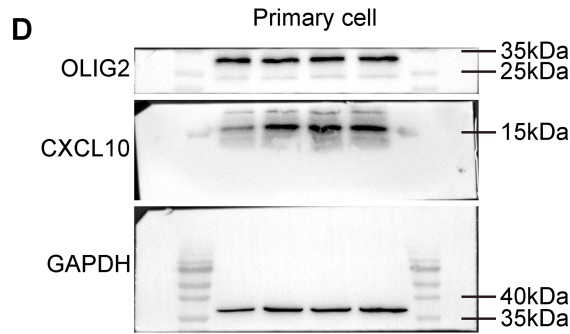

Supplement: Unedited blot and gel images [file jci-136-195556-s091.pdf]
