# Supplementary material for: Oligodendrocyte transcription factor 2 orchestrates glioblastoma immune evasion by suppressing CXCL10 and CD8+ T cell activation
Source: J Clin Invest. 2026 Jan 27;136(5):e195556. doi: 10.1172/JCI195556 (PMC12948422; doi:10.1172/JCI195556)
Supplement: Supplementary file 3 [file jci-136-195556-s008.pdf]

Oligodendrocyte transcription factor 2 orchestrates glioblastoma immune evasion by suppressing CXCL10 and $CD8^+$ T cell activation

Xinchun Zhang[1,2,#], Jinjiang Xue[1,3,#], Cunyan Zhao[1,4,#], Chenqiuyue Zeng[1,#], Jiacheng Zhong[2], Gangfeng Yu[1], Xi Yang[1], Yao Ling[3], Dazhen Li[1], Jiaxiao Yang[3], Yun Xiu[3], Hongda Li[3], Shiyuan Hong[5], Liangjun Qiao[3], Song Chen[2], Q. Richard Lu[6], Yaqi Deng[3,*], Zhaohua Tang[2,*], Fanghui Lu[1,*]

[1] Department of Cancer Center, The Second Affiliated Hospital of Chongqing Medical University, Chongqing Medical University, Chongqing, 404100, China.

[2] Department of Neurosurgery, Key Laboratory of Major Brain Disease and Aging Research (Ministry of Education), The First Affiliated Hospital of Chongqing Medical University, Chongqing, 400016, China.

[3]School of Basic Medical Sciences, Chongqing Medical University, Chongqing, 400016, China.

[4] Chongqing University Central Hospital, Chongqing, 400014, China.

[5] College of Pharmacy, Chongqing Medical University, Chongqing, 400016, China.

[6] Department of Pediatrics, Division of Experimental Hematology and Cancer Biology, Cincinnati Children's Hospital Medical Center, Cincinnati, OH, 45229, USA.

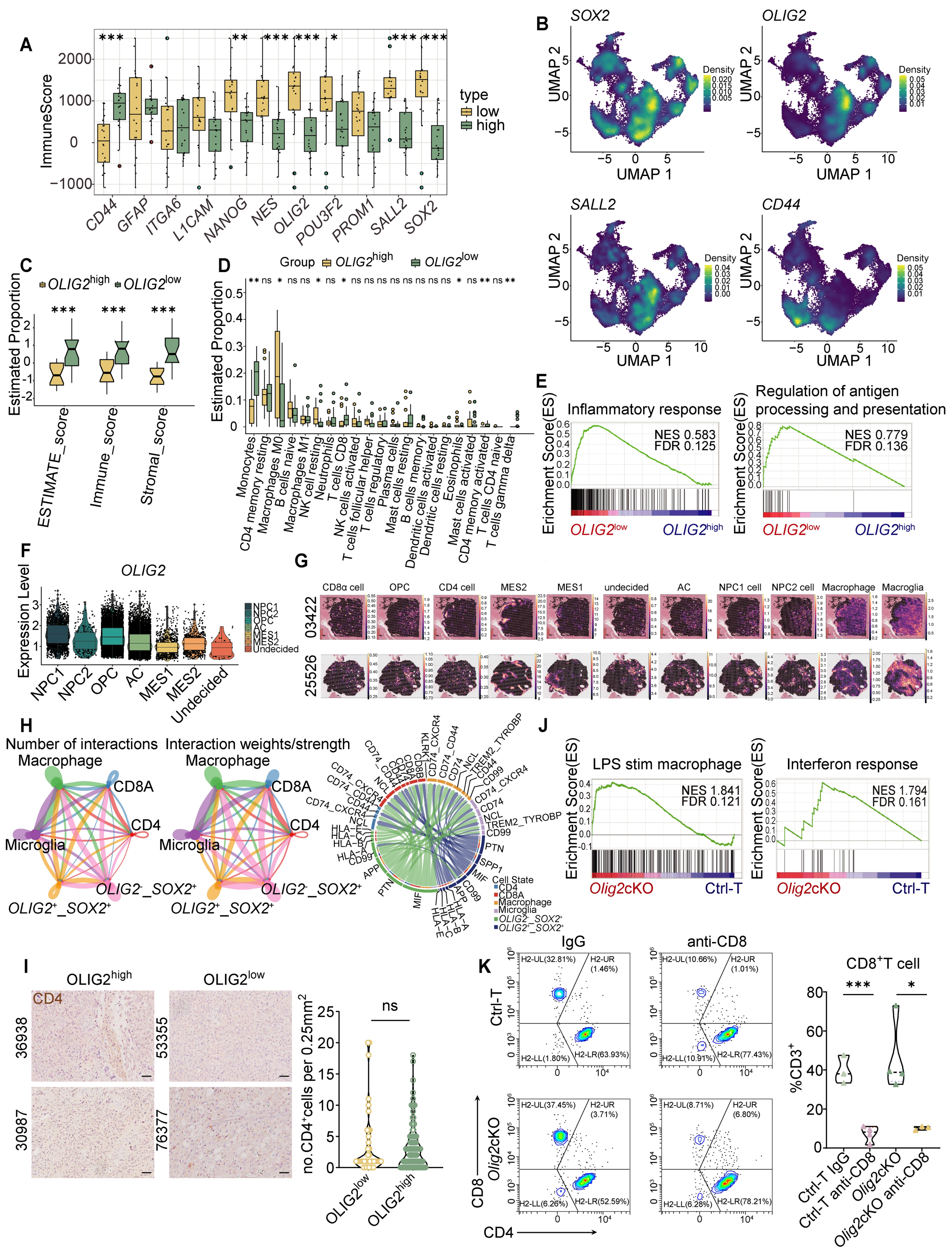
A
ImmuneScore
type
low
high
CD44
GFAP
ITGA6
L1CAM
NANOG
NES
OLIG2
POU3F2
PROM1
SALL2
SOX2
B
SOX2
OLIG2
SALL2
CD44
UMAP 1
UMAP 2
Density
C
Estimated Proportion
ESTIMATE_score
Immune_score
Stromal_score
D
Group
Estimated Proportion
E
Inflammatory response
NES 0.583
FDR 0.125
Regulation of antigen processing and presentation
NES 0.779
FDR 0.136
Erichment Score(ES)
F
OLIG2
Expression Level
NPC1
NPC2
OPC
AC
MES1
MES2
Undecided
G
CD8α cell
OPC
CD4 cell
MES2
MES1
undecided
AC
NPC1 cell
NPC2 cell
Macrophage
Macroglia
03422
25526
H
Number of interactions
Interaction weights/strength
Macrophage
CD8A
CD4
Microglia
Cell State
J
LPS stim macrophage
NES 1.841
FDR 0.121
Interferon response
NES 1.794
FDR 0.161
Olig2cKO
Ctrl-T
I
OLIG2high
OLIG2low
CD4
36938
53355
30987
76377
no.CD4+cells per 0.25mm2
ns
K
IgG
anti-CD8
Ctrl-T
Olig2cKO
CD8
CD4
CD8+T cell
%CD3+
Ctrl-T IgG
Ctrl-T anti-CD8
Olig2cKO
Olig2cKO anti-CD8

Supplementary Figure 1. High OLIG2 expression positively correlates with the pro-tumor immune microenvironment in GBM

(**A**) ESTIMATE analysis of immune infiltrations in GBM patients from TCGA database with high or low expression of GSC markers (n=40). (**B**) UMAP visualization of the specific GSC markers distribution in GBM cells (GSE182109). (**C**) ESTIMATE analysis of immune infiltrations in GBM patients from TCGA database with high or low *OLIG2* expression (n=40). (**D**) Characterization of the immune cell composition in GBM from TCGA databases by CIBERSORT analysis (n=40). (**E**) GSEA showing the enrichment of signature gene sets of immunoregulation in $OLIG2^{low}$ GBM cases compared with $OLIG2^{high}$ specimen (n=40). (**F**) The *OLIG2* expression levels in different subtypes of tumor cells (GSE182109). (**G**) Spatial feature plots showing the scores of cell types in GBM slides (GSE276841). (**H**) Cell-cell communications among $OLIG2^{+}SOX2^{+}$ GSC, $OLIG2^{-}SOX2^{+}$ GSC and immune cells from GBM scRNA-seq dataset (GSE182109) by CellChat. (**I**) Representative staining and quantification for CD4 (n=164) in GBM cases with high or low OLIG2 expression. Scale bar: 50 μm. Each plot represents the number of CD4 per area (0.25 mm^2). (**J**) GSEA showing the enrichment of signature gene sets of LPS-stimulated macrophage and Interferon response in GBM of *Olig2*cKO mice compared with Ctrl-T mice. (**K**) The efficiency of $CD8^+$ T cells depletion in mouse spleen (n=3-4 mice/group). Unpaired Student's t-test in **C**, **D**, **H** and **J**. *p< 0.05, **p < 0.01, ***p < 0.001, ns, not significant.

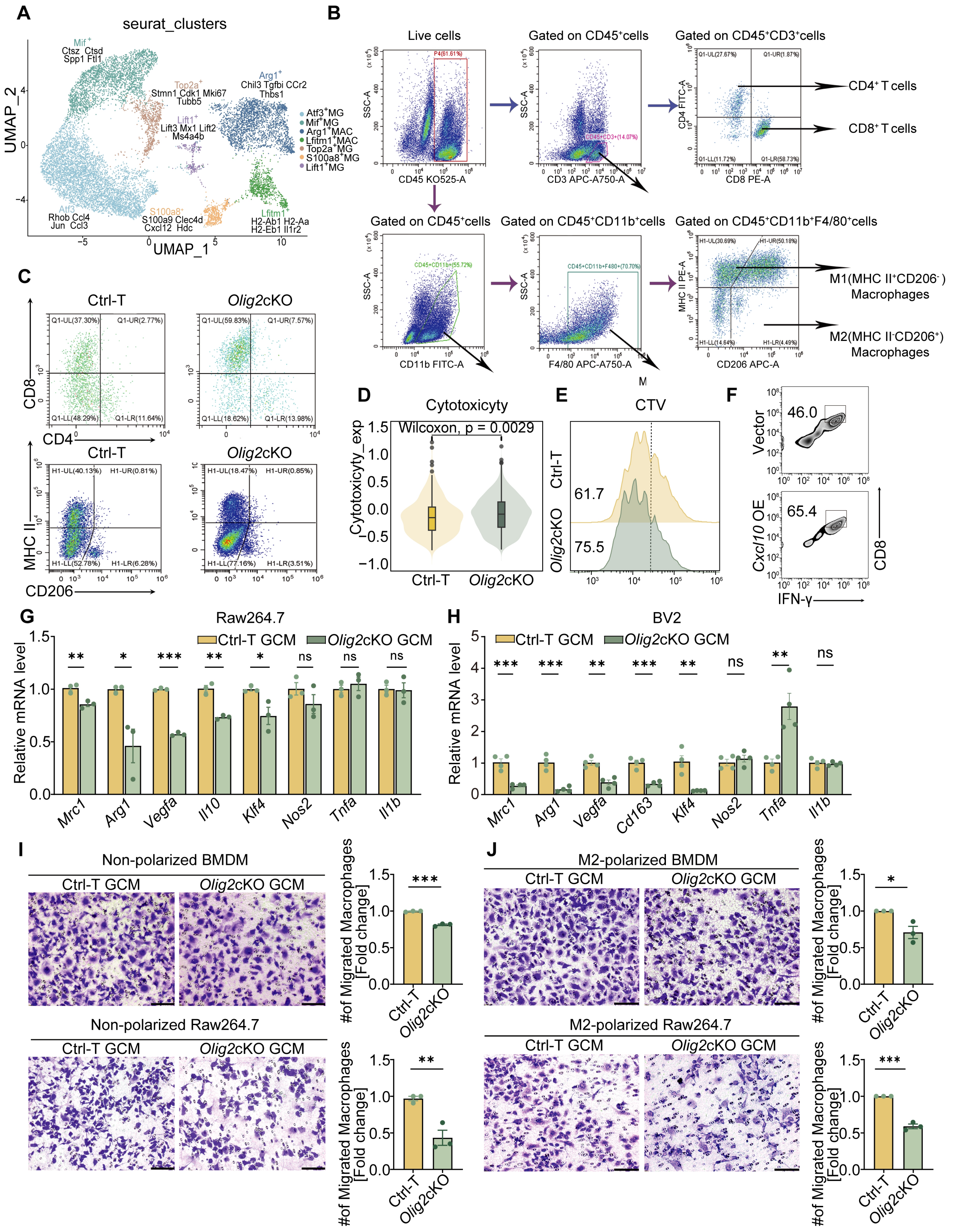

A
seurat_clusters
Mif+
Ctsz Ctsd
Spp1 Ftl1
Top2a+
Stmn1 Cdk1 Mki67
Tubb5
Arg1+
Chil3 Tgfbi CCr2
Thbs1
Lift1+
Lift3 Mx1 Lift2
Ms4a4b
Atf3
Rhob Ccl4
Jun Ccl3
S100a8+
S100a9 Clec4d
Cxcl12 Hdc
Lfitm1+
H2-Ab1 H2-Aa
H2-Eb1 Il1r2
Atf3+MG
Mif+MG
Arg1+MAC
Lfitm1+MAC
Top2a+MG
S100a8+MG
Lift1+MG
UMAP_2
UMAP_1
B
Live cells
Gated on CD45+cells
Gated on CD45+CD3+cells
SSC-A
CD45 KO525-A
CD3 APC-A750-A
CD4 FITC-A
CD8 PE-A
CD4+ T cells
CD8+ T cells
Gated on CD45+cells
Gated on CD45+CD11b+cells
Gated on CD45+CD11b+F4/80+cells
CD11b FITC-A
F4/80 APC-A750-A
MHC II PE-A
CD206 APC-A
M1(MHC II+CD206-)
Macrophages
M2(MHC II-CD206+)
Macrophages
C
Ctrl-T
Olig2cKO
CD8
CD4
MHC II
CD206
D
Cytotoxicyty
Wilcoxon, p = 0.0029
Cytotoxicyty_exp
Ctrl-T
Olig2cKO
E
CTV
Ctrl-T
Olig2cKO
61.7
75.5
F
Vector
46.0
Cxcl10 OE
65.4
CD8
IFN-γ
G
Raw264.7
Ctrl-T GCM
Olig2cKO GCM
Relative mRNA level
Mrc1
Arg1
Vegfa
Il10
Klf4
Nos2
Tnfa
Il1b
H
BV2
Ctrl-T GCM
Olig2cKO GCM
Relative mRNA level
Mrc1
Arg1
Vegfa
Cd163
Klf4
Nos2
Tnfa
Il1b
I
Non-polarized BMDM
Ctrl-T GCM
Olig2cKO GCM
Non-polarized Raw264.7
#of Migrated Macrophages
[Fold change]
Ctrl-T
Olig2cKO
J
M2-polarized BMDM
M2-polarized Raw264.7

41

Supplementary Figure 2. OLIG2 deletion enhances T cells activation and inhibits protumor TAMs in GBM

(**A**) UMAP of scRNAseq data from TAMs and signature genes for each TAM cluster were labeled. (**B**) Gating strategy for $CD8^+$ T cells, $CD4^+$ T cells, MHC II^-CD206^+ TAMs, and MHC II^+CD206^- TAMs in mouse GBM. (**C**) Representative flow cytometry scatter plots of the $CD8^+$ T cells, $CD4^+$ T cells, MHC II^-CD206^+ TAMs, and MHC II^+CD206^- TAMs in Ctrl-T and *Olig2*cKO mice. (**D**) Violin-box plots depicting the cytotoxicity score of the $CD8^+$ T cells using scRNA seq data from Ctrl-T and *Olig2*cKO groups. Two-tailed unpaired Wilcoxon rank-sum test. (**E**) Representative histograms for CellTrace Violet proliferation assay of $CD8^+$ T cells treated with CM from Ctrl-T and *Olig2*cKO tumor cells for 72h (n=3). (**F**) Representative plots for IFN-γ expression of $CD8^+$ T cells treated with CM from Ctrl-T and *Olig2*cKO tumor cells (n=3). (**G**, **H**) qPCR for mRNA expression of anti-tumor and pro-tumor markers in Raw264.7 and BV2 cells treated with Ctrl-T and *Olig2*cKO CM (n=3 in G; n=4 in H). (**I**, **J**) Representative images and quantifications of migrated non- or M2-polarized Raw264.7 and BMDMs toward Ctrl-T CM or *Olig2*cKO CM by Transwell assay (n=3). Scale bar: 100 μm. Unpaired Student's t-test in **G**-**J**. $*p < 0.05$, $**p < 0.01$, $***p < 0.001$, ns, not significant.

Supplementary figure 3

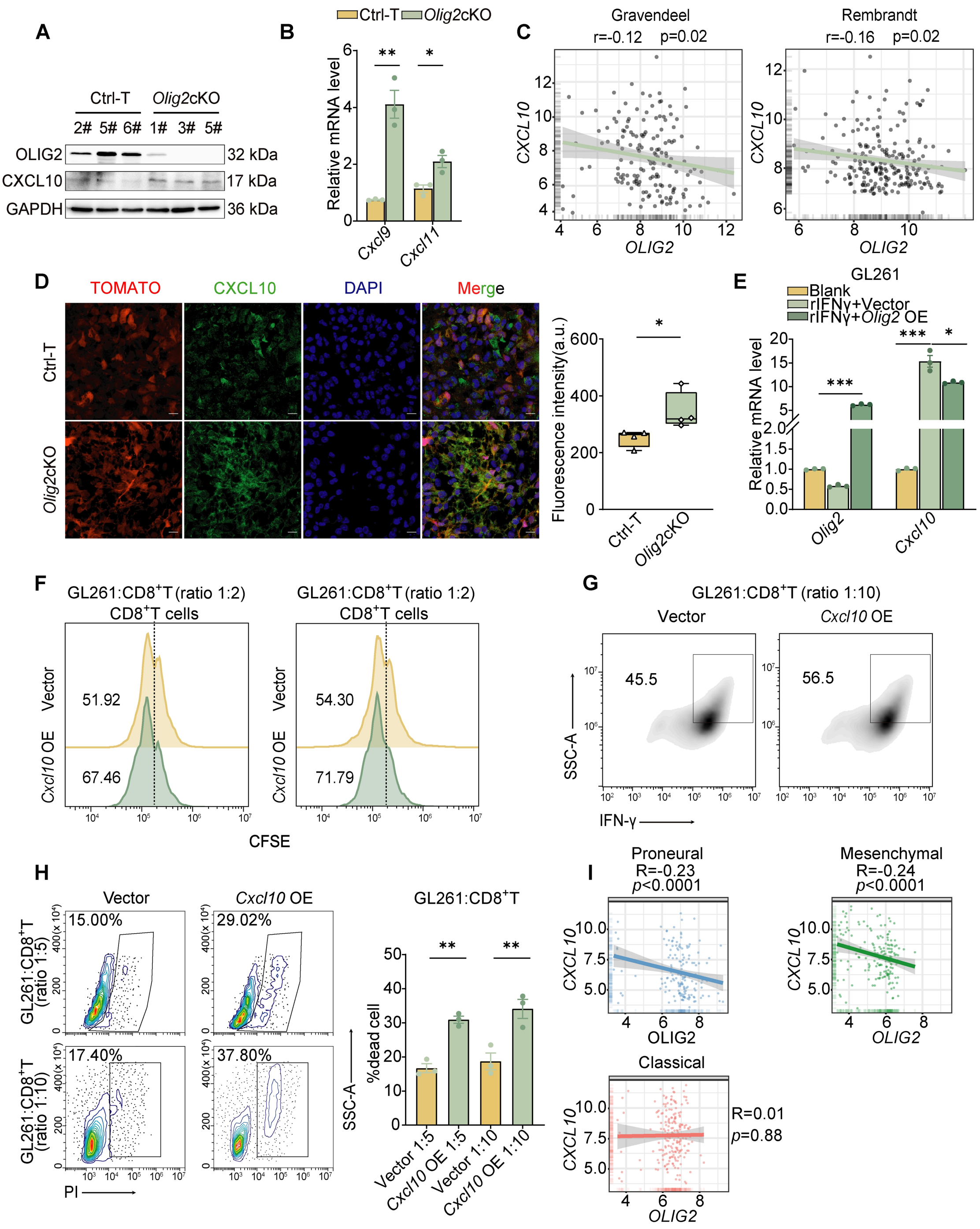

Supplementary Figure 3. OLIG2 regulates *CXCL10* expression to modulate T Cell proliferation and IFN-γ secretion

(**A**) Western blot for CXCL10 protein expression in Ctrl-T and *Olig2*cKO GBM cells. (**B**) qPCR for mRNA expression levels of *Cxcl9/11* in Ctrl-T and *Olig2*cKO GBM primary cells (*n*=3). (**C**) Correlation between *OLIG2* expression and *CXCL10* expression in Gravendeel and Rembrandt database. (**D**) Representative images and quantification of CXCL10 staining from Ctrl-T and *Olig2*cKO mice. Scale bar: 10 μm. (**E**) qPCR for mRNA expression of *Cxcl10* in 20ng/mL IFN-γ stimulated GL261 cells upon *Olig2* overexpression (*n*=3). (**F**) Representative histograms for CFSE proliferation assay of $CD8^+$ T cells co-cultured with GL261-Ctrl and GL261-*Cxcl10* OE cells at the indicated ratio for 72h (*n*=3). (**G**) Representative plots for IFN-γ expression of $CD8^+$ T cells treated with CM from GL261-Ctrl and GL261-*Cxcl10* OE cells for 24h (*n*=3). (**H**) Representative plots and quantification for the PI-positive tumor cells in GL261-Ctrl and GL261-*Cxcl10* OE groups cocultured with $CD8^+$ T cell at the indicated ratio for 72h (*n*=3). (**I**) Correlation between *OLIG2* and *CXCL10* expressions in TCGA GBM dataset. Unpaired Student's *t*-test in **B**, One-way ANOVA in **E, H**. $*p < 0.05$, $**p < 0.01$, $***p < 0.001$, ns, not significant.

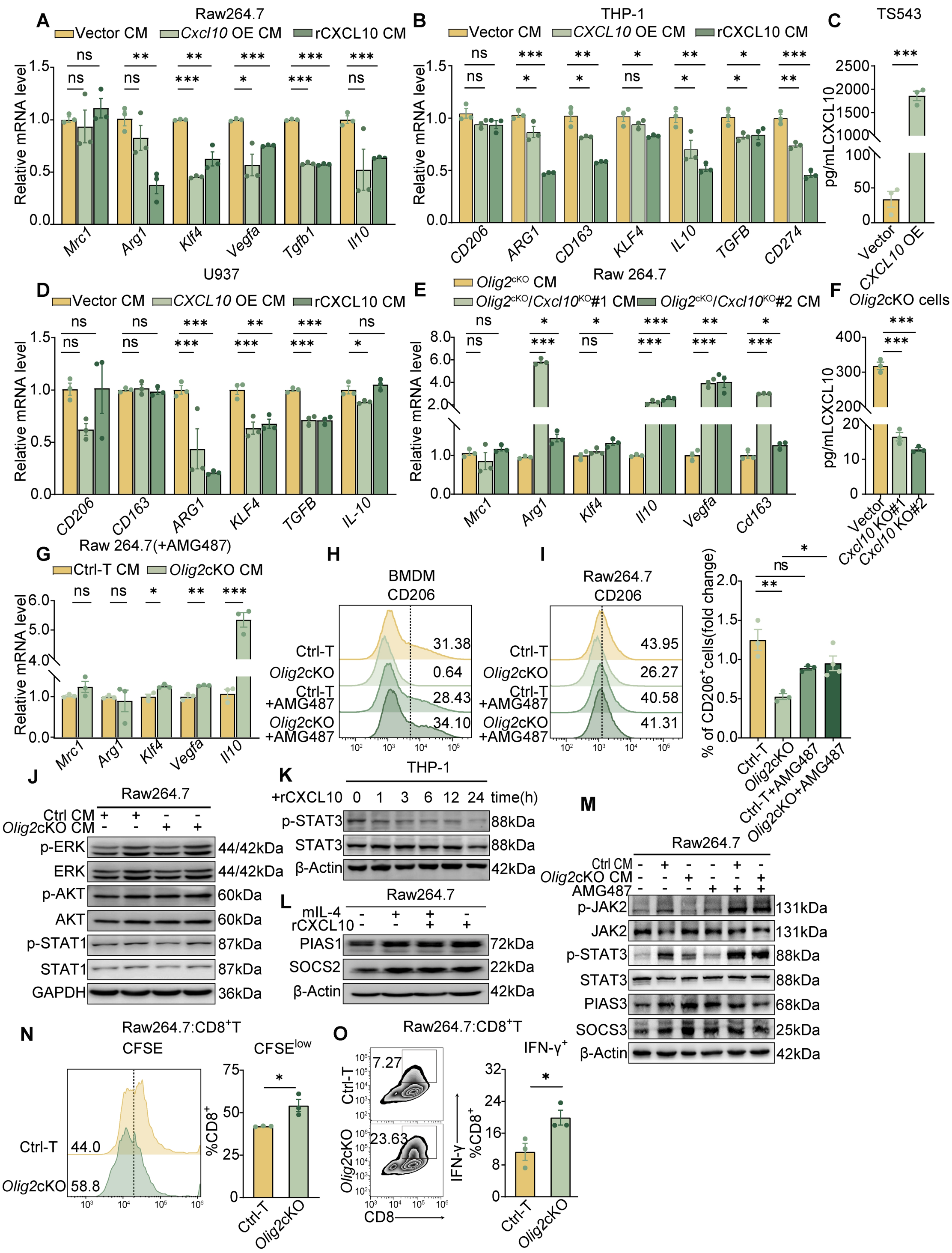
A
Raw264.7
Vector CM
Cxcl10 OE CM
rCXCL10 CM
Relative mRNA level
Mrc1
Arg1
Klf4
Vegfa
Tgfb1
Il10
B
THP-1
Vector CM
CXCL10 OE CM
rCXCL10 CM
Relative mRNA level
CD206
ARG1
CD163
KLF4
IL10
TGFB
CD274
C
TS543
pg/mLCXCL10
Vector
CXCL10 OE
D
U937
Vector CM
CXCL10 OE CM
rCXCL10 CM
Relative mRNA level
CD206
CD163
ARG1
KLF4
TGFB
IL-10
E
Raw 264.7
Olig2cKO CM
Olig2cKO/Cxcl10KO#1 CM
Olig2cKO/Cxcl10KO#2 CM
Relative mRNA level
Mrc1
Arg1
Klf4
Il10
Vegfa
Cd163
F
Olig2cKO cells
pg/mLCXCL10
Vector
Cxcl10 KO#1
Cxcl10 KO#2
G
Raw 264.7(+AMG487)
Ctrl-T CM
Olig2cKO CM
Relative mRNA level
Mrc1
Arg1
Klf4
Vegfa
Il10
H
BMDM
CD206
Ctrl-T 31.38
Olig2cKO 0.64
Ctrl-T +AMG487 28.43
Olig2cKO +AMG487 34.10
I
Raw264.7
CD206
Ctrl-T 43.95
Olig2cKO 26.27
Ctrl-T +AMG487 40.58
Olig2cKO +AMG487 41.31
% of CD206+cells(fold change)
Ctrl-T
Olig2cKO
Ctrl-T+AMG487
Olig2cKO+AMG487
J
Raw264.7
Ctrl CM
Olig2cKO CM
p-ERK 44/42kDa
ERK 44/42kDa
p-AKT 60kDa
AKT 60kDa
p-STAT1 87kDa
STAT1 87kDa
GAPDH 36kDa
K
THP-1
+rCXCL10 0 1 3 6 12 24 time(h)
p-STAT3 88kDa
STAT3 88kDa
β-Actin 42kDa
L
Raw264.7
mIL-4
rCXCL10
PIAS1 72kDa
SOCS2 22kDa
β-Actin 42kDa
M
Raw264.7
Ctrl CM
Olig2cKO CM
AMG487
p-JAK2 131kDa
JAK2 131kDa
p-STAT3 88kDa
STAT3 88kDa
PIAS3 68kDa
SOCS3 25kDa
β-Actin 42kDa
N
Raw264.7:CD8+T
CFSE
Ctrl-T 44.0
Olig2cKO 58.8
CFSElow
%CD8+
Ctrl-T
Olig2cKO
O
Raw264.7:CD8+T
Ctrl-T 7.27
Olig2cKO 23.63
CD8
IFN-γ+
IFN-γ
%CD8+
Ctrl-T
Olig2cKO

78

Supplementary Figure 4. CXCL10 attenuates M2 macrophage polarization through STAT3 signaling suppression

(**A**) qPCR for mRNA expression of anti-inflammatory markers in Raw264.7 cells treated with CM from the indicated groups for 48h (n=3). (**B**) qPCR for mRNA expression of anti-inflammatory markers in THP-1 cells treated with CM from the indicated groups for 48h (n=3). (**C**) ELISA for CXCL10 secretion levels in the supernatant of TS543-Vector and TS543-*CXCL10* OE cells (n=3). (**D**) qPCR for mRNA expression of anti-inflammatory markers in U937 cells treated with CM from the indicated groups for 48h (n=3). (**E**) qPCR for mRNA expression of anti-inflammatory markers in Raw264.7 cells treated with CM from the indicated groups for 48h (n=3). (**F**) ELISA for CXCL10 secretion levels in the supernatant of *Olig2*cKO and *Olig2*cKO-*Cxcl10* KO cells (n=3). (**G**) qPCR for mRNA expression of anti-inflammatory markers in Raw264.7 cells treated with Ctrl-T and *Olig2*cKO CM containing 1 μM AMG487 for 48h (n=3). (**H**) Representative histograms for the CD206 expression of BMDMs treated with Ctrl-T and *Olig2*cKO CM containing AMG487 for 24h (n=3). (**I**) Representative histograms and quantification for the CD206 expression of Raw264.7 cells treated with Ctrl-T and *Olig2*cKO CM containing AMG487 for 24h (n=3). (**J**) Western blot for the protein levels in Raw264.7 cells treated with Ctrl-T and *Olig2*cKO CM. (**K**) Western blot for the protein levels in THP-1 cells treated with 100 ng/mL rCXCL10 proteins at indicated time points. (**L**) Western blot for the protein levels in Raw264.7 cells treated with mIL-4 and rCXCL10 proteins. (**M**) Western blot for the protein levels in Raw264.7 cells co-cultured with Ctrl-T and *Olig2*cKO CM containing AMG487. (**N**) Representative histograms for CFSE proliferation assay of the CD8$^+$ T cells co-cultured with BMDMs pre-incubated with Ctrl-T and *Olig2*cKO CM (n=3). (**O**) Representative flow cytometry for the IFN-γ expression of CD8$^+$ T cells co-cultured with BMDMs pre-incubated with Ctrl-T and *Olig2*cKO CM (n=3). One-way ANOVA in **F**, **I**, Unpaired Student's t-test in **A**-**E**, **G**, **N**, **O**. *$p < 0.05$, **$p < 0.01$, ***$p < 0.001$, ns, not significant.

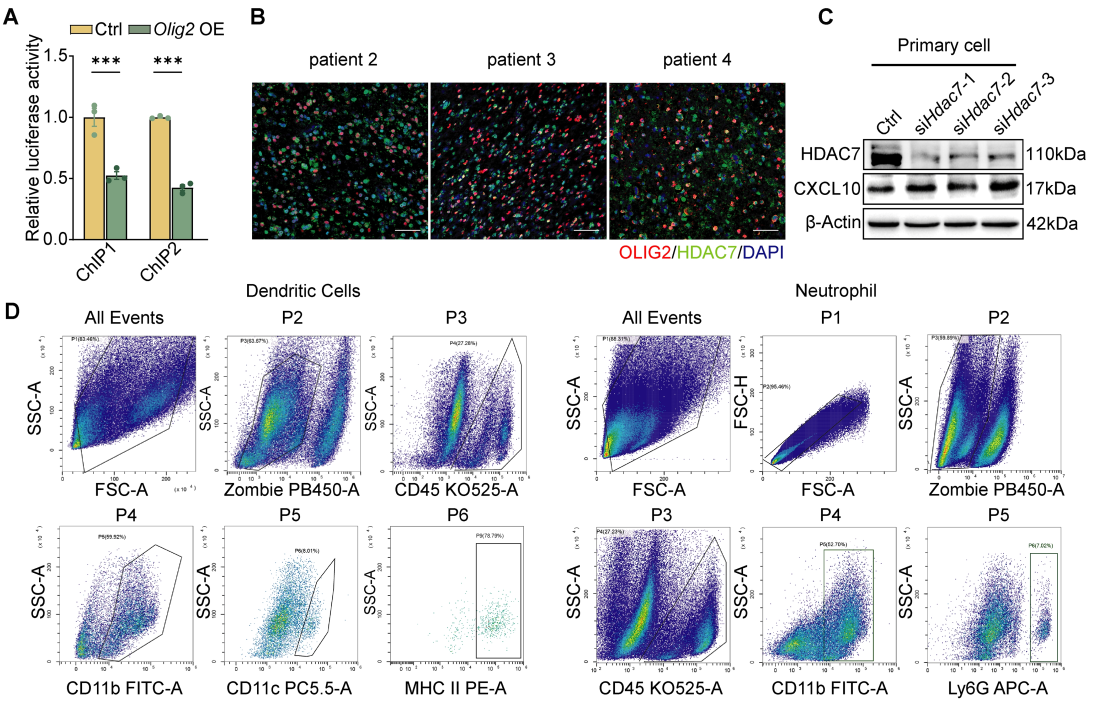

Supplementary Figure 5. OLIG2 recruits HDAC7 to epigenetically silence *CXCL10* expression

(**A**) Dual-luciferase reporter assay for *Cxcl10* enhancer activity in control or *Olig2* overexpressed 293FT cells (*n*=3). (**B**) Representative immunofluorescence images showing OLIG2 (red) and HDAC7 (green) signals from three GBM patient specimen. Scale bars = 50 μm. (**C**) Western blot for protein levels of HDAC7 and CXCL10 in Ctrl-T tumor cells transfected with si*Hdac7*. (**D**) Gating strategy for $CD11b^{+}CD11c^{+}MHC\ II^{+}$ dendritic cells and $CD11b^{+}Ly6G^{+}$ neutrophils in mouse GBM for Figure 2B. Unpaired Student's *t*-test in **A**. **p* < 0.05; ***p* < 0.01; ****p* < 0.001; ns, not significant.

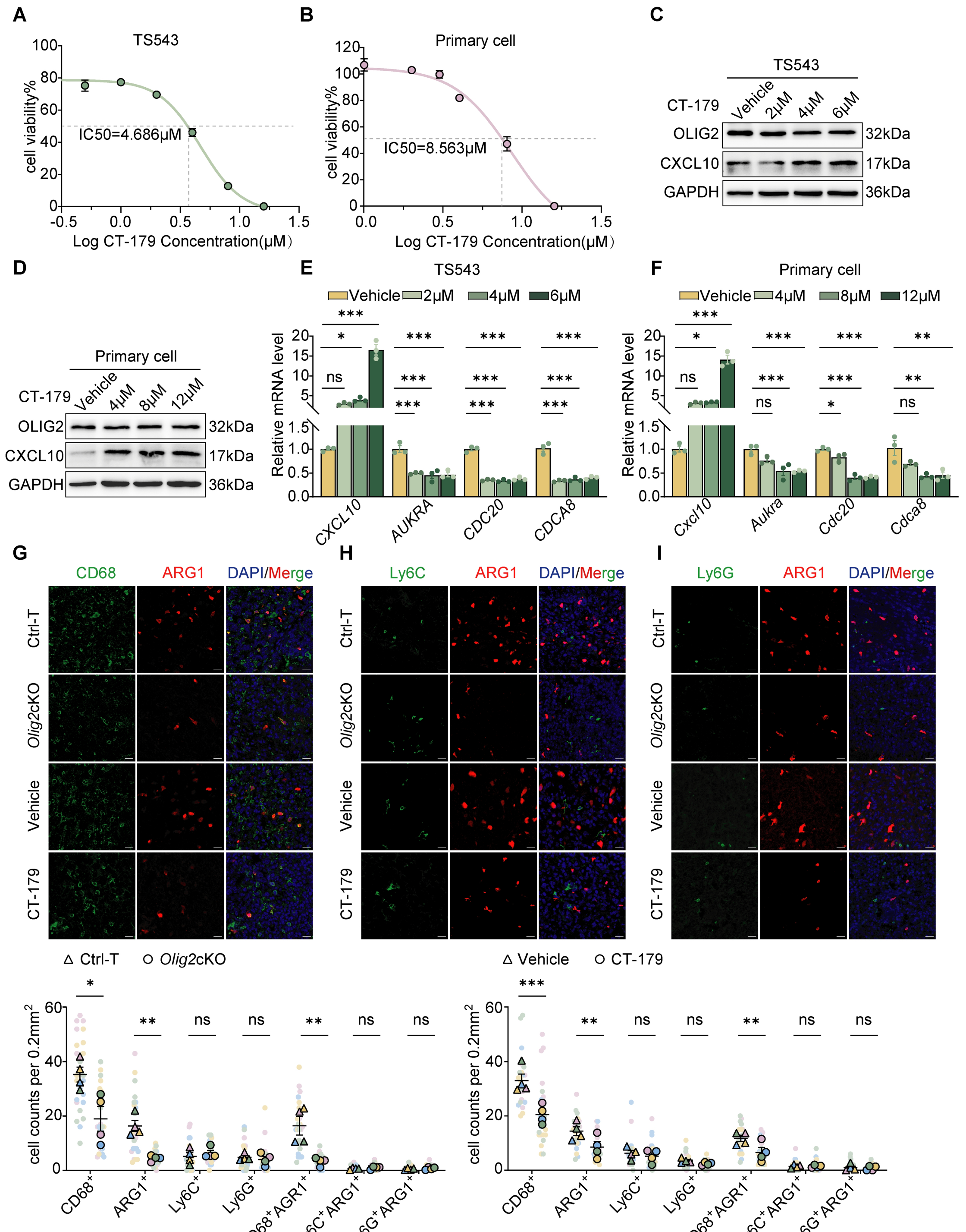
A
TS543
cell viability%
IC50=4.686μM
Log CT-179 Concentration(μM)
B
Primary cell
cell viability%
IC50=8.563μM
Log CT-179 Concentration(μM)
C
TS543
CT-179
Vehicle 2μM 4μM 6μM
OLIG2 32kDa
CXCL10 17kDa
GAPDH 36kDa
D
Primary cell
CT-179
Vehicle 4μM 8μM 12μM
OLIG2 32kDa
CXCL10 17kDa
GAPDH 36kDa
E
TS543
Vehicle 2μM 4μM 6μM
Relative mRNA level
CXCL10 AUKRA CDC20 CDCA8
F
Primary cell
Vehicle 4μM 8μM 12μM
Relative mRNA level
Cxcl10 Aukra Cdc20 Cdca8
G
CD68 ARG1 DAPI/Merge
Ctrl-T
Olig2cKO
Vehicle
CT-179
H
Ly6C ARG1 DAPI/Merge
I
Ly6G ARG1 DAPI/Merge
Ctrl-T Olig2cKO
Vehicle CT-179
cell counts per 0.2mm²
CD68⁺ ARG1⁺ Ly6C⁺ Ly6G⁺ CD68⁺AGR1⁺ Ly6C⁺ARG1⁺ Ly6G⁺ARG1⁺

120

Supplementary Figure 6. CT-179 suppresses OLIG2 transcriptional activity and reduces ARG1$^+$ TAMs in mouse GBM

(**A**, **B**) Cell viability assays for TS543 cells and mouse primary GBM cells exposed to CT-179 for 48h. (**C**, **D**) Western blot for OLIG2 and CXCL10 protein expressions in TS543 cells and mouses primary GBM cells treated with CT-179 for 48h. (**E**, **F**) qPCR for mRNA levels of the classical target genes of OLIG2 in TS543 GBM cells and mouse primary GBM cells treated with Vehicle or CT-179 for 48 hours (n=3). (**G-I**) (Top) Representative immunofluorescence images of tumor sections from four experimental groups: Ctrl-T, *Olig2*cKO, Vehicle, and CT-179 (20 mg kg^{-1}). CD68$^+$ macrophages (green), ARG1$^+$ cells (red) in **G**; Ly6C$^+$ monocytes (green), ARG1$^+$ cells (red) in **H**; Ly6G$^+$ neutrophils (green), ARG1$^+$ cells (red) in **I**. (Bottom) Quantitative analysis of immunosuppressive myeloid cell densities. Scale bars = 20 μm; n = 4 mice/group. Data was presented as mean ± SEM. One-way ANOVA in **E**, **F**. Unpaired Student's t-test in **G-I**, *p<0.05, **p<0.01, ***p<0.001, ns, not significant.

Supplementary Table 1

Name	Sequence
**Primers for qPCR**	
q-m*Il1b*-F	GCAACTGTTCCTGAACTCAACT
q-m*Il1b*-R	ATCTTTTGGGGTCCGTCAACT
q-m*Tnfa*-F	GACGTGGAACTGGCAGAAGAG
q-m*Tnfa*-R	TTGGTGGTTTGTGAGTGTGAG
q-m*Arg1*-F	CTCCAAGCCAAAGTCCTTAGAG
q-m*Arg1*-R	AGGAGCTGTCATTAGGGACATC
q-m*Il10*-F	GCTCTTACTGACTGGCATGAG
q-m*Il10*-R	CGCAGCTCTAGGAGCATGTG
q-m*Klf4*-F	GTGCCCCGACTAACCGTTG
q-m*Klf4*-R	GTCGTTGAACTCCTCGGTCT
q-m*Vegfa*-F	GCACATAGAGAGAATGAGCTTCC
q-m*Vegfa*-R	CTCCGCTCTGAACAAGGCT
q-m*Nos2*-F	GTTCTCAGCCCAACAATACAAGA
q-m*Nos2*-R	GTGGACGGGTCGATGTCAC
q-m*Cd163*-F	ATGGGTGGACACAGAATGGTT
q-m*Cd163*-R	CAGGAGCGTTAGTGACAGCAG
q-m*Tgfb1*-F	CTCCCGTGGCTTCTAGTGC
q-m*Tgfb1*-R	GCCTTAGTTTGGACAGGATCTG
q-m*β-Actin*-F	GGCTGTATTCCCCTCCATCG
q-m*β-Actin*-R	CCAGTTGGTAACAATGCCATGT
q-m*Mrc1*-F	CTCTGTTCAGCTATTGGACGC
q-m*Mrc1*-R	CGGAATTTCTGGGATTCAGCTTC
q-m*CD274*-F	GCTCCAAAGGACTTGTACGTG
q-m*CD274*-R	TGATCTGAAGGGCAGCATTTC
q-m*Vegfb*-F	GCCAGACAGGGTTGCCATAC
q-m*Vegfb*-R	GGAGTGGGATGGATGATGTCAG
q-m*Cxcl10*-F	CCAAGTGCTGCCGTCATTTTC
q-m*Cxcl10*-R	GGCTCGCAGGGATGATTTCAA
q-m*Cxcl9*-F	TCCTTTTGGGCATCATCTTCC
q-m*Cxcl9*-R	TTTGTAGTGGATCGTGCCTCG
q-m*Cxcl11*-F	GGCTTCCTTATGTTCAAACAGGG
q-m*Cxcl11*-R	GCCGTTACTCGGGTAAATTACA
q-m*Tnc*-F	ACGGCTACCACAGAAGCTG
q-m*Tnc*-R	ATGGCTGTTGTTGCTATGGCA
q-m*Il17ra*-F	AGTGTTTCCTCTACCCAGCAC
q-m*Il17ra*-R	GAAAACCGCCACCGCTTAC
q-m*Sema3e*-F	AGGCTACGCCTGTCACATAAA
q-m*Sema3e*-R	CCGTTCTTGATACTCATCCAGC
q-m*Adm*-F	CACCCTGATGTTATTGGGTTCA

q-m*Adm*-R	TTAGCGCCCACTTATTCCACT
q-H-*ACTIN*-F	CATGTACGTTGCTATCCAGGC
q-H-*ACTIN*-R	CTCCTTAATGTCACGCACGAT
q-H-*OLIG2*-F	GCTCCTCAAATCGCATCCA
q-H-*OLIG2*-R	AAAGGTCATCGGGCTCTG
q-H-*CXCL10*-F	GTGGCATTCAAGGAGTACCTC
q-H-*CXCL10*-R	TGATGGCCTTCGATTCTGGATT
q-H-*IL-10*-F	TCACATGCGCCTTGATGTCTG
q-H-*IL-10*-R	GACTTTAAGGGTTACCTGGGTTG
q-H-*TNFa*-F	TCGAGAAGATGATCTGACTGCC
q-H-*TNFa*-R	GCTGCACTTTGGAGTGATCG
q-H-*CD206*-F	TTTCTTGTCTGTTGCCGTAGTT
q-H-*CD206*-R	GGGTTGCTATCACTCTCTATGC
q-H-*Arg1*-R	CCAGTCCGTCAACATCAAAACT
q-H-*Arg1*-F	TGGACAGACTAGGAATTGGCA
q-H-*Klf4*-F	CGGACATCAACGACGTGAG
q-H-*Klf4*-R	GACGCCTTCAGCACGAACT
q-H-*TGFB1*-F	GGCCAGATCCTGTCCAAGC
q-H-*TGFB1*-R	GTGGGTTTCCACCATTAGCAC
q-H-*CD163*-F	TTTGTCAACTTGAGTCCCTTCAC
q-H-*CD163*-R	TCCCGCTACACTTGTTTTCAC
q-H-*CXCR3*-F	TTTGACCGCTACCTGAACATAGT
q-H-*CXCR3*-R	GGGAAGTTGTATTGGCAGTGG
q-H-*HDAC7*-F	GGCGGCCCTAGAAAGAACAG
q-H-*HDAC7*-R	CTTGGGCTTATAGCGCAGCTT
**sgRNA**	
Crispr1-m*Cxcl10* F	CACCGGCGGACCGTCCTTGCGAGA
Crispr1-m*Cxcl10* R	AAACTCCTGCGAGGAACGGTCCGCC
Crispr2-m*Cxcl10* F	CACCGGGGAGGGACGTTTATCGGCC
Crispr2-m*Cxcl10* R	AAACGCCGATAAACGTCCCTCCCC
**siRNA**	
m-si-*Hdac7*-1-s	GGCUGGAAACAGAAACCCA
m-si-*Hdac7*-1-a	UGGGUUUCUGUUUCCAGCC
m-si-*Hdac7*-2-s	CGGUCAUGCUGAAGCACCA
m-si-*Hdac7*-2-a	UGGUGCUUCAGCAUGACCG
m-si-*Hdac7*-3-s	GGAUAGUCGUGAUGCCCAU
m-si-*Hdac7*-3-a	AUGGGCAUCACGACUAUCC
H-si-*HDAC7*-1-s	GCAGUGUGGUCAAGCAGAA
H-si-*HDAC7*-1-a	UUCUGCUUGACCACACUGC
H-si-*HDAC7*-2-s	GGCAGGCUUACACCAGCAA
H-si-*HDAC7*-2-a	UUGCUGGUGUAAGCCUGCC

H-si-*HDAC7*-3-s	CCUGAAGUUGCGCUACAAA
H-si-*HDAC7*-3-a	UUUGUAGCGCAACUUCAGG

Supplementary Table 2

The information of antibody		
Antibodies	Source	Catalogue number
Anti-HLA-DMB	Abcam	#ab131273
Anti-Iba1	Abcam	#ab5076
Anti-Acetyl-Histone H3-K27	ABclonal	#A7253
Anti-HDAC7	ABclonal	#A13008
Anti-CXCL10	ABclonal	#A19138
Anti-β-Tubulin	ABclonal	#AC008
HRP-Mouse Anti-Rabbit IgG Light Chain	ABclonal	#AS061
PerCP/Cy5.5 anti-mouse CD8α(clone 53-6.7)	BD Biosciences	#561109
BV510 anti-mouse CD45 (clone 30-F11)	BioLegend	#103138
FITC anti-mouse/human CD11b (clone M1/70)	BioLegend	#101205
APC-Cy7 anti-mouse F4/80 (clone BM8)	BioLegend	#123117
APC anti-mouse CD206 (clone C068C2)	BioLegend	#141707
APC-Cy7 anti-mouse CD3 (clone 17A2)	BioLegend	#100222
APC anti-mouse CD4 (clone GK1.5)	BioLegend	#100405
PE-Cy7 anti-mouse PD-1 (clone RMP1-30)	BioLegend	#109109
APC anti-mouse IFN-γ (clone XMG1.2)	BioLegend	#505810
PerCP/Cy5.5 anti-mouse CD11c (clone N418)	BioLegend	#117327
BV605 anti-mouse NK1.1 (clone PK136)	BioLegend	#108739
APC anti-mouse Ly-6G (clone 1A8)	BioLegend	#127613
Purified anti-mouse CD3ε	BioLegend	#100340
Purified anti-mouse CD28	BioLegend	#117003
Zombie Violet	BioLegend	#423113
Anti-Ly6G	BioLegend	#127601
Anti-CD68	Bio-Rad	#MCA1957T
Anti-CD4	BOSTER	#A00344-2
Anti-CD8α	CST	#D8A8Y
Anti-CD8α	CST	#98941
Anti-p-ERK1/2	CST	#4370S
Anti-ERK	CST	#4695S
Anti-AKT	CST	#9272S
Anti-p-AKT	CST	#9271S
PE anti-mouse MHC II (clone M5/114.15.2)	Invitrogen	#12-5321-81
Rabbit IgG isotype control	Invitrogen	#02-6102
Donkey anti-Mouse IgG (H+L), Alexa Fluor™ Plus 488	Invitrogen	#A32766TR
Donkey anti-Mouse IgG (H+L), Alexa Fluor™ 555	Invitrogen	#A-31570
Donkey anti-Mouse IgG (H+L), Alexa Fluor™ Plus 647	Invitrogen	#A32787TR
Donkey anti-Rabbit IgG (H+L), Alexa Fluor™ 555	Invitrogen	#A-31572
Donkey anti-Rabbit IgG (H+L), Alexa Fluor™ Plus 647	Invitrogen	#A32795TR
Donkey anti-Goat IgG (H+L), Alexa Fluor™ 488	Invitrogen	#A-11055

Donkey anti-Goat IgG (H+L), Alexa Fluor™ 555	Invitrogen	#A-21432
Donkey anti-Rat IgG (H+L), Alexa Fluor™ Plus 647	Invitrogen	#A48272TR
Anti-OLIG2	Millipore	#ab9610
Anti-CXCL10	Novus	#AF-466-NA
Anti-Iba1 Guinea pig pAb	Oasis Biofarm	#OB-PGP049
Donkey-anti-Guinea pig IgG, AF488	Oasis Biofarm	#D-GP488
Anti-Arginase-1	Proteintech	#16001-1-AP
Anti-STAT3	Proteintech	#91408
Anti-β-Actin	Proteintech	#66009-1-Ig
Anti-GAPDH	Proteintech	#60004-1-g
HRP-Goat Anti-Mouse IgG	Proteintech	#SA00001-1
HRP-Goat Anti-Rabbit IgG	Proteintech	#SA00001-2
HRP-Rabbit Anti-Goat IgG	Proteintech	#SA00001-4
Anti-Ly6C	Santa Cruz	#sc-271811
Anti-CD69	Santa Cruz	#sc-373799
Anti-mouse PD-L1-InVivo	Selleck	#A2115
Rat IgG2b isotype control-InVivo	Selleck	#A2116
Anti-mouse CD8α-InVivo	Selleck	#A2102
Anti-p-STAT3 (Tyr705)	ZenBio	#R381552
Anti-p-STAT1 (Ser727)	ZenBio	#R25797
Anti-STAT1	ZenBio	#R25799
Anti-JAK2	ZenBio	#R24775
Anti-p-JAK2 (Tyr1007/1008)	ZenBio	#R381556
Anti-PIAS1	ZenBio	#R383086
Anti-PIAS3	ZenBio	#370213
Anti-SOCS2	ZenBio	#R25765
Anti-SOCS3	ZenBio	#500694
